# Expression prevalence and dynamics of GPCR somatostatin receptors 2 and 3 as cancer biomarkers beyond NET: a paired immunohistochemistry approach

**DOI:** 10.1038/s41598-023-47877-0

**Published:** 2023-11-27

**Authors:** Mor Oron-Herman, David Kirmayer, Amelie Lupp, Stefan Schulz, Genady Kostenich, Michel Afargan

**Affiliations:** 1 Starget Pharma, 26 Snir st., 4704086 Ramat Hasharon, Israel; 2 Institute of Pharmacology and Toxicology, Jena University Hospital, Friedrich Schiller University Jena, Drackendorfer Str. 1, 07747 Jena, Germany; 3 The Advanced Technology Center, Sheba Medical Center, Tel Hashomer, 5262000 Ramat Gan, Israel

**Keywords:** Immunohistochemistry, Tumour biomarkers, High-throughput screening

## Abstract

Somatostatin receptors are clinically validated GPCR biomarkers for diagnosis and treatment of various neuroendocrine tumors (NET). Among the five somatostatin receptors, SST2 and SST3 are associated with apoptosis and cell cycle arrest, making these receptor subtypes better differentiated targets in precision oncology. In this study we performed immunohistochemistry of paired tissue microarrays containing 1125 cores, representing 43 tumor types, each stained for SST2 and SST3. A 12-point immunoreactive scoring (IRS) range was used for interpretation of the staining results. We analyzed the results twice, using the conventional positivity IRS cutoffs ≥ 3 and more stringent ≥ 6. Evaluation of receptors expression dynamics was performed for tumor-nodes-metastases (TNM) defined subgroups (ovarian and hepatocellular adenocarcinomas) as a function of their tumor stage. Our results indicate that two-thirds of tested cores exhibit clinically significant expression of at least SST2 or SST3 (IRS ≥ 6). The expression prevalence of both receptors tends to decline with tumor progression. However, an unexpected upregulation of both SST2 and SST3 reemerged in metastases suggesting conserved receptors genetic potential during tumor life cycle. We suggest that SST2 and SST3 should be further explored as potential biomarkers and therapeutic tools for maximizing the efficiency of somatostatin-based precision oncology of solid tumors beyond NET.

## Introduction

Clinically validated biomarkers specific to cancer cells are hallmarks of diagnosis and treatment in precision oncology. Molecular specific treatment modalities aim to improve the relatively narrow therapeutic index of traditional chemotherapy and radiation. Targeted molecular oncology approach is based on targeting tumoral foci via genetic, functional, or structural cellular mechanisms that are highly specific to cancerous compared to the normal tissues. Breast cancer was one of the first malignancies for which targeted therapies have been applied successfully by hormone based targeting and monoclonal antibody against human epidermal growth factor receptor 2 (HER2)^1–3^. Hence, the discovery and validation of various cancer-specific biomarkers drive the development of novel targeted therapeutics.

G-protein-coupled receptors (GPCRs) are considered the largest and the most diverse group of membrane-bound receptors. They are involved in a multitude of physiological processes and serve as clinically validated targets for diagnosis and therapy of a wide range of diseases^4^. By 2021, over one-third^5^ of all Food and Drug Administration (FDA) approved drugs targeted 108 members of the GPCR family. Several GPCR family members are also known to be involved in tumorigenesis by modulating proliferative signaling, replicative immortality, evasion of growth suppressors, resistance to apoptosis, initiation of angiogenesis, and activation of invasion and metastasis^6^. Complex cellular trafficking, interactions with other cellular components (inter and intrafamily), homo- and hetero-dimerization, and eventful life cycle of these receptors make them desirable yet challenging targets.

Somatostatin receptors (SST) belong to the GPCR superfamily and are encoded by five highly conserved genes (sst1-sst5), each located in a distinct chromosome^7^. Two variants of SST2: SST2A, and SST2B, are generated by alternative splicing^8^. Intrafamily, the SST subtypes differ from one another in several structural and functional aspects in health and disease^9^. Overexpression of SSTs was reported in a wide range of malignancies compared to their expression in normal cells^10–12^. Therefore, somatostatin analogs were the first-in-class receptor-binding ligands that gained clinical application in precision oncology for the diagnosis and treatment of cancer^12–15^. Recently, the highly selective SST2 radioligands of DOTATATE, [^68^ Ga]Ga-DOTA-TATE (Netspot™) and [^177^Lu]Lu-DOTA-TATE (Lutathera™), were approved for both diagnosis and treatment of gastroenteropancreatic neuroendocrine tumors (GEP-NET) that overexpress somatostatin receptor 2A (SST2A^+^ GEP-NET)^16,17^. Currently, several clinical trials are ongoing, aiming to explore the potential of SST2 for both diagnostic and therapeutic application for additional cancer indications beyond GEP-NET^18–24^. This additional clinical validation of SSTs as molecular targets in cancer fuels numerous efforts to explore somatostatin analogs conjugated to diagnostic or therapeutic moieties^25^.

Somatostatin receptors expression, like that of any other biomarker, can be detected by different in-vivo and ex-vivo techniques, such as scintigraphy, autoradiography, reverse-transcriptase polymerase chain reaction (RT-PCR), Western blot, *in-situ* hybridization, and immunohistochemistry (IHC)^26–29^. Naturally, these techniques aim at different biological targets related to biomarker expression, and unsurprisingly, different methods may furnish different results, depending not just on the level of expression, but also translation, membrane trafficking, turnover, and other cellular processes. Moreover, the inherent variability in the tissue samples, individuals, and protocol differences may lead to dissimilar results even when using the same methodology. Hence, high prevalence results obtained by any of these in vitro/ex vivo methods do not necessarily guarantee clinically relevant intensity, therefore, reports of prevalence of certain SST subtypes in a particular tumor should be routinely considered with caution.

Of these methods, IHC is widely used, both in research and in clinical settings. However, the reproducibility of this empirical methodology is frequently limited by numerous uncontrolled factors, and each assay should be validated for its respective biomarker and primary antibody. This lack of standardization, both in the procedural and the evaluating stages, i.e. interpreting and reporting of results, makes the comparison of different reports complicated and challenging^30^. In fact, although the IHC is abundantly used, to date, only three biomarkers have gained the privilege of approved standard methodology: Her2/neu, estrogen receptor (ER), and progesterone receptor (PR)^31^. Interpretation of the IHC data is made using several surrogate scales corresponding to various levels of target expression. One such scale is the 12-point prevalence-intensity immunoreactive score (IRS). Originally, the interpretation of IRS values was classified as follows: 0–1 indicates negative, and ≥ 2 indicates positive expression. Specifically: 2–3 score indicated mild expression, 4–8 score indicated moderate expression, and 9–12 score indicated strong expression^32^. This classification has been slightly shifting during the last decade towards revisited interpretation. While there is a consensus regarding what is considered strong staining, the positivity threshold was elevated from 2 to 3, and the interim definitions of mild and moderate expression were shifted accordingly: 0–2, negative/no expression; 3–5, mild expression; 6–8, moderate expression; and 9–12, strong expression^33^. Moreover, a recent study indicated that considering mild expression as positive may be misleadingly low in terms of its clinical relevance^34^.

Much work has been performed and published on the expression of SSTs in human malignancies, including a body of IHC work using various protocols^11,26^. Investigating the correlation of SST2 immunohistochemical scoring versus PET scans of [^68^ Ga]Ga-DOTATATE tracer in GEP-NET patients, Yu et al.^34^ found that the highest sensitivity and specificity of IRS in predicting the imaging results were obtained when defining the positivity cutoff value as 6, meaning that 51–80% of the tumor cells should be moderately stained or 10–50% should be strongly stained.

Several publications describing new sets of selective and specific antibodies targeting SSTs appeared around a decade ago^35–39^ prompting reassessment of various somatostatin receptor subtypes’ expression^40–44^. Nevertheless, discrepancies between the new IHC findings, the other methods, and the earlier data were also reported^45^. Moreover, a specific evaluation of rabbit anti-SST3 and anti-SST5 antibodies concluded that SST3 IHC staining is not yet optimal and should be applied with caution^46^.

Whereas SST2A is a clinically validated target that has been successfully employed in the treatment of neuroendocrine neoplasms, targeted drug delivery, and theranostics, the definite subtype 3 data is still elusive, although recent studies indicate that it might mature into another viable therapeutic target^47–50^. Therefore, it was our aim to generate a broad, coherent set of IHC data on multiple human cancers by implementing the tissue microarray (TMA) technique to evaluate both SST2A and SST3 expression in paired specimens. To the best of our knowledge, this is the first attempt to evaluate a wide range of cancer tissues by unified staining protocols and evaluation for these two receptor subtypes.

## Results

**Figure 1:**
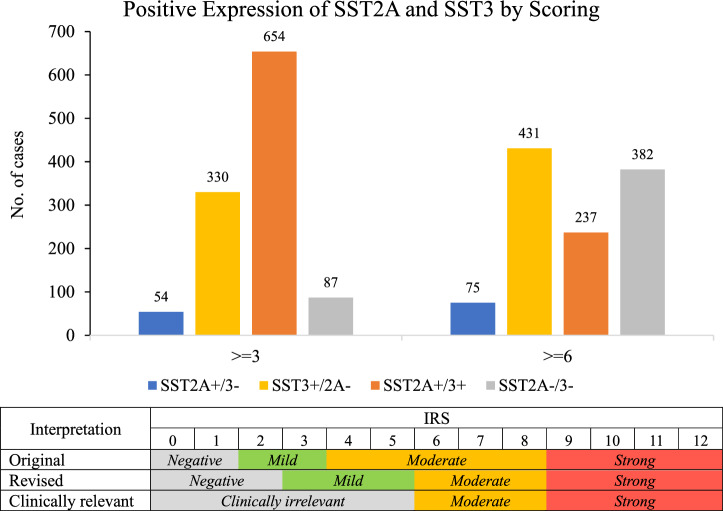
Distribution of positive expression of SST2A and SST3 in the tested cohort as detected by IRS conventional interpretation (≥ 3) and clinically relevant (≥ 6) method. The pane below presents methods of interpretation of IRS results as used today.

A total of 1,125 cores from 10 commercially available human tissue microarrays representing 964 patients pertaining to 43 clinical conditions were included in this study. For the sake of consistency and traceability, the manufacturer’s terminology is used herein. Further, seven additional cores were omitted from the analysis as they were damaged or otherwise unevaluable. All the cores were analyzed per each receptor in parallel under the same experimental conditions. The microarrays included a wide variety of cancerous tissues grouped according to the oncological conditions (Table 1). The summarized data on the expression of somatostatin receptors type 2A, and 3 is presented in Tables 2 and 3. Representative images of SST2A and SST3 stained tissues are shown in the Supplementary Information Figs. SI-1, SI-2 and SI-3. Evaluation of each receptor expression was done using the standard IRS scale, obtained by multiplying IHC staining intensity factor by fraction of stained cells factor (Table SI-2, see also “Methods” section, Scoring). A schematic illustration of three interpretation methods is presented in the lower pane of Fig. 1.

**Table 1:** Description of commercially available TMAs that were used in the present study.

TMA # (cases/cores)	Description of content
LY301 (30/30)	Lymph-node diffuse large B cell lymphoma (DLBCL) tissue microarray
TP242f (24/24)	Top 4 types with normal tissue array, including colon adenocarcinoma, breast invasive carcinoma of no special type, prostate adenocarcinoma, lung squamous cell carcinoma, lung adenocarcinoma
NE841 (46/74)	Multiple organs neuroendocrine tumor tissue microarray, containing 20 × 2 cases neuroendocrine carcinomas (carcinoid, atypical carcinoid, and small cell carcinoma), 2 squamous cell carcinoma, 16 adenocarcinoma, 8 normal tissues
AG801 (80/80)	Adrenal tumor tissue microarray, containing 10 cases of adrenal cortical adenocarcinoma, 30 pheochromocytoma, 40 adrenocortical adenoma
LV1221 (100/112)	Liver primary carcinoma and metastatic carcinoma tissue microarray containing 5 cases of cancer adjacent liver tissue, 39 intrahepatic cholangiocarcinoma, 28 hepatocellular carcinoma, 3 carcinoid, 2 adenosquamous carcinoma, 4 mixed carcinoma, 19 metastatic carcinoma
LV1021a (102/102)	Liver hepatocellular carcinoma with liver tissue microarray, containing 97 cases of hepatocellular carcinoma, 4 liver cirrhosis, and 1 liver tissue
BO2081 (104/208)	Bone disease spectrum and cancer adjacent normal bone tissue microarray, containing × 2 of 25 cases of osteosarcoma, 10 chondrosarcoma, 9 myeloma, 2 Ewing's sarcoma, 2 chordoma, 1 parosteal osteosarcoma, 20 giant cell tumor of bone, 8 adamantinoma, 1 osteoblastoma, 2 chondroma, 4 osteochondroma, 3 osteofibrous dysplasia, 6 bone cyst, 10 adjacent normal bone tissue
BC000120b (190/190)	Multiple organ carcinoma tissue microarray, containing 38 cases each of stomach adenocarcinoma, hepatocellular carcinoma, ovary adenocarcinoma, endometrioid adenocarcinoma and squamous cell carcinoma of head and neck
KD2001 (100/200)	Kidney tumor with normal tissue microarray, containing 71 cases of clear cell carcinoma, 13 invasive low grade urothelial carcinoma, 10 normal kidney tissue, plus 2 each of sarcomatoid carcinoma, papillary renal cell carcinoma and chromophobe carcinoma
ME2082d (188/192)	Malignant melanoma with skin tissue microarray, containing 112 cases malignant melanoma, 64 metastatic malignant melanoma (4 cases matched with malignant melanoma), 8 adjacent normal skin tissue, 8 skin tissue

**Table 2:** Expression of SST2A and SST3 in conditions represented by more than 20 cores.

Diagnosis	TMA ID	SST2A^+^/3^–^ % (n)	SST3^+^/2A^–^ % (n)	SST2A^+/^3^+^ % (n)	SST2A^–^/3^–^ % (n)
Bone giant cell tumor (n = 40)	BO2081	2.5% (1)	5.0% (2)	85.0% (34)	7.5% (3)
Adrenocortical adenoma (n = 40)	AG801	5% (2)	22.5% (9)	65% (26)	7.5% (3)
Osteosarcoma (n = 52)	BO2081	7.7% (4)	48.1% (25)	34.6% (18)	9.6% (5)
Malignant melanoma (n = 176)	ME2082d	2.3% (4)	44.9% (79)	43.2% (76)	9.7% (17)
Benign pheochromocytoma (n = 30)	AG801	0.0% (0)	56.7% (17)	30.0% (9)	13.3% (4)
Metastatic hepatocellular carcinoma (n = 34)	LV1221	5.9% (2)	52.9% (18)	20.6% (7)	20.6% (7)
Pelvic urothelial carcinoma (n = 26)	KD2001	34.6% (9)	30.8% (8)	11.5% (3)	23.1% (6)
Carcinoid (n = 21)	NE841, LV1221	0.0% (0)	47.6% (10)	28.6% (6)	23.8% (5)
Hepatocellular carcinoma stages I-IIIa (n = 136)	LV1221a, BC000120b, LV1221	4.4% (6)	59.5% (81)	10.3% (14)	25.7% (35)
Lymph node diffuse large B cell lymphoma (DLBL) (n = 30)	LY301	23.3% (7)	33.3% (10)	13.3% (4)	30.0% (9)
Chondrosarcoma (n = 20)	BO2081	0.0% (0)	55.0% (11)	5.0% (1)	40.0% (8)
Hepatocellular carcinoma stage IV (n = 28)	LV1221	9.7% (3)	38.7% (11)	6.4% (1)	45.2% (13)
Endometroid adenocarcinoma (n = 38)	BC000120b	0.0% (0)	42.1% (16)	7.9% (3)	50.0% (19)
Intrahepatic cholangiocarcinoma (n = 40)	LV1221	25.0% (10)	17.5% (7)	5.0% (2)	52.5% (21)
Ovarian adenocarcinoma (n = 38)	BC000120b	5.3% (2)	36.8% (14)	2.6% (1)	55.3% (21)
Renal clear cell carcinoma (n = 138)	KD2001	5.1% (7)	26.8% (37)	6.5% (9)	61.6% (85)
Gastric adenocarcinoma (n = 37)	BC000120b	8.1% (3)	13.5% (5)	0.0% (0)	78.4% (29)
Head and neck squamous cell carcinoma (n = 38)	BC000120b	2.6% (1)	18.4% (7)	0.0% (0)	78.9% (30)

Numbers indicate the number of cores found utilitarianly positive for the respective receptor expression (IRS ≥ 6). The co-expression has been determined when both receptors on the same core were positive (IRS ≥ 6).

**Table 3:** Expression of SST2A and SST3 in screening group, represented by less than 20 cores.

Diagnosis	TMA ID	SST2A^+^/3^–^ % (n)	SST3^+^/2A^–^ % (n)	SST2A^+/^3^+^ % (n)	SST2A^–^/3^–^ % (n)
Plasma cell myeloma (n = 18)	BO2081	11.1% (2)	5.6% (1)	50.0% (9)	33.3% (6)
Atypical carcinoid (n = 18)	NE841	0.0% (0)	33.3% (6)	0.0% (0)	66.7% (12)
Renal carcinomas (n = 16)	KD2001	0.0% (0)	31.3% (5)	31.3% (5)	37.5% (6)
Neuroendocrine adenocarcinoma (n = 15)	NE841	0.0% (0)	20.0% (3)	20.0% (3)	60.0% (9)
Adamantioma (n = 12)	BO2081	0.0% (0)	50.0% (6)	0.0% (0)	50.0% (6)
Aneurismal bone cyst (n = 12)	BO2081	0.0% (0)	83.3% (10)	16.7% (2)	0.0% (0)
Adrenal cortical adenocarcinoma (n = 9)	AG801	0% (0)	33.3% (3)	1.1% (1)	55.5% (5)
Benign osteochondroma (n = 6)	BO2081	0.0% (0)	66.7% (4)	0.0% (0)	33.3% (2)
Metastatic cholangiocarcinoma (n = 4)	LV1221	100% (4)	0.0% (0)	0% (0)	0.0% (0)
Osteofibrous dysplasia (n = 5)	BO2081	0.0% (0)	100.0% (5)	0.0% (0)	0% (0)
Breast invasive carcinoma (n = 4)	TP242f	0.0% (0)	50.0% (2)	25.0% (1)	25.0% (1)
Lung carcinomas (n = 4)	TP242f	25.0% (1)	75.0% (3)	0.0% (0)	0.0% (0)
Prostate adenocarcinoma (n = 4)	TP242f	0.0% (0)	25.0% (1)	0.0% (0)	75.0% (3)
Colon adenocarcinoma (n = 4)	TP242f	50.0% (2)	25.0% (1)	0.0% (0)	25.0% (1)
Lung small cell carcinoma (n = 4)	NE841	0.0% (0)	75.0% (3)	0.0% (0)	25.0% (1)
Mixed HCC (n = 4)	LV1221	0.0% (0)	50.0% (2)	0.0% (0)	50.0% (2)
Chondroma (n = 4)	BO2081	75.0% (3)	0.0% (0)	0.0% (0)	25.0% (1)
Chordoma (malignant) (n = 4)	BO2081	0.0% (0)	0.0% (0)	0.0% (0)	100.0% (4)
Ewing’s sarcoma (n = 4)	BO2081	25.0% (1)	25.0% (1)	25.0% (1)	25.0% (1)
Bone squamous cell carcinoma (n = 4)	BO2081	0.0% (0)	75.0% (3)	0.0% (0)	25.0% (1)
Mediastinum squamous cell carcinoma (n = 2)	NE841	100.0% (2)	0.0% (0)	0.0% (0)	0.0% (0)
Adenosquamous carcinoma of liver (n = 2)	LV1221	0.0% (0)	0.0% (0)	0.0% (0)	100.0% (2)
Chondroblastoma (n = 2)	BO2081	0.0% (0)	100.0% (2)	0.0% (0)	0.0% (0)
Osteoblastoma (malignant) (n = 2)	BO2081	0.0% (0)	0.0% (0)	0.0% (0)	100.0% (2)

Numbers indicate the number of cores found utilitarianly positive for the respective receptor expression (IRS ≥ 6). The co-expression has been determined when both receptors on the same core were positive (IRS ≥ 6).

In the present manuscript, we interpreted our results both with the modern conventional IRS cutoff ≥ 3 and with a clinically relevant IRS ≥ 6 (Fig. 1). The data indicate that the modern (revised) cutoff might have overestimated the expression of both receptors in the tested cohort. This is in line with Yu et al. results and other published data. Therefore, using the clinically relevant cutoff ≥ 6 was justified by the data and was adopted for further analysis.

Of the entire data set, ca. two-thirds (743 cores, 66%) show clinically relevant expression of at least one of the investigated receptors. Seventy-five cores (6.7%) have been found positive only for SST2A, and 431 cores (38.3%) were found positive only for SST3. About one-fifth of the cores (237, 21%) have demonstrated co-expression of SST2A and SST3. The rest of the cores, about one-third (382, 34%), did not express either receptor at a clinically significant level.

In the current study, 43 clinical conditions were included. Of these conditions, 18 were each represented by more than 20 cores (extensive group, Table 2), and 25 were represented by less than 20 cores (screening group, Table 3). Each of the extensive group indications demonstrated significant expression of at least one receptor subtype. Of all the indications, three conditions (adenosquamous liver carcinoma, malignant chordoma, and malignant osteoblastoma) did not show clinically significant expression of any of the investigated receptors. Nevertheless, the low number of tested cores in these indications (derived from an even smaller number of patients due to duplicate cores) may not necessarily represent the actual situation accurately.

In the extensive group, 5 of 18 indications demonstrated over 30% co-expression of the tested receptor subtypes. Yet, more than half of all the tested indications (26 of 43) exhibited less than 10% of cores co-expressing the SST2A and SST3 at a clinically relevant magnitude. This finding may however be biased by the indications represented by six cores or fewer.

The indications can be roughly classified into abundantly-expressing groups (showing at least 50% of cores expressing at least one receptor subtype) and poorly-expressing indications (over 50% of double-negative cores). The share of abundantly-expressing indications was 13 out of 18 in the extensive group and 17 out of 25 in the screening group, represented by 780 cores out of 1125 total (69.3%). Among the abundantly-expressing indications, the expression distribution of the cores per receptor subtype was 53 (6.8%) for SST2A^+^/3^–^, 345 (44.2%) for SST3^+^/2A^–^, and 220 (28.2%) for SST2A^+^/3^+^, which is in overall good correspondence to the total results.

**Figure 2:**
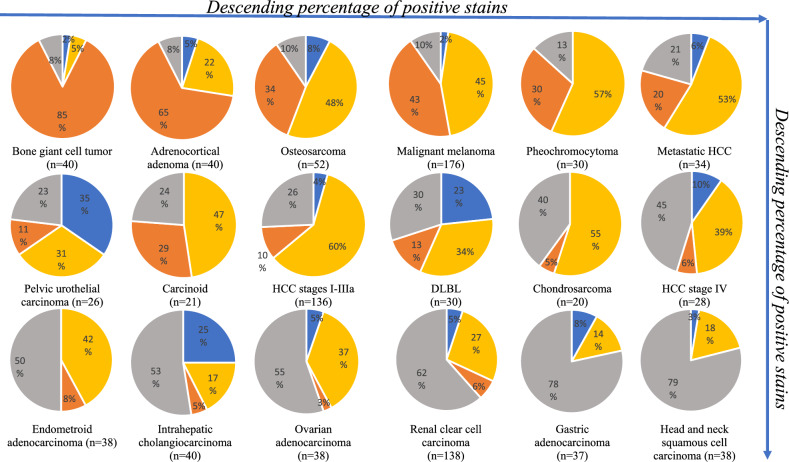
Prevalence of SST2A and SST3 expression in selected indications, by IRS ≥ 6, ordered from most expressing (top left) to least expressing (bottom right): Blue—% samples expressing SST2A only; Yellow—% samples expressing SST3 only; Orange—% samples expressing both SST2A and SST3; Grey—% samples not expressing SST2A or SST3.

Table 2 summarizes the data of clinical conditions represented by more than 20 cores, and organized in ascending order of the tissues that are not expressing SST2A nor SST3 at a clinically relevant extent. Table 3 summarizes medical conditions represented by fewer cases, and is organized in descending order according to the number of tissue cores in each diagnosis. Figure 2 demonstrates the data of Table 2 as a set of receptors’ expression distribution in juxtaposed pie charts. The data on the cores, including available anamnesis, tissue identification, and the individual scoring is provided in the Table SI-3.

The data were analyzed qualitatively and quantitatively for correlation between various patient- and disease-defined factors. A negative correlation trend was found in some tumors between tumor stage and SST2A and SST3 expression rate. Specifically, 198 cases of hepatocellular carcinoma (HCC), were included in the present study: most of them (136) were at stages I-III, 28 were defined as stage IV, and 34 cores represented HCC metastases from liver that were excised from different organs. Stages I-III group showed a higher prevalence of SST receptors expression compared to stage IV group. Among the early-stage cases approximately one quarter (26%) were negative for both receptors, and three-quarters (74%) expressed at least one of the two investigated receptors. Among the advanced tumors (stage IV), almost half (45%) were negative for both receptors. Interestingly, the metastases exhibited abundant expression, similar to the early stages. The pie-charts demonstrating the data are found in the upper right column and in the middle row of Fig. 2.

**Figure 3:**
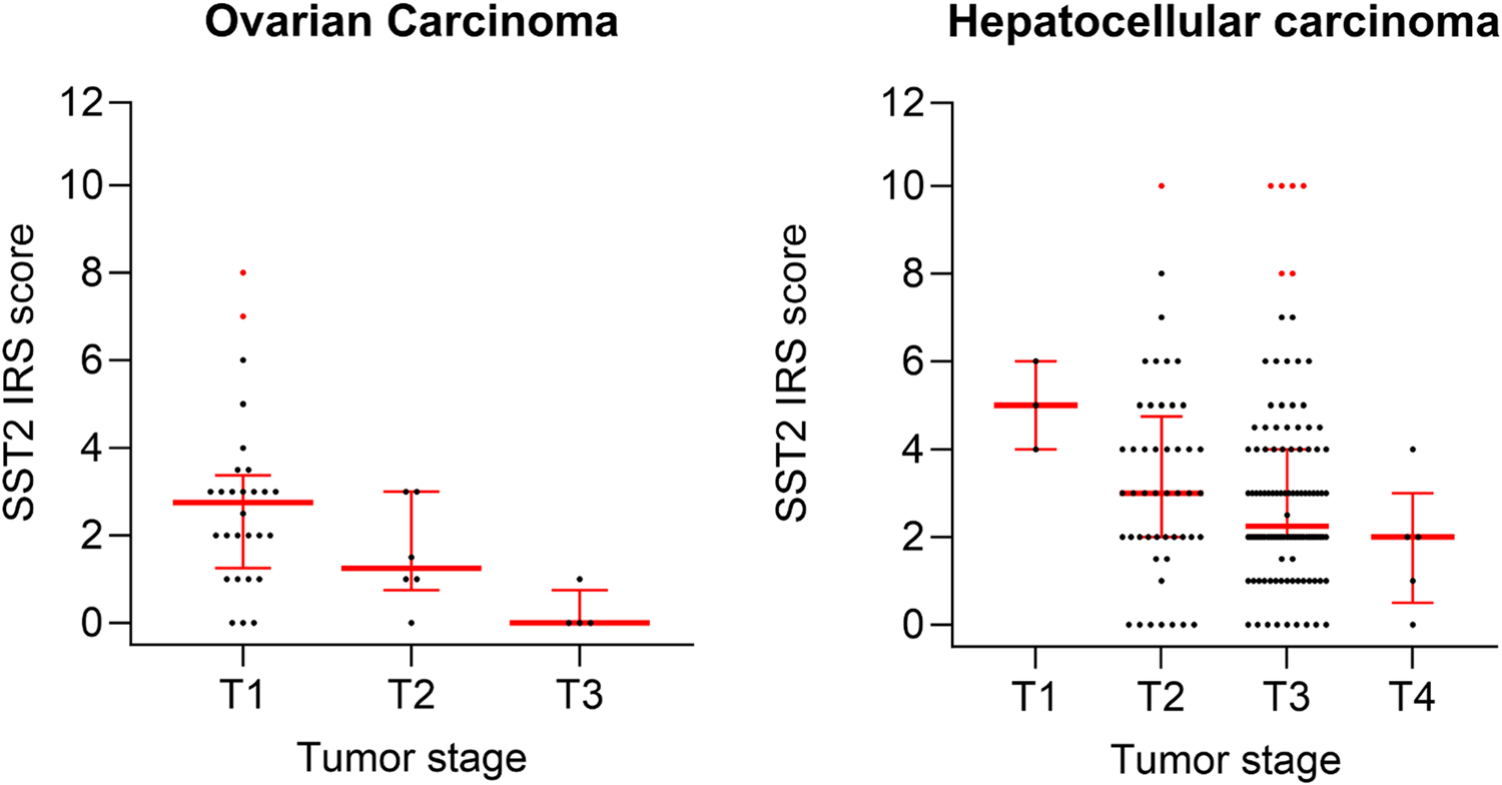
Relationship between SST2A expression intensity and tumor T (TNM) in ovarian carcinomas (left) and hepatocellular carcinomas (right). SST2 IRS individual values distribution of 38 ovarian carcinoma tissues (T1: n = 28, T2: n = 6, T3: n = 4) and 167 HCC tissues (T1: n = 3, T2: n = 49; T3: n = 110; T4 = 5) are presented as black dots. Upper and lower quartiles, median and outlier values (> quartile 3 + 1.5 × interquartile range) are depicted in red. By GraphPad Prism 10.0.3 (275).

In the cores for which the TNM and grading data were available, a statistically significant negative correlation with the T status (tumor size) and also with the tumor stage was found for the SST2 in the hepatocellular carcinomas and particularly in the ovarian carcinomas (Fig. 3, with Kendall τ and Kruskal Wallis values of 0.037/0.089, and 0.004/0.013, respectively). This supports the hypothesis that the tumors may lose the receptor as the disease progresses. Same trend was observed also for SST3 expression but failed to reach the statistical significance.

Significant tendency correlations between SST2 and SST3 expression were frequently observed (e.g. adrenocortical tumors (Spearman rho = 0.508, p < 0.001), ovarian carcinomas (rho = 0.297, p = 0.037), endometrial carcinomas (rho = 0.583, p < 0.001), renal clear cell carcinomas (rho = 0.355, p = 0.002), and melanomas (rho = 0.363, p < 0.001)).

**Figure 4:**
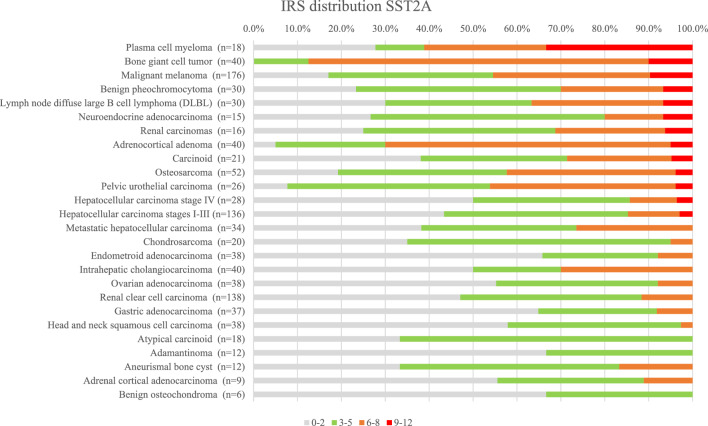
IRS of SST2A expression in the tested specimens, as percentile of the tested core numbers. Indications represented by more than 6 cores are shown.

**Figure 5:**
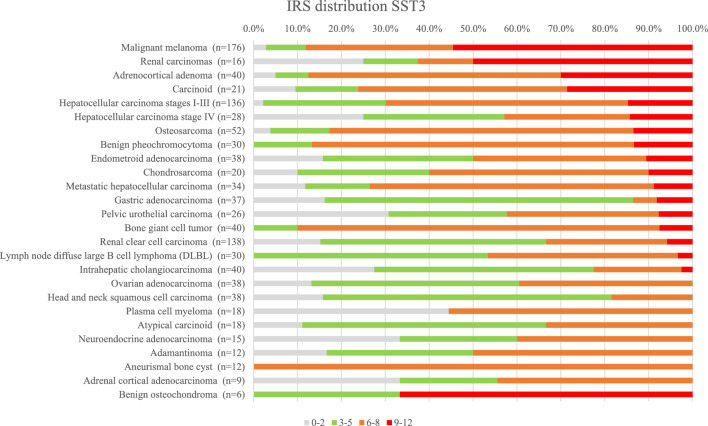
IRS of SST3 expression in the tested specimens, as percentile of the tested core numbers. Indications represented by more than 6 cores are shown.

The IRS values distribution in the tested samples is shown in Figs. 4 and 5. The values were charted as normalized stacked bar charts, with the percentile of the correspondingly stained cores per indication being charted per indication and categorized by color code, for SST2A and SST3, respectively. The cores representing indications originating from several TMAs, were pooled together. Only indications represented by more than 6 cores are shown.

## Discussion

In the current study, a longitudinal screening of 1125 cores from 964 cases were assessed by IHC for the presence of somatostatin GPCR subtypes 2A and 3 in a wide range of human cancer tissues. To the best of our knowledge, the data presented in the current manuscript is the first and the largest collection of a single-protocol – single-evaluator IHC results for comparative expression levels of somatostatin receptors type 2A and 3 in paired samples.

The results of this study show that two-thirds of the tested specimen cores exhibit clinically significant expression of the SST2 and/or SST3, and at least 21% of the specimens were clinically positive for both. Furthermore, the data of this study intriguingly indicates a tendency for reduced expression of SST-GPCRs with tumor progression and the unexpected significant recurrence of overexpression of both receptor subtypes in many tested metastases samples.

A further feature revealed by the study is the relatively high prevalence of SST3 expression in tumors. Emphasizing that the observation of this relatively high prevalence of SST3 was supported by the clinically relevant high IRS ≥ 6 analysis. The main reason for this intriguing finding appears to be our choice of the clinically relevant cut-off value of IRS ≥ 6 and not the previously standard cut-off value ≥ 2 or ≥ 3, also for SST2A, which was interpreted as mild and regardless of clinically relevant expression, as positive^32–34^. Notwithstanding, it is undeniable that the data strongly suggests that SST3 should be an accepted subject of medical interest, which might have been overlooked for an unduly long time.

The findings of positive expression of SST2 and/or SST3 in various solid tumors observed in our study are in concordance with previously published data, indicating that various human cancerous tissues exhibit SST expression, including GEP-NET, lymphoma, gastrointestinal carcinoma, head and neck carcinoma, lung carcinoma, Merkel cell carcinoma, malignant melanoma, meningioma, neuroblastoma, thyroid carcinoma, thymoma, breast carcinoma, pheochromocytoma, paraganglioma, prostate carcinoma, ovarian carcinoma, and endometrial carcinoma, as recently reviewed by Priyadarshini et al.^11^. Remarkably, some tumor types have been reported to express SST3 at similar levels or higher than SST2A (some GEP-NETs, thyroid carcinoma, thymoma, breast cancer, ovarian tumors, pheochromocytomas, and specially nonfunctioning pituitary tumors)^51–60^. Moreover, abundant co-expression of SST2A and SST3 in certain cancers was also reported^61^. Notably, in normal tissues, SST3 expression is limited to the brain, testis, and duodenum^62^ &@ https://www.proteinatlas.org/ENSG00000278195-SSTR3/tissue, last accessed on 15th of March, 2023, whereas SST2A is much more ubiquitously present in the body (*ibid*, &@ https://www.proteinatlas.org/ENSG00000180616-SSTR2/tissue).

In the current work, we aimed to evaluate both the individual and co-expression of SST2A and SST3 in human cancer tissues. This type of information is indispensable to enable selecting the most suitable somatostatin analog to target and treat a specific tumor type. For example, our data suggests that metastatic intrahepatic cholangiocarcinoma and pelvic urothelial carcinoma mainly express SST2 (Fig. 2). Therefore, patients bearing these types of cancer may more likely benefit from the therapy based on DOTA-TATE ligand, which selectively and specifically targets SST2. On the other hand, octreotide, pasireotide, and DOTA-NOC that demonstrate broader SST subtypes affinities, including both SST2 and SST3^63^, should be considered as diagnostic and therapeutic option for tumors showing expression of receptor subtypes other than exclusively SST2. For example, they may be better used for non-functioning pituitary adenoma^50^, hepatocellular carcinoma^64^ (also see Fig. 2), pheochromocytoma^65^ (also see Fig. 2), melanoma^10^ (also Fig. 2) and others. A newly emerged, not yet clinically approved SST3-specific analog^66^ and a pan-somatostatin analog^49^ may potentially also be useful in these cases. The results of the current study highlight SST3 as a potential powerful candidate biomarker alongside the clinically validated SST2A. Once selective SST3 ligands are clinically available, they might provide a new therapeutic option for patients who are SST3 positive. Our findings suggest that this group may include malignant melanoma, hepatocellular carcinoma, osteosarcoma, pheochromocytoma, and several others.

Another intriguing finding of this study is the dynamic expression of SST2 and SST3 that tend to decline alongside tumor progression at least in certain tumors. This phenomenon is well known in neuroendocrine tumors, presenting decreased SST2A expression with increasing tumor grading and staging. The results of the current study concur with this tendency, especially in the 38 samples of ovarian adenocarcinoma and 198 samples representing different stages of HCC. Interestingly, metastases tend to re-express both SST2A and SST3 at levels that are similar or exceeding the earliest stages, indicating that these cells conserved their genotypic potential of protein synthesis during the tumor evolution, despite the observed phenotypic shift. This may also indicate a mutually affecting interrelationship between the tumor and its microenvironment. Additionally, since somatostatin receptors 2A and 3 are implicated in apoptosis, anti-angiogenesis, and cell cycle arrest, their overexpression at earlier stages may be, at the molecular level, a proliferation inhibiting attempt, as a defense tumor suppression response of the body against tumorigenesis.

As to the particular choice of the target GPCRs in the present work, structurally, SST3 exhibits a long carboxyl-terminal tail, making it the largest subtype with 418 amino acids. SST2A and SST5 are the smallest, with only 369 and 363 amino acids, respectively. While SST subtypes 1, 2, 4, and 5 are known to induce cell cycle arrest, apoptosis is induced by SST2 and mainly by SST3^9^, and anti-angiogenesis effect, by inhibition of both eNOS and MAPK activities, is exclusively contributed by SST3^67^. Pharmacologically, upon agonist binding SST3 is rapidly and effectively internalized by the recruitment of β-arrestin^68–70^. In fact, SST3 is the most efficient subtype in ligand internalization. The receptor-mediated internalization capability of SST3 was found to be almost four times higher than SST2A (78% vs. 20%)^71^.

Moreover, it ought to be borne in mind that SST2A and SST3 take part in an extensive and complex functional crosstalk when the two subtypes appear concomitantly on the same cell^72^. When co-expressed on the cell membrane, SST2A and SST3 tend to form both constitutive homodimeric and heterodimeric complexes. However, their GPCR-ligand complexes demonstrate distinct trafficking upon exposure to their specific agonists: SST2A-specific agonist induces the dissociation of SST2A homodimers into monomers followed by internalization, whereas SST3 internalizes as a homodimer^73,74^. Internalization of GPCR heterodimers is induced both by SST2A- and SST3-specific ligands^72^, whereas binding of somatostatin-14 inactivates heterodimeric SST3 and only SST2A undergoes internalization^75^. This interplay between intrafamily GPCR subtypes suggests that several tumor types that co-express these receptor subtypes, as revealed in our study, might be effectively targeted by both SST2- or SST3-based targeted therapies.

The evidence of low expression of SST3 in normal tissues, together with its relatively high internalization efficiency among the other SST subtypes^71^ and high expression level of this receptor subtype in tumors, strongly supports its potential as therapeutic target in cancer. Significant co-expression of SST3 with SST2A in some indications may also provide the rationale to improve the targeting of approved SST2A agonists with further SST3-targeting agents.

Immunohistochemistry has been considered for decades the “gold standard” methodology for *in-situ* protein expression analysis in tissue samples. Moreover, target validation by IHC has been designated as the first recommended clinical diagnostic step for the new therapeutic modalities seeking targeted cancer therapy by the Cancer Therapy Evaluation Program of the US National Cancer Institute^76^. Combining IHC and tissue microarray technology allows an effective simultaneous analysis of hundreds of tissue samples while keeping maximal experimental standardization^77–79^, and was therefore selected for the present study. However, the reliability of IHC results depends on many critical elements throughout the assay. A calibrated, preferably validated IHC protocol should be used with adequate controls whenever possible. The sources of variability may come from each step of the assay. For example, the physicochemical processes of sample preparation, involving tissue fixation, embedding, slicing, and mounting may damage the tissue and alter the natural and authentic target expression. Conditions used for antigen retrieval and even interlaboratory inconsistencies, may also significantly affect the results. Moreover, the selection of an appropriate target-specific primary antibody is a crucial feature in the study design and implementation of IHC. Notably, unoptimized use of antibodies in terms of selectivity and concentration may cause both false positive and false negative results^77^. For many molecular targets, including SSTs, highly selective and specific antibodies and a corresponding validated protocol are not yet available. Being aware of this critical choice, prior to performing the immunostaining of the TMAs, nine different commercially available SST3 antibodies produced by different manufacturers were evaluated, and the most specific antibody was selected to this study (Table SI1, Figs. SI 1–3). Finally, one prominent key feature of IHC is the scoring of the stained slides to evaluate the expression of the target molecules. Although artificial-intelligence-based scoring approaches are emerging, the vast majority of clinical IHC diagnostic data are still being scored manually by experienced histopathologists in the relevant clinical settings. Yet, using digital image analysis software, such as QuPath™, is becoming increasingly popular recently. Automated digital image analysis (DIA) has the potential to provide eventually the objectivity, reliability, and analysis speed, required to radically transform tissue biomarker research, discovery, and routine testing. However, achieving reliable results using automatic scoring requires an intensive model training for each individual biomarker in a variety of indications, and validation compared to manual scoring. This requires significant effort and expertise, and caution should usually be exercised that the utilized model has been trained on an appropriate set and duly validated. Since the aim of the present study was to evaluate the clinically relevant expression of SST2A and SST3, clinically acceptable manual evaluation was used.

The use of several alternative semi-quantitative scoring methods (e.g. Her2, IRS, H-score) in different studies significantly contributes to the complexity of the data analysis, interpretation, and comparison. In the present study we attempted to minimize these variabilities via a unified protocol and streamlined evaluation, attaining the comparative power of the results from the large number of paired evaluated cores.

Practically, the experimental approaches employed in this study aimed to maximize reliability and reproducibility of results. The use of consecutively prepared tissue microarray slides ensured that the specimens were derived from the same source and could be pairwise compared and evaluated. One evident limitation of the present study is the unequal number of tested cores per tumor type. This was particularly important for the diseases that were represented in a low number of cores. This drawback stems from the usage of commercially available rather than custom-made TMAs. Moreover, the analysis of SST2A expression, valuable per se, is expected to facilitate comparison of, and to, the previously published data, on both receptors. Nevertheless, the data from the limited number of observations must be treated with caution and regarded only as indicative.

The overall quality of observed data of this study was deemed satisfactory. The repetitive cores generally furnished identical or very similar results, as would be expected when the slide preparation methodology is streamlined in TMAs, and the evaluation is performed according to identical criteria and by the same evaluator. The different sets of cores originating from several different TMAs also furnished comparable results, particularly regarding co-expressions of the two receptors on a single tissue. The power of the study lies in the high overall number of tested specimens, its broad design, including many types of cancer, the clinically oriented interpretation, and the internal integrity of the data.

Noteworthy, despite our best effort to find the most suitable SST3 antibody for this wide survey, certain non-specific staining was still observed in some of the cores. For this reason, the results reported herein might involve a slight overestimation. Nevertheless, the extent of the problem has not been considered objectionable. Foregoing notwithstanding, the large number of tested cores empowers sufficient confidence at least in the general trend which was our focus. Moreover, positive staining of blood vessels, endothelial, and stroma cells, all belonging to the cancerous tissue may indicate potential targeting anchors not limited to the tumor cells, but also in the tumor’s microenvironment. To this end, scientific reports support the overexpression of SST3 in angiogenic vasculature around the tumor^67^, therefore this should not necessarily indicate non-specific staining when referring to a tumor tissue. Once a better antibody be available, reassessment of expression will be of value, as has been previously done^36,38,39^.

Targeted therapy has long been recognized as an effective strategy to decrease the general toxicity of conventional cancer chemo- and radiotherapy, increasing the tolerability, maximizing the efficacy of selected treatment, and thus improving survival rate. The future of personalized medicine depends on further biomarkers discovery and the development of appropriate ligands^80^. Our present findings indicate that SSTs are yet to reach their full therapeutic potential, despite extensive SST2A-agonist clinical trials. Targeting SST2A^+^/3^+^ co-expressing cancers or SST3^+^ cancers may bring an additional treatment opportunity to numerous patients currently lacking options. A thorough understanding of SSTs expression patterns in various tumor types might reveal additional receptor subtypes as potential cancer biomarkers, leading eventually to the development of further diagnostic and therapeutic tools to expanding the patient population that can benefit from a personalized therapy approach.

## Materials and methods

### Tumor specimens

Two consecutive copies of ten human paraffin-embedded tissue microarrays were purchased from US Biomax, Inc (USA). According to the manufacturer declaration, each tissue has been collected under the highest ethical standards with the donor being informed completely and with their consent. The standard medical care was followed, and the donors’ privacy is being protected. All human tissues have been collected under HIPPA approved protocols, have been tested negative for HIV and Hepatitis B or their counterparts, and approved for commercial product development. The identification and description of tissue microarrays are summarized in Table 1. Comparative assessments of SST2 and SST3 were performed on paired slides by utilizing the receptor-specific antibodies for parallel immunostainings. The slides contained several duplicate cores, but mainly single cores from a variety of patients. A total of 1125 cores from 964 cases were screened. Seven cores were disqualified from the analysis due to technical reasons: one from each of the NET adenocarcinoma, adrenal cortical adenocarcinoma, intrahepatic cholangiocarcinoma, gastric adenocarcinoma, and osteofibrous dysplasia groups, and two from the benign osteochondroma group.

### Antibodies selection

An antibody screening was performed by testing nine different anti-SST3 antibodies. The list of tested antibodies is presented in Table SI-1.

Rabbit monoclonal antibody clone UMB-5 (Abcam Cambridge, UK, Cat. # ab137026) showed highest specificity and selectivity along with a favorable signal to background ratio. Therefore, it was selected for SST3. Figure SI-1 demonstrates positive and negative staining of human pancreatic tissue using the selected antibody and a representative non-selected antibody. For consistency, the rabbit monoclonal anti-human somatostatin receptor 2 clone UMB-1 was used for SST2 determination (Cat. # ab134152).

### Calibration

Each primary antibody (including ones that were not selected) was calibrated in order to determine the optimal concentration prior to TMAs staining. Normal human pancreatic islets served as positive controls. As negative control, the primary antibody was omitted. In both antibodies a complete abolishment of immunostaining was obtained. Figure SI-2 demonstrates pancreatic stain using the chosen optimal dilution of SST2 antibody. Figure SI-3 shows staining of human pheochromocytoma tissue sample with optimal and sub-optimal dilution of SST3 antibody.

### Immunohistochemistry

Immunostaining was performed using an indirect peroxidase labelling method. Briefly, sections were dewaxed and rehydrated via a graded ethanol series, during which endogenous peroxidases were blocked by an additional incubation of the slides in 0.3% hydrogen peroxide in methanol for 45 min. Samples were then microwaved in 10 mM citric acid (pH 6.0) for 16 min at 600 W and incubated either with the anti-SST2 (dilution: 1:250) or the anti-SSTR3 antibody (dilution: 1:1000) overnight at 4 °C, followed by incubation with biotinylated goat anti-rabbit IgG and peroxidase-conjugated avidin (Vector ABC “Elite” kit; Vector Laboratories, Burlingame, CA, USA). Non-specific binding was blocked using normal goat serum. The binding of the primary antibody was visualized using 3-amino-9-ethylcarbazole in acetate buffer (BioGenex, San Ramon, CA, USA). Sections were counterstained with Mayer’s hematoxylin and mounted in Vectamount™ mounting medium (Vector Laboratories, Burlingame, CA, USA).

### Scoring

Expression of SST2 and SST3 was evaluated. The evaluation of the stained sections was performed manually using light microscopy, by an expert senior scientist. All scoring was performed by the same investigator to ensure uniformity. Samples were viewed at ample magnification using a light microscope. The entire core containing the tissue sample of each analyzed tumor specimen was evaluated. The proportion of the stained cells was estimated by counting and averaging several representative regions to classify the specimen according to the semi-quantitative immunoreactive score (IRS) suggested by Remmele and Stegner^32^. Ranking was applied to the fraction of stained cells, as 0: no positive cells; 1: < 10%; 2: 10–50%; 3: 51–80%; 4: > 80% positive cells. Simultaneously, ranking of the staining intensity was applied, the intensity being estimated ranked as 0: no staining; 1: weak staining; 2: moderate staining; 3: strong staining. Then, the IRS was calculated by multiplying staining intensity rank with the ranking of percentage of positively stained cells. The overall IRS ranges were therefore between 0 and 12. As elaborated extensively above, the threshold of “positive” expression has been set to 6, since this threshold appears to be more clinically relevant. The scoring calculation and evaluation criteria are also summarized in Table SI-2 and Fig. 1.

### Statistical analysis

Correlation analysis was performed using *SPSS* software (version 29.0.0, IBM SPSS Inc., Armonk, NY, USA). Non-parametric Spearman test was performed to evaluate the correlation between the T status of the cores and their respective expression of SST2A and SST3. Kendall tau test was used to determine co-expression correlation, corroborated by Kruskal–Wallis non-parametric multivariate analysis. The significance was accepted with the probability of committing a type I error (alpha) being 5% and less (α = 0.05).

### Supplementary Information

Supplementary Information.

## Data Availability

All data analyzed during the study are included in this published article (and its Supplementary Information files).

## References

[1] Yan L, Rosen N, Arteaga C (2011). Targeted cancer therapies. Chin. J. Cancer.

[2] Scholl S, Beuzeboc P, Pouillart P (2001). Targeting HER2 in other tumor types. Ann. Oncol..

[3] Schramm A (2015). Targeted therapies in HER2-positive breast cancer-a systematic review. Breast Care.

[4] Chaudhary PK, Kim S (2021). An insight into GPCR and G-proteins as cancer drivers. Cells.

[5] Hauser AS (2018). Pharmacogenomics of GPCR drug targets. Cell.

[6] O’Hayre M, Degese MS, Gutkind JS (2014). Novel insights into G protein and G protein-coupled receptor signaling in cancer. Curr. Opin. Cell Biol..

[7] Lamberts SW (1996). Octreotide. N. Engl. J. Med..

[8] Patel Y (1993). Multiple gene transcripts of the somatostatin receptor SSTR2: Tissue-selective distribution and cAMP regulation. Biochem. Biophys. Res. Commun..

[9] Grozinsky-Glasberg S (2008). Somatostatin analogues in the control of neuroendocrine tumours: Efficacy and mechanisms. Endocr. Relat. Cancer.

[10] Lum SS (2001). Distribution and functional significance of somatostatin receptors in malignant melanoma. World J. Surg..

[11] Priyadarshini S, Allison DB, Chauhan A (2022). Comprehensive assessment of somatostatin receptors in various neoplasms: A systematic review. Pharmaceutics.

[12] Reubi J (2001). Somatostatin receptor sst1–sst5 expression in normal and neoplastic human tissues using receptor autoradiography with subtype-selective ligands. Eur. J. Nucl. Med..

[13] Sun L, Coy DH (2016). Somatostatin and its analogs. Curr. Drug Targets.

[14] Afargan M (2001). Novel long-acting somatostatin analog with endocrine selectivity: Potent suppression of growth hormone but not of insulin. Endocrinology.

[15] Strosberg J, Leeuwenkamp O, Siddiqui MK (2021). Peptide receptor radiotherapy re-treatment in patients with progressive neuroendocrine tumors: A systematic review and meta-analysis. Cancer Treat. Rev..

[16] FDA, *FDA approves new diagnostic imaging agent to detect rare neuroendocrine tumors.* https://www.fda.gov/news-events/press-announcements/fda-approves-new-diagnostic-imaging-agent-detect-rare-neuroendocrine-tumors (2016).

[17] FDA, *FDA approves new treatment for certain digestive tract cancers.* https://www.fda.gov/news-events/press-announcements/fda-approves-new-treatment-certain-digestive-tract-cancers (2018).

[18] ClinicalTrials.Gov. *A Dose Finding Study of [177Lu]Lu-DOTA-TATE in Newly Diagnosed Glioblastoma in Combination With Standard of Care and in Recurrent Glioblastoma as a Single Agent*. https://clinicaltrials.gov/ct2/show/NCT05109728 (2021).

[19] ClinicalTrials.Gov. *A Safety Study of [177Lu]Lu-DOTA-TATE in Newly Diagnosed Extensive Stage Small Cell Lung Cancer (ES-SCLC) Patients in Combination With Carboplatin, Etoposide and Tislelizumab*. https://clinicaltrials.gov/ct2/show/NCT05142696 (2021).

[20] ClinicalTrials.Gov. *177Lu-DOTATATE for the Treatment of Stage IV or Recurrent Breast Cancer*. https://clinicaltrials.gov/ct2/show/NCT04529044 (2020).

[21] ClinicalTrials.Gov. *Phase II 177Lu-DOTATATE Study in Metastatic NPC With a Safety Run-in (SG-AAA-II-01)*. https://clinicaltrials.gov/ct2/show/NCT05198479 (2022).

[22] ClinicalTrials.Gov. *177Lutetium-DOTATATE in Children with Primary Refractory or Relapsed High-risk Neuroblastoma (LuDO-N)*. https://clinicaltrials.gov/ct2/show/NCT04903899 (2021). 10.3389/fped.2022.836230 PMC8960300 35359899

[23] Chauhan A (2023). SSTR-2 expression in solid tumors: An immunohistochemistry analysis. Endocr. Abstr. Biosci..

[24] ClinicalTrials.Gov. *Lutathera for the Treatment of Inoperable, Progressive Meningioma After External Beam Radiation Therapy*. https://clinicaltrials.gov/ct2/show/NCT04082520 (2019).

[25] Redko B (2015). Synthesis, drug release, and biological evaluation of new anticancer drug–bioconjugates containing somatostatin backbone cyclic analog as a targeting moiety. Peptide Sci..

[26] Chatzellis E, Kaltsas G, Chatzellis E, Kaltsas G (2019). Somatostatin receptor expression in gastrointestinal tumors. Encyclopedia of Endocrine Diseases.

[27] Harda K (2018). Somatostatin receptors as molecular targets in human Uveal melanoma. Molecules.

[28] Krenning E (1993). Somatostatin receptor scintigraphy with [111 In-DTPA-D-Phe 1]-and [123 I-Tyr 3]-octreotide: The Rotterdam experience with more than 1000 patients. Eur. J. Nucl. Med..

[29] Reubi J (1997). Distribution of somatostatin receptors in normal and neoplastic human tissues: Recent advances and potential relevance. Yale J. Biol. Med..

[30] Fedchenko N, Reifenrath J (2014). Different approaches for interpretation and reporting of immunohistochemistry analysis results in the bone tissue–a review. Diagn. Pathol..

[31] Fitzgibbons PL (2014). Template for reporting results of biomarker testing of specimens from patients with carcinoma of the breast. Arch. Pathol. Lab. Med..

[32] Remmele W, Stegner H (1987). Recommendation for uniform definition of an immunoreactive score (IRS) for immunohistochemical estrogen receptor detection (ER-ICA) in breast cancer tissue. Der Pathol..

[33] Beyer A-SL (2021). Immunohistochemical evaluation of adaptor protein FAM159B expression in normal and neoplastic human tissues. Int. J. Mol. Sci..

[34] Yu J (2022). Correlation and comparison of somatostatin receptor type 2 immunohistochemical scoring systems with 68Ga-DOTATATE positron emission tomography/computed tomography imaging in gastroenteropancreatic neuroendocrine neoplasms. Neuroendocrinology.

[35] Schmid HA (2012). Monoclonal antibodies against the human somatostatin receptor subtypes 1–5: Development and immunohistochemical application in neuroendocrine tumors. Neuroendocrinology.

[36] Lupp A (2012). Reassessment of sst<sub>3</sub> somatostatin receptor expression in human normal and neoplastic tissues using the novel rabbit monoclonal antibody UMB-5. Neuroendocrinology.

[37] Leu FP, Nandi M (2010). GPCR somatostatin receptor extracellular loop 2 is a key ectodomain for making subtype-selective antibodies with agonist-like activities in the pancreatic neuroendocrine tumor BON cell line. Pancreas.

[38] Fischer T (2008). Reassessment of sst2 somatostatin receptor expression in human normal and neoplastic tissues using the novel rabbit monoclonal antibody UMB-1. J. Clin. Endocrinol. Metab..

[39] Lupp A (2011). Reassessment of sst5 somatostatin receptor expression in normal and neoplastic human tissues using the novel rabbit monoclonal antibody UMB-4. Neuroendocrinology.

[40] Kaemmerer D (2017). Somatostatin and CXCR4 chemokine receptor expression in hepatocellular and cholangiocellular carcinomas: tumor capillaries as promising targets. BMC Cancer.

[41] Czajkowski M (2022). Comparative evaluation of somatostatin and CXCR4 receptor expression in different types of thyroid carcinoma using well-characterised monoclonal antibodies. BMC Cancer.

[42] Remes SM (2019). Immunohistochemical expression of somatostatin receptor subtypes in a panel of neuroendocrine neoplasias. J. Histochem. Cytochem..

[43] Vesterinen T (2019). Somatostatin receptor expression is associated with metastasis and patient outcome in pulmonary carcinoid tumors. J. Clin. Endocrinol. Metab..

[44] Lambertini C (2013). Evaluation of somatostatin receptor subtype expression in human neuroendocrine tumors using two sets of new monoclonal antibodies. Regul. Pept..

[45] Papotti M (2002). Expression of somatostatin receptor types 1–5 in 81 cases of gastrointestinal and pancreatic endocrine tumors. A correlative immunohistochemical and reverse-transcriptase polymerase chain reaction analysis. Virchows Arch..

[46] Körner M (2018). A critical evaluation of sst3 and sst5 immunohistochemistry in human pituitary adenomas. Neuroendocrinology.

[47] Gou R-S (2013). Somatostatin receptors 3, 4 and 5 play important roles in gallbladder cancer. Asian Pac. J. Cancer Prev..

[48] Carmona Matos DM (2019). Characterization of somatostatin receptors (SSTRs) expression and antiproliferative effect of somatostatin analogues in aggressive thyroid cancers. Surgery.

[49] Modena D (2022). Identification of a novel SSTR3 full agonist and its activity in non-functioning pituitary adenoma model. Eur. Congr. Endocrinol..

[50] Øystese KA (2017). Estrogen receptor α, a sex-dependent predictor of aggressiveness in nonfunctioning pituitary adenomas: SSTR and sex hormone receptor distribution in NFPA. J. Clin. Endocrinol. Metab..

[51] Herrera-Martínez AD (2018). Clinical and functional implication of the components of somatostatin system in gastroenteropancreatic neuroendocrine tumors. Endocrine.

[52] Elston MS (2015). Increased SSTR2A and SSTR3 expression in succinate dehydrogenase–deficient pheochromocytomas and paragangliomas. Hum. Pathol..

[53] Ibáñez-Costa A (2016). Octreotide and pasireotide (dis) similarly inhibit pituitary tumor cells in vitro. J. Endocrinol..

[54] Hofland LJ (2010). Pituitary tumours: The sst/D2 receptors as molecular targets. Mol. Cell. Endocrinol..

[55] Lee M (2015). SSTR3 is a putative target for the medical treatment of gonadotroph adenomas of the pituitary. Endocr. Relat. Cancer.

[56] Ain KB (1997). Somatostatin receptor subtype expression in human thyroid and thyroid carcinoma cell lines. J. Clin. Endocrinol. Metab..

[57] Palmieri G (1997). Successful treatment of a patient with a thymoma and pure red-cell aplasia with octreotide and prednisone. N. Engl. J. Med..

[58] Schulz S (2002). Immunohistochemical detection of somatostatin receptors in human ovarian tumors. Gynecol. Oncol..

[59] Kumar U (2005). Somatostatin receptors in primary human breast cancer: Quantitative analysis of mRNA for subtypes 1–5 and correlation with receptor protein expression and tumor pathology. Breast Cancer Res. Treat..

[60] Zou Y (2019). Expression and selective activation of somatostatin receptor subtypes induces cell cycle arrest in cancer cells. Oncol. Lett..

[61] Kaemmerer D (2012). Comparing of IRS and Her2 as immunohistochemical scoring schemes in gastroenteropancreatic neuroendocrine tumors. Int. J. Clin. Exp. Pathol..

[62] Uhlén M (2015). Tissue-based map of the human proteome. Science.

[63] Hu Y (2021). Role of somatostatin receptor in pancreatic neuroendocrine tumor development, diagnosis, and therapy. Front. Endocrinol..

[64] Liu H-L, Huo L, Wang L (2004). Octreotide inhibits proliferation and induces apoptosis of hepatocellular carcinoma cells. Acta Pharmacol. Sin..

[65] Leijon H (2019). Variable somatostatin receptor subtype expression in 151 primary pheochromocytomas and paragangliomas. Hum. Pathol..

[66] Vázquez-Borrego MC (2020). A somatostatin receptor subtype-3 (SST3) peptide agonist shows antitumor effects in experimental models of nonfunctioning pituitary tumorsrole of SST3 in nonfunctioning pituitary tumors. Clin. Cancer Res..

[67] Florio T (2003). Somatostatin inhibits tumor angiogenesis and growth via somatostatin receptor-3-mediated regulation of endothelial nitric oxide synthase and mitogen-activated protein kinase activities. Endocrinology.

[68] Tower-Gilchrist C (2014). Monitoring endosomal trafficking of the G protein-coupled receptor somatostatin receptor 3. Methods in Enzymology.

[69] Kreuzer O (2001). Agonist-mediated endocytosis of rat somatostatin receptor subtype 3 involves β-arrestin and clathrin coated vesicles. J. Neuroendocrinol..

[70] Hukovic N (1996). Agonist-dependent regulation of cloned human somatostatin receptor types 1–5 (hSSTR1-5): Subtype selective internalization or upregulation. Endocrinology.

[71] Cuevas-Ramos D, Fleseriu M (2014). Somatostatin receptor ligands and resistance to treatment in pituitary adenomas. J. Mol. Endocrinol..

[72] War SA, Kumar U (2012). Coexpression of human somatostatin receptor-2 (SSTR2) and SSTR3 modulates antiproliferative signaling and apoptosis. J. Mol. Signal..

[73] Grant M, Collier B, Kumar U (2004). Agonist-dependent dissociation of human somatostatin receptor 2 dimers: A role in receptor trafficking. J. Biol. Chem..

[74] War SA, Somvanshi RK, Kumar U (2011). Somatostatin receptor-3 mediated intracellular signaling and apoptosis is regulated by its cytoplasmic terminal. Biochim. Biophys. Acta.

[75] Pfeiffer M (2001). Homo- and heterodimerization of somatostatin receptor subtypes. Inactivation of sst(3) receptor function by heterodimerization with sst(2A). J. Biol. Chem..

[76] Kunos CA (2021). Radiopharmaceutical validation for clinical use. Front. Oncol..

[77] Simon R, Mirlacher M, Sauter G, Simon R (2010). Immunohistochemical analysis of tissue microarrays. Tissue Microarrays: Methods and Protocols.

[78] Eckel-Passow JE (2010). Tissue microarrays: One size does not fit all. Diagn. Pathol..

[79] Au-Zlobec I (2014). A next-generation tissue microarray (ngTMA) protocol for biomarker studies. JoVE.

[80] Vargas AJ, Harris CC (2016). Biomarker development in the precision medicine era: Lung cancer as a case study. Nat. Rev. Cancer.

