# Supplementary material for: Expression prevalence and dynamics of GPCR somatostatin receptors 2 and 3 as cancer biomarkers beyond NET: a paired immunohistochemistry approach
Source: Sci Rep. 2023 Nov 27;13:20857. doi: 10.1038/s41598-023-47877-0 (PMC10682014; doi:10.1038/s41598-023-47877-0)
Supplement: Supplementary file 1 — Supplementary Information. [file 41598_2023_47877_MOESM1_ESM.pdf]

Expression Prevalence and Dynamics of GPCR Somatostatin Receptors 2 and 3 as Cancer Biomarkers Beyond NET: a Paired Immunohistochemistry Approach

Mor Oron-Herman[1,*,+], David Kirmayer[1,+], Amelie Lupp[2], Stefan Schulz[2], Gennady Kostenich[1,3], Michel Afargan[1]

[1]Starget Pharma, 26 Snir st., Ramat Hasharon, 4704086, Israel
[2]Institute of Pharmacology and Toxicology, Jena University Hospital, Friedrich Schiller University Jena, Drackendorfer Str. 1, 07747 Jena, Germany
[3]The Advanced Technology Center, Sheba Medical Center, Tel Hashomer, 5262000, Israel
*corresponding author, mor@stargetpharma.com
[+]the authors contributed equally to the work

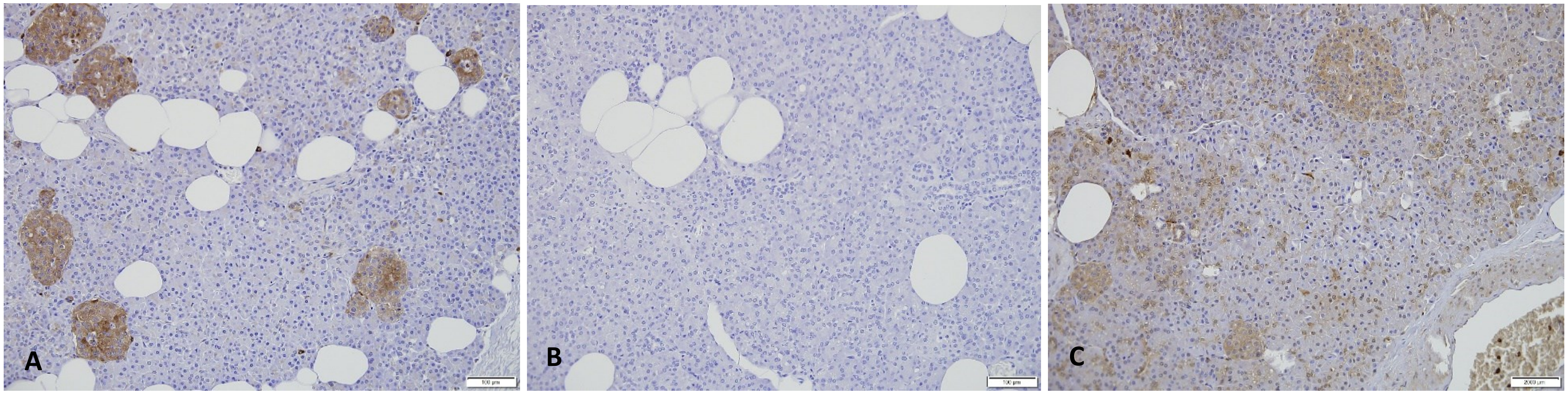

Figure SI-1: Representative staining of human pancreatic tissue using Rabbit monoclonal antibody anti human somatostatin receptor 3 clone UMB-5 (Ab137026). A: Positive Brown staining of pancreatic islets. B: Negative control. C: Staining with LS-2262. Staining is seen in pancreatic islets, and also in the surrounding tissue.

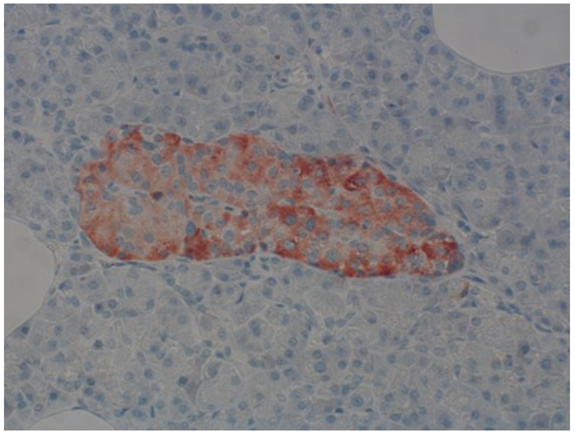

Figure SI-2: Human pancreatic tissue, stained with 1:250 dilution of anti Sst2 UMB-1 clone.

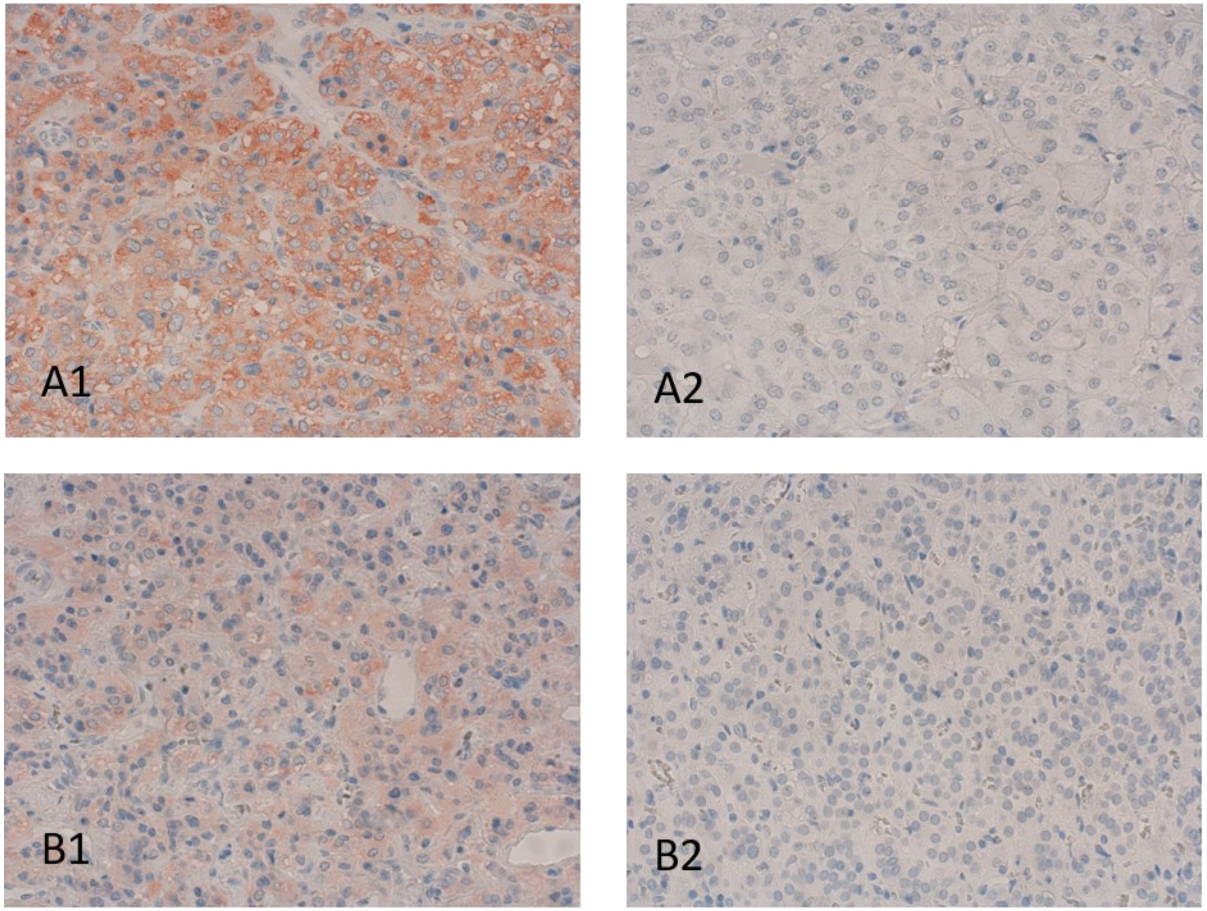

Figure SI-3: Human pheochromocytoma tissue, stained with anti Sst3 UMB-5 clone. A1- 1:1000 dilution. A2 – negative control. B1-1:5000 dilution, B2 – negative control

Manufacturer	Cat no.	Description
OriGene™	TA812061	Mouse monoclonal anti human SST3 clone OTI1A1
OriGene™	TA808922	Mouse monoclonal anti human SST3 clone OTI9A7
Abcam™	ab201952	Mouse monoclonal antibody anti human SST3 clone 7H85
Rndsystems™	MAB7018	Mouse monoclonal antibody anti human SST3 clone 576017
Abcam™	ab28680	Rabbit polyclonal serum to SST3
Bio-Rad™	MCA5921	Mouse monoclonal antibody anti human SST3
Invitrogen™	PA3-110	Rabbit polyclonal antibody anti human SST3
LSBio™	LS-A2262	Rabbit polyclonal antibody anti-human SST3
Abcam™	Ab137026	Rabbit monoclonal antibody anti human SST3 clone UMB-5

Table SI-1: Different SST3 antibodies tested for antibody selection.

				Interpretation			
Staining intensity		Fraction of stained cells		Classic	Modern	Yu et al*	Expression
**0** (no)	**X**	**1** (1-10%)	=	0-1	0-2	0-2	**Negative**
**1** (mild)		**2** (11-50%)		2-3	3-5	3-5	**Mild**
**2** (moderate)		**3** (51-80%)		4-8	6-8	6-8	**Moderate**
**3** (strong)		**4** (81-100%)		9-12	9-12	9-12	**Strong**

*Table SI-2 - IRS scoring. *same scoring system is adopted in the present manuscript, with staining between 0-5 considered "clinically negative". The full reference is found in the bibliography to the manuscript*

TMA	Position	No.	Age	Sex	Organ/Anatomic Site	Pathology diagnosis	TNM	Grade	Stage	Type	Tissue ID.	**IRS SST2**	**IRS SST3**
LY301	A1	1	75	M	Lymph node	Diffuse large B cell lymphoma of mesentery (DLBCL)				Malignant	Ily130209	2	4.5
LY301	A2	2	65	F	Lymph node	Diffuse large B cell lymphoma of left neck (DLBCL)				Malignant	Ily130198	4.5	6
LY301	A3	3	37	M	Lymph node	Diffuse large B cell lymphoma of right neck (DLBCL)				Malignant	Ily130196	5.25	5.25
LY301	A4	4	65	M	Lymph node	Diffuse large B cell lymphoma of left neck (DLBCL)				Malignant	Ily130178	10	5.25
LY301	A5	5	60	F	Lymph node	Diffuse large B cell lymphoma of mesentery (DLBCL)				Malignant	Ily130164	1	2.5
LY301	A6	6	59	F	Lymph node	Diffuse large B cell lymphoma of retroperitoneal cavity (DLBCL)				Malignant	Ily130163	4.5	4.5
LY301	B1	7	65	F	Lymph node	Diffuse large B cell lymphoma above left collarbone (DLBCL)				Malignant	Ily130159	3	9
LY301	B2	8	41	M	Lymph node	Diffuse large B cell lymphoma of right groin (DLBCL)				Malignant	Ily130151	6	4
LY301	B3	9	34	M	Lymph node	Diffuse large B cell lymphoma of left neck (DLBCL)				Malignant	Ily130142	6	5
LY301	B4	10	51	M	Lymph node	Diffuse large B cell lymphoma of right thigh (DLBCL)				Malignant	Ily130120	6	8
LY301	B5	11	31	F	Lymph node	Diffuse large B cell lymphoma of right neck (DLBCL)				Malignant	Ily130108	2.5	7
LY301	B6	12	68	M	Lymph node	Diffuse large B cell lymphoma of left groin (DLBCL)				Malignant	Ily130080	2	4
LY301	C1	13	55	F	Lymph node	Diffuse large B cell lymphoma of left armpit (DLBCL)				Malignant	Ily130076	1	3
LY301	C2	14	46	M	Lymph node	Diffuse large B cell lymphoma of left neck (DLBCL)				Malignant	Ily130056	6	8
LY301	C3	15	81	M	Lymph node	Diffuse large B cell lymphoma of right groin (DLBCL)				Malignant	Ily130050	3	6
LY301	C4	16	30	M	Lymph node	Diffuse large B cell lymphoma of left groin (DLBCL)				Malignant	Ily130045	2	4
LY301	C5	17	62	F	Lymph node	Diffuse large B cell lymphoma of right lower jaw (DLBCL)				Malignant	Ily130044	8	6
LY301	C6	18	55	M	Lymph node	Diffuse large B cell lymphoma above left collarbone (DLBCL)				Malignant	Ily130040	10	6
LY301	D1	19	77	M	Lymph node	Diffuse large B cell lymphoma of left neck (DLBCL)				Malignant	Ily130021	6	4
LY301	D2	20	44	M	Lymph node	Diffuse large B cell lymphoma of right neck (DLBCL)				Malignant	Ily130020	5.25	7
LY301	D3	21	46	M	Lymph node	Diffuse large B cell lymphoma of right neck (DLBCL)				Malignant	Ily130017	5.25	7
LY301	D4	22	59	M	Lymph node	Diffuse large B cell lymphoma of right armpit (DLBCL)				Malignant	Ily130010	7	4
LY301	D5	23	81	M	Lymph node	Diffuse large B cell lymphoma of right groin (DLBCL)				Malignant	Ily130005	4.5	7
LY301	D6	24	35	F	Lymph node	Diffuse large B cell lymphoma of armpit (DLBCL)				Malignant	Ily120004	2	6
LY301	E1	25	59	M	Lymph node	Diffuse large B cell lymphoma of right groin (DLBCL)				Malignant	Ily100011	1	4
LY301	E2	26	65	F	Lymph node	Diffuse large B cell lymphoma above left collarbone (DLBCL)				Malignant	Ily100006	4	8
LY301	E3	27	58	F	Lymph node	Diffuse large B cell lymphoma of right armpit (DLBCL)				Malignant	Ily090038	2	4.5

TMA	Posit ion	No.	Age	Sex	Organ/Anatomic Site	Pathology diagnosis	TNM	Grade	Stage	Type	Tissue ID.	**IRS SST2**	**IRS SST3**
LY301	E4	28	30	M	Lymph node	Diffuse large B cell lymphoma of right armpit (DLBCL)				Malignant	Ily090035	0	7.5
LY301	E5	29	45	F	Lymph node	Diffuse large B cell lymphoma of left groin (DLBCL)				Malignant	Ily080056	6	4
LY301	E6	30	40	F	Lymph node	Diffuse large B cell lymphoma of right neck (DLBCL)				Malignant	Ily050159	7	4
TP242f	A1	1	49	M	Transverse colon	Uplift type mucinous adenocarcinoma	T2N0M0	1	I	Malignant	Dco500270	4	4
TP242f	A2	2	54	F	Right colon	Uplift type mucinous adenocarcinoma	T3N0M0	1	IIA	Malignant	Dco500268	6	4
TP242f	A3	3	65	M	Right colon	Ulcer type adenocarcinoma	T4aN1M0	2	IIIB	Malignant	Dco500226	7	4
TP242f	A4	4	74	F	Right colon	Infiltrating type adenocarcinoma	T2N2bM0	3	IIIC	Malignant	Dco500251	4.5	8
TP242f	B1	7	45	F	Breast	Invasive carcinoma of no special type	T2N3aM0	2	IIIC	Malignant	Fmg500232	5	5
TP242f	B2	8	50	F	Breast	Invasive carcinoma of no special type	T2N1M0	2	IIB	Malignant	Fmg500264	4	7
TP242f	B3	9	49	F	Breast	Invasive carcinoma of no special type	T2N2M0	3	IIIA	Malignant	Fmg500236	2	6
TP242f	B4	10	49	F	Breast	Invasive carcinoma of no special type	T1cN0M0	3	IA	Malignant	Fmg500283	9	6
TP242f	C1	13	55	M	Lung	Papillary adenocarcinoma	T4N1M0	2	IIIA	Malignant	Rln180047	6	5.25
TP242f	C2	14	51	M	Lung	Solid adenocarcinoma	T2bN1M0	3	IIB	Malignant	Rln180040	4	6
TP242f	C3	15	73	M	Lung	Squamous cell carcinoma	T2aN0M0	1	IB	Malignant	Rln170093	4	6
TP242f	C4	16	51	F	Lung	Squamous cell carcinoma	T2aN1M0	3	IIB	Malignant	Rln170106	2	6
TP242f	D1	19	80	M	Prostate	Adenocarcinoma (Gleason grade:2, Gleason score 3+4)	T2N0M0	2	IIB	Malignant	Mpr500003	2	6
TP242f	D2	20	71	M	Prostate	Adenocarcinoma (Gleason grade:4, Gleason score 4+4)	T3bN0M0	3	IIIB	Malignant	Mpr500170	2	5
TP242f	D3	21	65	M	Prostate	Adenocarcinoma (Gleason grade:5, Gleason score 5+4)	T2N0M0	3	IIIC	Malignant	Mpr500061	0	2
TP242f	D4	22	68	M	Prostate	Adenocarcinoma (Gleason grade:5, Gleason score 5+4)	T2N0M0	3	IIIC	Malignant	Mpr500174	2	4
NE841	A1	1	49	M	Mediastinum	Atypical carcinoid				Malignant	Amd14017 6	4	4
NE841	A2	2	49	M	Mediastinum	Atypical carcinoid				Malignant	Amd14017 6	4	4
NE841	A3	3	38	M	Mediastinum	Atypical carcinoid				Malignant	Amd06036 5	4	6
NE841	A4	4	38	M	Mediastinum	Atypical carcinoid				Malignant	Amd06036 5	4	6
NE841	A5	5	61	M	Cardia	Atypical carcinoid				Malignant	Dca062166	3	4
NE841	A6	6	61	M	Cardia	Atypical carcinoid				Malignant	Dca062166	1	3
NE841	A7	7	65	F	Cardia	Carcinoid				Malignant	Dca070529	3	5
NE841	A8	8	65	F	Cardia	Carcinoid				Malignant	Dca070529	2	4

TMA	Position	No.	Age	Sex	Organ/Anatomic Site	Pathology diagnosis	TNM	Grade	Stage	Type	Tissue ID.	**IRS SST2**	**IRS SST3**
NE841	A9	9	53	M	Gallbladder	Carcinoid				Malignant	Dch061394	0	11
NE841	A10	10	53	M	Gallbladder	Carcinoid				Malignant	Dch061394	0	11
NE841	B1	11	64	F	Gallbladder	Carcinoid				Malignant	Dch140100	0	1
NE841	B2	12	64	F	Gallbladder	Carcinoid				Malignant	Dch140100	0	2
NE841	B3	13	55	M	Colon	Atypical carcinoid				Malignant	Dco080371	4	2
NE841	B4	14	55	M	Colon	Atypical carcinoid				Malignant	Dco080371	4	2
NE841	B5	15	59	M	Colon	Atypical carcinoid				Malignant	Dco080348	4	3
NE841	B6	16	59	M	Colon	Atypical carcinoid				Malignant	Dco080348	5	4
NE841	B7	17	47	F	Small intestine	Carcinoid				Malignant	Din050627	3	10
NE841	B8	18	47	F	Small intestine	Carcinoid				Malignant	Din050627	3	11
NE841	B9	19	66	M	Small intestine	Atypical carcinoid				Malignant	Din100169	0	5
NE841	B10	20	66	M	Small intestine	Atypical carcinoid				Malignant	Din100169	2	7
NE841	C1	21	42	M	Pancreas	Atypical carcinoid				Malignant	Dpa050092	4	6
NE841	C2	22	42	M	Pancreas	Atypical carcinoid				Malignant	Dpa050092	2	5
NE841	C3	23	45	F	Pancreas	Carcinoid				Malignant	Dpa090143	5	7
NE841	C4	24	45	F	Pancreas	Carcinoid				Malignant	Dpa090143	6	7
NE841	C5	25	38	M	Rectum	Carcinoid				Malignant	Dre041231	4	8
NE841	C6	26	38	M	Rectum	Carcinoid				Malignant	Dre041231	3	8
NE841	C7	27	79	M	Rectum	Carcinoid				Malignant	Dre050765	8	11
NE841	C8	28	79	M	Rectum	Carcinoid				Malignant	Dre050765	7	10
NE841	C9	29	56	F	Rectum	Carcinoid				Malignant	Dre051121	2	6
NE841	C10	30	56	F	Rectum	Carcinoid				Malignant	Dre051121	2	4
NE841	D1	31	52	F	Stomach	Carcinoid				Malignant	Dst020325	8	7
NE841	D2	32	52	F	Stomach	Carcinoid				Malignant	Dst020325	10	8
NE841	D3	33	44	M	Stomach	Atypical carcinoid				Malignant	Dst041647	5	3
NE841	D4	34	44	M	Stomach	Atypical carcinoid				Malignant	Dst041647	5	3
NE841	D5	35	52	M	Lung	Small cell carcinoma				Malignant	Rln030169	1	4
NE841	D6	36	52	M	Lung	Small cell carcinoma				Malignant	Rln030169	2	6

TMA	Position	No.	Age	Sex	Organ/Anatomic Site	Pathology diagnosis	TNM	Grade	Stage	Type	Tissue ID.	**IRS SST2**	**IRS SST3**
NE841	D7	37	56	F	Lung	Small cell carcinoma				Malignant	Rln030643	5	8
NE841	D8	38	56	F	Lung	Small cell carcinoma				Malignant	Rln030643	3	7
NE841	D9	39	58	M	Lung	Atypical carcinoid				Malignant	Rln030502	2	7
NE841	D10	40	58	M	Lung	Atypical carcinoid				Malignant	Rln030502	2	7
NE841	E1	41	39	M	Mediastinum	Squamous cell carcinoma				Malignant	Amd080023	7	4
NE841	E2	42	56	M	Mediastinum	Squamous cell carcinoma				Malignant	Amd070237	7	4
NE841	E3	43	55	F	Cardia	Adenocarcinoma				Malignant	Dca030620	4	5
NE841	E4	44	73	M	Cardia	Adenocarcinoma				Malignant	Dca031125	2	2
NE841	E5	45	78	M	Gallbladder	Adenocarcinoma				Malignant	Dch010283	7	7
NE841	E6	46	45	M	Gallbladder	Adenocarcinoma				Malignant	Dch100351	4	7
NE841	E7	47	47	M	Colon	Adenocarcinoma				Malignant	Dco060166	6	7
NE841	E9	49	87	M	Small intestine	Adenocarcinoma				Malignant	Din070072	3	1
NE841	E10	50	39	F	Small intestine	Adenocarcinoma				Malignant	Din070399	0	2
NE841	F1	51	68	F	Pancreas	Adenocarcinoma				Malignant	Dpa100492	4	2
NE841	F2	52	69	F	Pancreas	Adenocarcinoma				Malignant	Dpa010448	4	1
NE841	F3	53	60	M	Rectum	Adenocarcinoma				Malignant	Dre060053	4	5
NE841	F4	54	34	F	Rectum	Adenocarcinoma				Malignant	Dre060122	5	4.5
NE841	F5	55	65	M	Stomach	Adenocarcinoma				Malignant	Dst060101	11	8
NE841	F6	56	46	F	Stomach	Adenocarcinoma				Malignant	Dst100333	2	3
NE841	F7	57	70	M	Lung	Adenocarcinoma				Malignant	Rln060038	3	6
NE841	F8	58	59	M	Lung	Adenocarcinoma				Malignant	Rln060042	2	7
AG801	A2	2	45	M	Adrenal gland	Adrenal cortical adenocarcinoma	T2N0M0	-	II	malignant	Eag030021	3	3
AG801	A3	3	59	M	Adrenal gland	Adrenal cortical adenocarcinoma	T3N0M0	-	III	malignant	Eag030100	3	6
AG801	A4	4	58	M	Adrenal gland	Adrenal cortical adenocarcinoma	T4N0M0	-	IV	malignant	Eag030307	6	7
AG801	A5	5	27	F	Adrenal gland	Adrenal cortical adenocarcinoma	T3N0M0	-	III	malignant	Eag030364	4	1
AG801	A6	6	35	M	Adrenal gland	Adrenal cortical adenocarcinoma	T3N0M0	-	III	malignant	Eag030511	1	1
AG801	A7	7	59	M	Adrenal gland	Adrenal cortical adenocarcinoma	T3N0M0	-	III	malignant	Eag040066	0	5
AG801	A8	8	38	M	Adrenal gland	Adrenal cortical adenocarcinoma	T3N0M0	-	III	malignant	Eag050003	0	7

TMA	Position	No.	Age	Sex	Organ/Anatomic Site	Pathology diagnosis	TNM	Grade	Stage	Type	Tissue ID.	**IRS SST2**	**IRS SST3**
AG801	A9	9	10	F	Adrenal gland	Adrenal cortical adenocarcinoma	T3N0M0	-	III	malignant	Eag050100	0	1
AG801	A10	10	27	F	Adrenal gland	Adrenal cortical adenocarcinoma	T3N0M0	-	III	malignant	Eag060053	0	7
AG801	B1	11	34	F	Adrenal gland	Pheochromocytoma		-		benign	Eag020061	6	8
AG801	B2	12	51	M	Adrenal gland	Pheochromocytoma		-		benign	Eag020203	5.25	8
AG801	B3	13	26	F	Adrenal gland	Pheochromocytoma		-		benign	Eag020735	5	8
AG801	B4	14	41	F	Adrenal gland	Pheochromocytoma		-		benign	Eag020736	3	5
AG801	B5	15	20	M	Adrenal gland	Pheochromocytoma		-		benign	Eag020845	3	8
AG801	B6	16	29	F	Adrenal gland	Pheochromocytoma		-		benign	Eag020846	5	8
AG801	B7	17	39	M	Adrenal gland	Pheochromocytoma		-		benign	Eag020847	1	8
AG801	B8	18	16	F	Adrenal gland	Pheochromocytoma		-		benign	Eag030089	0	6
AG801	B9	19	39	M	Adrenal gland	Pheochromocytoma		-		benign	Eag030147	1	8
AG801	B10	20	51	F	Adrenal gland	Pheochromocytoma		-		benign	Eag030211	5.25	8
AG801	C1	21	34	M	Adrenal gland	Pheochromocytoma		-		benign	Eag030259	2	10
AG801	C2	22	65	M	Adrenal gland	Pheochromocytoma		-		benign	Eag030522	4	5
AG801	C3	23	31	F	Adrenal gland	Pheochromocytoma		-		benign	Eag030439	6	9
AG801	C4	24	52	F	Adrenal gland	Pheochromocytoma		-		benign	Eag030435	3	6
AG801	C5	25	48	M	Adrenal gland	Pheochromocytoma		-		benign	Eag030427	2	5
AG801	C6	26	56	M	Adrenal gland	Pheochromocytoma		-		benign	Eag030420	6	7
AG801	C7	27	18	M	Adrenal gland	Pheochromocytoma		-		benign	Eag030398	4.5	8
AG801	C8	28	55	M	Adrenal gland	Pheochromocytoma		-		benign	Eag030375	3	7
AG801	C9	29	28	M	Adrenal gland	Pheochromocytoma		-		benign	Eag030374	4	8
AG801	C10	30	16	F	Adrenal gland	Pheochromocytoma		-		benign	Eag030313	5.25	10
AG801	D1	31	50	F	Adrenal gland	Pheochromocytoma		-		benign	Eag040017	2	8
AG801	D2	32	45	M	Adrenal gland	Pheochromocytoma		-		benign	Eag040024	4	8
AG801	D3	33	44	M	Adrenal gland	Pheochromocytoma		-		benign	Eag040034	6	9
AG801	D4	34	46	F	Adrenal gland	Pheochromocytoma		-		benign	Eag040035	10	6
AG801	D5	35	50	F	Adrenal gland	Pheochromocytoma		-		benign	Eag040322	6	7
AG801	D6	36	43	F	Adrenal gland	Pheochromocytoma		-		benign	Eag050046	4	4

TMA	Position	No.	Age	Sex	Organ/Anatomic Site	Pathology diagnosis	TNM	Grade	Stage	Type	Tissue ID.	**IRS SST2**	**IRS SST3**
AG801	D7	37	48	F	Adrenal gland	Pheochromocytoma		-		benign	Eag050012	10	8
AG801	D8	38	38	M	Adrenal gland	Pheochromocytoma		-		benign	Eag050037	8	8
AG801	D9	39	44	F	Adrenal gland	Pheochromocytoma		-		benign	Eag050027	6	8
AG801	D10	40	34	M	Adrenal gland	Pheochromocytoma		-		benign	Eag050045	2	8
AG801	E1	41	39	F	Adrenal gland	Adrenocortical adenoma		-		benign	Eag030452	6	7
AG801	E2	42	65	F	Adrenal gland	Adrenocortical adenoma		-		benign	Eag040041	6	7
AG801	E3	43	40	F	Adrenal gland	Adrenocortical adenoma		-		benign	Eag030513	6	7
AG801	E4	44	32	F	Adrenal gland	Adrenocortical adenoma		-		benign	Eag030514	5	7
AG801	E5	45	37	F	Adrenal gland	Adrenocortical adenoma		-		benign	Eag030524	6	7
AG801	E6	46	39	F	Adrenal gland	Adrenocortical adenoma		-		benign	Eag040033	6	4
AG801	E7	47	38	F	Adrenal gland	Adrenocortical adenoma		-		benign	Eag050030	4	7
AG801	E8	48	35	M	Adrenal gland	Adrenocortical adenoma		-		benign	Eag040045	6	8
AG801	E9	49	53	F	Adrenal gland	Adrenocortical adenoma		-		benign	Eag040046	6	8
AG801	E10	50	52	M	Adrenal gland	Adrenocortical adenoma		-		benign	Eag040078	7	6
AG801	F1	51	46	M	Adrenal gland	Adrenocortical adenoma		-		benign	Eag010001	4	7
AG801	F2	52	33	F	Adrenal gland	Adrenocortical adenoma		-		benign	Eag010002	6	7
AG801	F3	53	48	M	Adrenal gland	Adrenocortical adenoma		-		benign	Eag010003	10	7
AG801	F4	54	30	F	Adrenal gland	Adrenocortical adenoma		-		benign	Eag010130	8	7
AG801	F5	55	40	M	Adrenal gland	Adrenocortical adenoma		-		benign	Eag010113	6	8
AG801	F6	56	54	M	Adrenal gland	Adrenocortical adenoma		-		benign	Eag020032	6	10
AG801	F7	57	47	F	Adrenal gland	Adrenocortical adenoma		-		benign	Eag010150	1	3
AG801	F8	58	30	F	Adrenal gland	Adrenocortical adenoma		-		benign	Eag030286	5	6
AG801	F9	59	57	F	Adrenal gland	Adrenocortical adenoma		-		benign	Eag030239	4	8
AG801	F10	60	46	M	Adrenal gland	Adrenocortical adenoma		-		benign	Eag030238	6	8
AG801	G1	61	40	M	Adrenal gland	Adrenocortical adenoma		-		benign	Eag050050	4.5	8
AG801	G2	62	50	F	Adrenal gland	Adrenocortical adenoma		-		benign	Eag050035	8	8
AG801	G3	63	62	F	Adrenal gland	Adrenocortical adenoma		-		benign	Eag050031	4	10
AG801	G4	64	30	F	Adrenal gland	Adrenocortical adenoma		-		benign	Eag010073	8	8

TMA	Position	No.	Age	Sex	Organ/Anatomic Site	Pathology diagnosis	TNM	Grade	Stage	Type	Tissue ID.	**IRS SST2**	**IRS SST3**
AG801	G5	65	67	M	Adrenal gland	Adrenocortical adenoma		-		benign	Eag030210	6	10
AG801	G6	66	35	F	Adrenal gland	Adrenocortical adenoma		-		benign	Eag030078	8	8
AG801	G7	67	27	F	Adrenal gland	Adrenocortical adenoma		-		benign	Eag021064	6	10
AG801	G8	68	32	M	Adrenal gland	Adrenocortical adenoma		-		benign	Eag020987	7	3
AG801	G9	69	30	F	Adrenal gland	Adrenocortical adenoma		-		benign	Eag020975	7	10
AG801	G10	70	30	F	Adrenal gland	Adrenocortical adenoma		-		benign	Eag020958	7	11
AG801	H1	71	67	M	Adrenal gland	Adrenocortical adenoma		-		benign	Eag030417	5.25	0
AG801	H2	72	45	F	Adrenal gland	Adrenocortical adenoma		-		benign	Eag030406	10	10
AG801	H3	73	46	F	Adrenal gland	Adrenocortical adenoma		-		benign	Eag030397	2	1
AG801	H4	74	36	F	Adrenal gland	Adrenocortical adenoma		-		benign	Eag030393	5	8
AG801	H5	75	52	F	Adrenal gland	Adrenocortical adenoma		-		benign	Eag030352	6	10
AG801	H6	76	24	F	Adrenal gland	Adrenocortical adenoma		-		benign	Eag030351	8	8.25
AG801	H7	77	32	F	Adrenal gland	Adrenocortical adenoma		-		benign	Eag030337	6	10
AG801	H8	78	50	M	Adrenal gland	Adrenocortical adenoma		-		benign	Eag030324	7	9
AG801	H9	79	46	M	Adrenal gland	Adrenocortical adenoma		-		benign	Eag030322	7	9
AG801	H10	80	41	M	Adrenal gland	Adrenocortical adenoma		-		benign	Eag030303	4	7
LV1221	A6	6	56	F	Liver	Intrahepatic cholangiocarcinoma	T2N0M0	2	II	Malignant	Dlv140267	1	6
LV1221	A8	8	64	M	Liver	Intrahepatic cholangiocarcinoma	T2N0M0	3	II	Malignant	Dlv130402	3	3
LV1221	A9	9	49	M	Liver	Intrahepatic cholangiocarcinoma	T2N0M0	3	II	Malignant	Dlv120372	3	2
LV1221	A10	10	56	M	Liver	Hepatocellular carcinoma	T3N0M0	2	IIIA	Malignant	Dlv140108	1.5	3
LV1221	A11	11	55	M	Liver	Intrahepatic cholangiocarcinoma	T3N1M0	1	IVA	Malignant	Dlv140141	4.5	4
LV1221	B1	13	62	F	Liver	Intrahepatic cholangiocarcinoma	T3N1M0	1	IVA	Malignant	Dlv140046	0	0
LV1221	B2	14	74	M	Liver	Intrahepatic cholangiocarcinoma	T3N1M0	1	IVA	Malignant	Dlv090140	1	6
LV1221	B3	15	32	F	Liver	Carcinoid	T2N1M0	-	IVA	Malignant	Dlv080059	4.5	8
LV1221	B4	16	49	M	Liver	Intrahepatic cholangiocarcinoma with necrosis	T2N1M0	2	IVA	Malignant	Dlv070581	1.5	2
LV1221	B5	17	40	M	Liver	Intrahepatic cholangiocarcinoma	T2N1M0	1	IVA	Malignant	Dlv070056	1	0
LV1221	B6	18	60	M	Liver	Intrahepatic cholangiocarcinoma	T3N1M0	1	IVA	Malignant	Dlv080175	1	4.5
LV1221	B7	19	65	F	Liver	Intrahepatic cholangiocarcinoma	T2N1M0	1	IVA	Malignant	Dlv040301	6	4

TMA	Position	No.	Age	Sex	Organ/Anatomic Site	Pathology diagnosis	TNM	Grade	Stage	Type	Tissue ID.	**IRS SST2**	**IRS SST3**
LV1221	B8	20	69	M	Liver	Intrahepatic cholangiocarcinoma	T3N1M0	1	IVA	Malignant	Dlv100144	2	5.25
LV1221	B9	21	71	M	Liver	Intrahepatic cholangiocarcinoma	T2N1M0	1	IVA	Malignant	Dlv100135	0	4
LV1221	B10	22	39	F	Liver	Intrahepatic cholangiocarcinoma	T3N1M0	1	IVA	Malignant	Dlv100131	1	4
LV1221	B11	23	70	F	Liver	Intrahepatic cholangiocarcinoma	T3N1M0	2	IVA	Malignant	Dlv090243	0	0
LV1221	C1	25	55	F	Liver	Intrahepatic cholangiocarcinoma	T2N1M0	1	IVA	Malignant	Dlv090215	1	7
LV1221	C2	26	73	M	Liver	Intrahepatic cholangiocarcinoma	T2N1M0	2	IVA	Malignant	Dlv090199	6	3
LV1221	C3	27	51	M	Liver	Intrahepatic cholangiocarcinoma	T3N1M0	-	IVA	Malignant	Dlv100160	1	2
LV1221	C4	28	69	F	Liver	Intrahepatic cholangiocarcinoma	T2N1M0	2	IVA	Malignant	Dlv090114	6	0
LV1221	C5	29	63	M	Liver	Intrahepatic cholangiocarcinoma	T2N1M0	2	IVA	Malignant	Dlv080072	7	4
LV1221	C6	30	67	F	Liver	Intrahepatic cholangiocarcinoma	T3N1M0	2	IVA	Malignant	Dlv140257	6	4
LV1221	C7	31	46	F	Liver	Intrahepatic cholangiocarcinoma	T2N1M0	2	IVA	Malignant	Dlv080019	6	3
LV1221	C8	32	54	M	Liver	Intrahepatic cholangiocarcinoma	T3N1M0	2	IVA	Malignant	Dlv140029	8	6
LV1221	C9	33	68	M	Liver	Intrahepatic cholangiocarcinoma	T3N1M0	-	IVA	Malignant	Dlv100133	4	0
LV1221	C10	34	54	F	Liver	Intrahepatic cholangiocarcinoma	T3N1M0	2	IVA	Malignant	Dlv070221	7	5
LV1221	C11	35	51	F	Liver	Intrahepatic cholangiocarcinoma	T3N1M0	-	IVA	Malignant	Dlv140105	1	4
LV1221	D1	37	52	M	Liver	Intrahepatic cholangiocarcinoma	T2N1M0	2	IVA	Malignant	Dlv070268	0	3
LV1221	D2	38	52	M	Liver	Intrahepatic cholangiocarcinoma	T3N1M0	3	IVA	Malignant	Dlv070232	1	5
LV1221	D3	39	67	M	Liver	Intrahepatic cholangiocarcinoma	T3N1M0	2	IVA	Malignant	Dlv070101	0	2.5
LV1221	D4	40	65	M	Liver	Intrahepatic cholangiocarcinoma	T3N1M0	2	IVA	Malignant	Dlv090244	6	2.5
LV1221	D5	41	60	M	Liver	Intrahepatic cholangiocarcinoma	T2N1M0	3	IVA	Malignant	Dlv080251	7	7
LV1221	D6	42	50	F	Liver	Intrahepatic cholangiocarcinoma	T3N1M0	2	IVA	Malignant	Dlv140070	6	2
LV1221	D7	43	49	M	Liver	Intrahepatic cholangiocarcinoma with necrosis	T2N1M0	3	IVA	Malignant	Dlv070459	2	6
LV1221	D8	44	64	M	Liver	Intrahepatic cholangiocarcinoma	T3N1M0	2	IVA	Malignant	Dlv080086	1	2
LV1221	D9	45	63	M	Liver	Intrahepatic cholangiocarcinoma	T3N1M0	3	IVA	Malignant	Dlv080010	4.5	6
LV1221	D10	46	45	M	Liver	Intrahepatic cholangiocarcinoma	T3N1M0	3	IVA	Malignant	Dlv090236	0	8
LV1221	D11	47	51	M	Liver	Intrahepatic cholangiocarcinoma	T3N1M0	3	IVA	Malignant	Dlv090250	2	9
LV1221	E1	49	68	M	Liver	Hepatocellular carcinoma	T3N1M0	2	IVA	Malignant	Dlv070317	2	9
LV1221	E2	50	34	M	Liver	Hepatocellular carcinoma	T2N1M0	-	IVA	Malignant	Dlv070445	0	0

TMA	Position	No.	Age	Sex	Organ/Anatomic Site	Pathology diagnosis	TNM	Grade	Stage	Type	Tissue ID.	**IRS SST2**	**IRS SST3**
LV1221	E3	51	65	M	Liver	Hepatocellular carcinoma	T2N1M0	-	IVA	Malignant	Dlv070384	4	2
LV1221	E4	52	70	F	Liver	Hepatocellular carcinoma	T3N1M0	3	IVA	Malignant	Dlv100148	1	9
LV1221	E5	53	47	F	Liver	Hepatocellular carcinoma	T2N1M0	3	IVA	Malignant	Dlv090216	0	5
LV1221	E6	54	60	F	Liver	Hepatocellular carcinoma	T3N1M0	-	IVA	Malignant	Dlv100128	0	4
LV1221	E7	55	57	F	Liver	Hepatocellular carcinoma	T2N1M0	2	IVA	Malignant	Dlv090205	1.5	4.5
LV1221	E8	56	52	F	Liver	Hepatocellular carcinoma	T3N1M0	3	IVA	Malignant	Dlv140015	7	3
LV1221	E9	57	36	F	Liver	Hepatocellular carcinoma	T2N1M0	3	IVA	Malignant	Dlv051498	6	6
LV1221	E10	58	69	M	Liver	Hepatocellular carcinoma	T3N1M0	3	IVA	Malignant	Dlv130567	3	6
LV1221	E11	59	52	M	Liver	Hepatocellular carcinoma	T3N1M0	2	IVA	Malignant	Dlv070188	4	10
LV1221	F1	61	61	M	Liver	Hepatocellular carcinoma	T3N1M0	2	IVA	Malignant	Dlv130636	2	9
LV1221	F2	62	76	M	Liver	Hepatocellular carcinoma	T3N1M0	2	IVA	Malignant	Dlv130422	1	0
LV1221	F3	63	57	M	Liver	Hepatocellular carcinoma	T3N1M0	3	IVA	Malignant	Dlv140021	4.5	4
LV1221	F4	64	40	M	Liver	Hepatocellular carcinoma	T3N1M0	2	IVA	Malignant	Dlv140328	7	4
LV1221	F5	65	64	M	Liver	Hepatocellular carcinoma	T3N1M0	2	IVA	Malignant	Dlv062329	1	1
LV1221	F6	66	52	M	Liver	Hepatocellular carcinoma	T3N1M0	3	IVA	Malignant	Dlv031147	4	8
LV1221	F7	67	52	M	Liver	Hepatocellular carcinoma with necrosis	T3N1M0	3	IVA	Malignant	Dlv062807	0	0
LV1221	F8	68	69	F	Liver	Hepatocellular carcinoma	T4N1M0	2	IVA	Malignant	Dlv011168	4	4
LV1221	F9	69	36	M	Liver	Hepatocellular carcinoma with necrosis	T3N1M0	2	IVA	Malignant	Dlv070037	5	6
LV1221	F10	70	56	M	Liver	Hepatocellular carcinoma with necrosis	T2N1M0	2	IVA	Malignant	Dlv080017	3	6
LV1221	F11	71	27	M	Liver	Hepatocellular carcinoma with necrosis	T3N1M0	2	IVA	Malignant	Dlv100149	4.5	2
LV1221	G1	73	37	M	Liver	Hepatocellular carcinoma	T4N1M0	3	IVA	Malignant	Dlv022897	2	7
LV1221	G2	74	64	M	Liver	Hepatocellular carcinoma	T2N1M0	2	IVA	Malignant	Dlv080387	5	4
LV1221	G3	75	34	M	Liver	Mixed hepatocellular carcinoma	T3N1M0	-	IVA	Malignant	Dlv140017	3	7.5
LV1221	G4	76	61	F	Liver	Mixed hepatocellular carcinoma	T2N1M0	-	IVA	Malignant	Dlv090028	5	3
LV1221	G5	77	63	M	Liver	Mixed hepatocellular carcinoma	T2N1M0	-	IVA	Malignant	Dlv090024	5	7
LV1221	G6	78	52	F	Liver	Mixed hepatocellular carcinoma	T3N1M0	-	IVA	Malignant	Dlv100158	0	2
LV1221	G7	79	30	M	Liver	Carcinoid	T3N1M0	-	IVA	Malignant	Dlv140066	1	7
LV1221	G8	80	37	F	Liver	Carcinoid	T2N1M0	-	IVA	Malignant	Dlv070356	7	7

TMA	Position	No.	Age	Sex	Organ/Anatomic Site	Pathology diagnosis	TNM	Grade	Stage	Type	Tissue ID.	**IRS SST2**	**IRS SST3**
LV1221	G9	81	56	M	Liver	Adenosquamous carcinoma	T3N1M0	-	IVA	Malignant	Dlv090231	0	1
LV1221	G10	82	58	F	Liver	Adenosquamous carcinoma	T3N1M0	-	IVA	Malignant	Dlv100146	1	2.5
LV1221	G11	83	75	F	Liver	Hepatocellular carcinoma	T3N1M0	2	IVA	Malignant	Dlv140161	1	4.5
LV1221	H1	85	52	F	Liver	Hepatocellular carcinoma	T3N1M0	3	IVA	Malignant	Dlv140062	10	1
LV1221	H2	86	56	M	Liver	Intrahepatic cholangiocarcinoma	T3N0M1	2	IV	Malignant	Dlv050242	4	4
LV1221	H3	87	56	M	Liver	Intrahepatic cholangiocarcinoma	T3N0M1	2	IV	Malignant	Dlv050242	4	0
LV1221	H4	88	54	F	Liver	Intrahepatic cholangiocarcinoma	T3N1M0	2	IV	Malignant	Dlv070221	6	4
LV1221	H5	89	54	F	Liver	Intrahepatic cholangiocarcinoma	T3N1M0	2	IV	Malignant	Dlv070221	4.5	3
LV1221	H6	90	57	M	Liver	Hepatocellular carcinoma	T4N1M1	2	IVB	Malignant	Dlv062615	1	8
LV1221	H7	91	57	M	Liver	Hepatocellular carcinoma	T4N1M1	2	IVB	Malignant	Dlv062615	0	7
LV1221	H8	92	53	M	Pancrease	Metastatic intrahepatic cholangiocarcinoma from liver	-	-	-	Metastasis	Dpa023858	7	3
LV1221	H9	93	53	M	Pancrease	Metastatic intrahepatic cholangiocarcinoma from liver	-	-	-	Metastasis	Dpa023858	7	3
LV1221	H10	94	38	F	Pancrease	Metastatic hepatocellular carcinoma from liver	-	-	-	Metastasis	Dpa011076	8	7
LV1221	H11	95	38	F	Pancrease	Metastatic hepatocellular carcinoma from liver	-	-	-	Metastasis	Dpa011076	8	8
LV1221	I1	97	56	M	Colon	Metastatic intrahepatic cholangiocarcinoma from liver	-	-	-	Metastasis	Din050242	6	3.5
LV1221	I2	98	56	M	Colon	Metastatic intrahepatic cholangiocarcinoma from liver	-	-	-	Metastasis	Din050242	6	3
LV1221	I3	99	41	M	Small intestine	Metastatic hepatocellular carcinoma from liver	-	-	-	Metastasis	Din050751	3	8
LV1221	I4	100	41	M	Small intestine	Metastatic hepatocellular carcinoma from liver	-	-	-	Metastasis	Din050751	3	8
LV1221	I5	101	59	M	Mesocolon	Metastatic hepatocellular carcinoma of adipose tissue from liver	-	-	-	Metastasis	Dco140236	6	7
LV1221	I6	102	59	M	Mesocolon	Metastatic hepatocellular carcinoma of adipose tissue from liver (sparse)	-	-	-	Metastasis	Dco140236	6	7
LV1221	I7	103	64	M	Colon	Metastatic hepatocellular carcinoma from liver	-	-	-	Metastasis	Dco060641	3	4.5
LV1221	I8	104	64	M	Colon	Metastatic hepatocellular carcinoma from liver	-	-	-	Metastasis	Dco060641	3	4
LV1221	I9	105	56	F	Colon	Metastatic hepatocellular carcinoma from liver	-	-	-	Metastasis	Dco061758	6	8
LV1221	I10	106	56	F	Colon	Metastatic hepatocellular carcinoma from liver	-	-	-	Metastasis	Dco061758	7	8
LV1221	I11	107	64	F	Lung	Metastatic hepatocellular carcinoma from liver	-	-	-	Metastasis	Rln060455	0	9
LV1221	J1	109	64	F	Lung	Metastatic hepatocellular carcinoma from liver	-	-	-	Metastasis	Rln060455	0	7
LV1221	J2	110	63	F	Lung	Metastatic hepatocellular carcinoma from liver	-	-	-	Metastasis	Rln140025	4	6
LV1221	J3	111	63	F	Lung	Metastatic hepatocellular carcinoma from liver	-	-	-	Metastasis	Rln140025	5	6

TMA	Position	No.	Age	Sex	Organ/Anatomic Site	Pathology diagnosis	TNM	Grade	Stage	Type	Tissue ID.	**IRS SST2**	**IRS SST3**
LV1221	J4	112	55	M	Chest wall	Metastatic hepatocellular carcinoma of fibrous tissue from liver	-	-	-	Metastasis	Atc040120	2	7
LV1221	J5	113	55	M	Chest wall	Metastatic hepatocellular carcinoma of fibrous tissue from liver	-	-	-	Metastasis	Atc040120	3	7
LV1221	J6	114	41	M	Pelvic cavity	Metastatic hepatocellular carcinoma from liver	-	-	-	Metastasis	Apc050106	0	7
LV1221	J7	115	41	M	Pelvic cavity	Metastatic hepatocellular carcinoma from liver	-	-	-	Metastasis	Apc050106	1	7
LV1221	J8	116	56	M	Heart	Metastatic hepatocellular carcinoma of right atrium from liver	-	-	-	Metastasis	Cht090001	4	8
LV1221	J9	117	56	M	Heart	Metastatic hepatocellular carcinoma of right atrium from liver	-	-	-	Metastasis	Cht090001	3	8
LV1221	J10	118	54	M	Brain	Metastatic hepatocellular carcinoma of right occipital lobe from liver	-	-	-	Metastasis	Nct130001	8	11
LV1221	J11	119	54	M	Brain	Metastatic hepatocellular carcinoma of right occipital lobe from liver	-	-	-	Metastasis	Nct130001	3	8
LV1221	K1	121	70	M	Brain	Metastatic hepatocellular carcinoma of left parietal lobe from liver	-	-	-	Metastasis	Nct030187	2	8
LV1221	K2	122	70	M	Brain	Metastatic hepatocellular carcinoma of left parietal lobe from liver	-	-	-	Metastasis	Nct030187	0	8
LV1221	K3	123	45	M	Bone	Metastatic hepatocellular carcinoma of rib from liver	-	-	-	Metastasis	Lbn070045	7	2
LV1221	K4	124	45	M	Bone	Metastatic hepatocellular carcinoma of rib from liver	-	-	-	Metastasis	Lbn070045	8	0
LV1221	K5	125	54	M	Bone	Metastatic hepatocellular carcinoma of rib from liver	-	-	-	Metastasis	Lbn050023	3	7
LV1221	K6	126	54	M	Bone	Metastatic hepatocellular carcinoma of rib from liver	-	-	-	Metastasis	Lbn050023	4.5	10
LV1221	K7	127	33	F	Chest wall	Metastatic hepatocellular carcinoma of fibrous tissue from liver	-	-	-	Metastasis	Sfb010162	0	5
LV1221	K8	128	33	F	Chest wall	Metastatic hepatocellular carcinoma of fibrous tissue from liver	-	-	-	Metastasis	Sfb010162	0	4
LV1221	K9	129	50	M	Spleen	Metastatic hepatocellular carcinoma from liver	-	-	-	Metastasis	Isp030653	0	2
LV1221	K10	130	50	M	Spleen	Metastatic hepatocellular carcinoma from liver	-	-	-	Metastasis	Isp030653	0	2
LV1221	K11	131	57	M	Spleen	Metastatic hepatocellular carcinoma from liver	-	-	-	Metastasis	Isp060440	0	5
LV1221	K12	132	57	M	Spleen	Metastatic hepatocellular carcinoma from liver	-	-	-	Metastasis	Isp060440	0	6
LV1021a	A1	1	52	M	Liver	Hepatocellular carcinoma (sparse)	T3N0M0	2	IIIA	Malignant	Dlv023859	4.5	6
LV1021a	A2	2	52	M	Liver	Hepatocellular carcinoma	T3N0M0	2	IIIA	Malignant	Dlv023654	0	7
LV1021a	A3	3	66	M	Liver	Hepatocellular carcinoma	T3N0M0	2	IIIA	Malignant	Dlv023861	4.5	9
LV1021a	A4	4	33	M	Liver	Hepatocellular carcinoma	T3N0M0	2	IIIA	Malignant	Dlv023923	1	2.5
LV1021a	A5	5	55	M	Liver	Hepatocellular carcinoma	T2N0M0	2	II	Malignant	Dlv024171	0	7
LV1021a	A6	6	45	M	Liver	Hepatocellular carcinoma	T2N0M0	1	II	Malignant	Dlv024308	3	7

TMA	Position	No.	Age	Sex	Organ/Anatomic Site	Pathology diagnosis	TNM	Grade	Stage	Type	Tissue ID.	**IRS SST2**	**IRS SST3**
LV1021a	A7	7	49	M	Liver	Hepatocellular carcinoma	T2N0M0	1	II	Malignant	Dlv024532	1	7
LV1021a	A8	8	68	M	Liver	Hepatocellular carcinoma	T3N0M0	2	IIIA	Malignant	Dlv030034	3	7
LV1021a	A9	9	61	M	Liver	Hepatocellular carcinoma	T1N0M0	2	I	Malignant	Dlv030801	6	7
LV1021a	A10	10	47	M	Liver	Hepatocellular carcinoma	T3N0M0	2	IIIA	Malignant	Dlv031061	2	8
LV1021a	A11	11	67	M	Liver	Hepatocellular carcinoma	T2N0M0	2	II	Malignant	Dlv031643	4	7
LV1021a	A12	12	57	M	Liver	Hepatocellular carcinoma	T3N0M0	2	IIIA	Malignant	Dlv031993	4	6
LV1021a	A13	13	46	M	Liver	Hepatocellular carcinoma	T3N0M0	1	IIIA	Malignant	Dlv023655	3	7
LV1021a	B1	14	35	M	Liver	Hepatocellular carcinoma	T3N0M0	2	IIIA	Malignant	Dlv023651	1	10
LV1021a	B2	15	36	F	Liver	Hepatocellular carcinoma	T3N0M0	2	IIIA	Malignant	Dlv024309	2	7
LV1021a	B3	16	58	M	Liver	Hepatocellular carcinoma	T2N0M0	2	II	Malignant	Dlv031833	3	3
LV1021a	B4	17	33	M	Liver	Hepatocellular carcinoma with necrosis	T3N0M0	2	IIIA	Malignant	Dlv024091	0	6
LV1021a	B5	18	63	M	Liver	Hepatocellular carcinoma	T3N0M0	1	IIIA	Malignant	Dlv031882	4.5	8
LV1021a	B6	19	32	M	Liver	Hepatocellular carcinoma	T3N0M0	2	IIIA	Malignant	Dlv023871	3	9
LV1021a	B7	20	65	M	Liver	Hepatocellular carcinoma	T3N0M0	2	IIIA	Malignant	Dlv023872	1	7
LV1021a	B8	21	71	M	Liver	Hepatocellular carcinoma	T3N0M0	2	IIIA	Malignant	Dlv023873	1	7
LV1021a	B9	22	50	M	Liver	Hepatocellular carcinoma	T2N0M0	2	II	Malignant	Dlv031874	0	3
LV1021a	B10	23	62	M	Liver	Hepatocellular carcinoma	T3N0M0	2	IIIA	Malignant	Dlv023924	2	7
LV1021a	B11	24	37	M	Liver	Hepatocellular carcinoma	T3N0M0	2	IIIA	Malignant	Dlv023867	1	8
LV1021a	B12	25	52	F	Liver	Hepatocellular carcinoma	T3N0M0	2	IIIA	Malignant	Dlv032005	3	7
LV1021a	B13	26	43	M	Liver	Hepatocellular carcinoma	T3N0M0	2	IIIA	Malignant	Dlv040123	4	10
LV1021a	C1	27	28	M	Liver	Hepatocellular carcinoma	T3N0M0	2	IIIA	Malignant	Dlv040342	2	3
LV1021a	C2	28	47	M	Liver	Hepatocellular carcinoma	T2N0M0	2	II	Malignant	Dlv024307	6	4
LV1021a	C3	29	46	M	Liver	Hepatocellular carcinoma	T3N0M0	2	IIIA	Malignant	Dlv040355	4.5	1
LV1021a	C4	30	54	M	Liver	Hepatocellular carcinoma	T3N0M0	2	IIIA	Malignant	Dlv032007	2	7
LV1021a	C5	31	39	M	Liver	Hepatocellular carcinoma	T2N0M0	2	II	Malignant	Dlv024310	4	8
LV1021a	C6	32	56	M	Liver	Hepatocellular carcinoma	T3N0M0	2	IIIA	Malignant	Dlv024437	2	9
LV1021a	C7	33	28	M	Liver	Hepatocellular carcinoma	T3N0M0	2	IIIA	Malignant	Dlv024438	4	3
LV1021a	C8	34	52	M	Liver	Hepatocellular carcinoma	T2N0M0	2	II	Malignant	Dlv020110	4	8

TMA	Position	No.	Age	Sex	Organ/Anatomic Site	Pathology diagnosis	TNM	Grade	Stage	Type	Tissue ID.	**IRS SST2**	**IRS SST3**
LV1021a	C9	35	36	M	Liver	Hepatocellular carcinoma	T3N0M0	2	IIIA	Malignant	Dlv024451	3	4.5
LV1021a	C10	36	60	M	Liver	Hepatocellular carcinoma	T2N0M0	2	II	Malignant	Dlv022890	4	7
LV1021a	C11	37	65	F	Liver	Hepatocellular carcinoma	T2N0M0	2	II	Malignant	Dlv030012	2	7
LV1021a	C12	38	62	M	Liver	Hepatocellular carcinoma	T1N0M0	2	I	Malignant	Dlv030026	5	9
LV1021a	C13	39	49	M	Liver	Hepatocellular carcinoma	T2N0M0	2	II	Malignant	Dlv030029	5	8
LV1021a	D1	40	63	M	Liver	Hepatocellular carcinoma	T2N0M0	2	II	Malignant	Dlv030030	4	10
LV1021a	D2	41	42	M	Liver	Hepatocellular carcinoma	T2N0M0	2	II	Malignant	Dlv040438	7	5
LV1021a	D3	42	46	M	Liver	Hepatocellular carcinoma	T3N0M0	2	IIIA	Malignant	Dlv023752	6	11
LV1021a	D4	43	55	F	Liver	Hepatocellular carcinoma	T3N0M0	2	IIIA	Malignant	Dlv030211	2	4.5
LV1021a	D5	44	38	M	Liver	Hepatocellular carcinoma	T3N0M0	2	IIIA	Malignant	Dlv030270	3	7
LV1021a	D6	45	35	F	Liver	Hepatocellular carcinoma	T3N0M0	2	IIIA	Malignant	Dlv023750	10	7
LV1021a	D7	46	60	M	Liver	Hepatocellular carcinoma	T2N0M0	2	II	Malignant	Dlv050816	3	7.5
LV1021a	D8	47	68	F	Liver	Hepatocellular carcinoma	T2N0M0	2	II	Malignant	Dlv030319	8	7
LV1021a	D9	48	49	F	Liver	Hepatocellular carcinoma	T2N0M0	2	II	Malignant	Dlv030617	2	4.5
LV1021a	D10	49	41	M	Liver	Hepatocellular carcinoma	T2N0M0	2	II	Malignant	Dlv030759	3	3
LV1021a	D11	50	50	M	Liver	Hepatocellular carcinoma	T2N0M0	2	II	Malignant	Dlv030787	4	4
LV1021a	D12	51	34	M	Liver	Hepatocellular carcinoma	T3N0M0	2	IIIA	Malignant	Dlv040279	2	10
LV1021a	D13	52	38	M	Liver	Hepatocellular carcinoma	T3N0M0	2	IIIA	Malignant	Dlv040315	4.5	11
LV1021a	E1	53	52	F	Liver	Hepatocellular carcinoma	T3N0M0	3	IIIA	Malignant	Dlv030850	5	6
LV1021a	E2	54	48	M	Liver	Hepatocellular carcinoma	T3N0M0	2	IIIA	Malignant	Dlv040086	1	7
LV1021a	E3	55	58	M	Liver	Hepatocellular carcinoma	T3N0M0	2	IIIA	Malignant	Dlv031052	10	11
LV1021a	E4	56	42	M	Liver	Hepatocellular carcinoma	T3N0M0	2	IIIA	Malignant	Dlv040053	3	4.5
LV1021a	E5	57	41	M	Liver	Hepatocellular carcinoma	T3N0M0	2	IIIA	Malignant	Dlv031063	2	4
LV1021a	E6	58	50	M	Liver	Hepatocellular carcinoma	T3N0M0	2	IIIA	Malignant	Dlv031079	2	4.5
LV1021a	E7	59	51	F	Liver	Hepatocellular carcinoma	T3N0M0	2	IIIA	Malignant	Dlv031099	4	7
LV1021a	E8	60	37	M	Liver	Hepatocellular carcinoma	T3N0M0	2	IIIA	Malignant	Dlv040016	6	6
LV1021a	E9	61	49	M	Liver	Hepatocellular carcinoma	T3N0M0	2	IIIA	Malignant	Dlv040015	1	2
LV1021a	E10	62	58	M	Liver	Hepatocellular carcinoma	T3N1M0	2	IIIC	Malignant	Dlv031127	0	7

TMA	Posit ion	No.	Age	Sex	Organ/Anatomic Site	Pathology diagnosis	TNM	Grade	Stage	Type	Tissue ID.	**IRS SST2**	**IRS SST3**
LV1021a	E11	63	55	M	Liver	Hepatocellular carcinoma	T3N0M0	2	IIIA	Malignant	Dlv032020	2	7
LV1021a	E12	64	40	M	Liver	Hepatocellular carcinoma with necrosis	T2N0M0	2	II	Malignant	Dlv022889	10	10
LV1021a	E13	65	39	M	Liver	Hepatocellular carcinoma	T2N0M0	2	II	Malignant	Dlv040396	6	3.5
LV1021a	F1	66	49	M	Liver	Hepatocellular carcinoma	T3N0M0	2	IIIA	Malignant	Dlv031311	4	11
LV1021a	F2	67	60	F	Liver	Hepatocellular carcinoma	T3N0M0	2	IIIA	Malignant	Dlv040275	5	7
LV1021a	F3	68	60	F	Liver	Hepatocellular carcinoma	T3N0M0	2	IIIA	Malignant	Dlv031329	0	6
LV1021a	F4	69	57	M	Liver	Hepatocellular carcinoma	T2N0M0	2	II	Malignant	Dlv031459	0	9
LV1021a	F5	70	41	M	Liver	Hepatocellular carcinoma	T3N0M0	3	IIIA	Malignant	Dlv031051	4	8
LV1021a	F6	71	57	M	Liver	Hepatocellular carcinoma	T3N0M0	3	IIIA	Malignant	Dlv023862	3	2
LV1021a	F7	72	48	F	Liver	Hepatocellular carcinoma	T3N0M0	3	IIIA	Malignant	Dlv023869	0	6
LV1021a	F8	73	54	M	Liver	Hepatocellular carcinoma	T3N0M0	3	IIIA	Malignant	Dlv024182	5	7
LV1021a	F9	74	65	F	Liver	Hepatocellular carcinoma	T3N0M0	3	IIIA	Malignant	Dlv031198	0	4
LV1021a	F10	75	46	F	Liver	Hepatocellular carcinoma	T3N0M0	3	IIIA	Malignant	Dlv030285	4	6
LV1021a	F11	76	50	M	Liver	Hepatocellular carcinoma	T3N0M0	3	IIIA	Malignant	Dlv020108	1	7
LV1021a	F12	77	43	M	Liver	Hepatocellular carcinoma	T2N0M0	3	II	Malignant	Dlv031995	4	9
LV1021a	F13	78	33	M	Liver	Hepatocellular carcinoma	T3N0M0	3	IIIA	Malignant	Dlv031799	3	7
LV1021a	G1	79	43	M	Liver	Hepatocellular carcinoma	T3N0M0	3	IIIA	Malignant	Dlv024090	8	7
LV1021a	G2	80	32	M	Liver	Hepatocellular carcinoma with necrosis	T3N0M0	3	IIIA	Malignant	Dlv023870	3	7
LV1021a	G3	81	43	M	Liver	Hepatocellular carcinoma	T3N0M0	3	IIIA	Malignant	Dlv023789	3	8
LV1021a	G4	82	51	M	Liver	Hepatocellular carcinoma	T2N0M0	3	II	Malignant	Dlv031996	2	4.5
LV1021a	G5	83	43	M	Liver	Hepatocellular carcinoma (blank)	T3N0M0	*	IIIA	Malignant	Dlv031463	1	7
LV1021a	G6	84	39	M	Liver	Hepatocellular carcinoma	T3N0M0	3	IIIA	Malignant	Dlv010007	8	5
LV1021a	G7	85	28	M	Liver	Hepatocellular carcinoma	T1N0M0	3	I	Malignant	Dlv032029	4	7
LV1021a	G8	86	40	M	Liver	Hepatocellular carcinoma	T3N0M0	3	IIIA	Malignant	Dlv022892	4	7
LV1021a	G9	87	45	M	Liver	Hepatocellular carcinoma	T2N0M0	3	II	Malignant	Dlv030804	6	6
LV1021a	G10	88	55	M	Liver	Hepatocellular carcinoma	T3N0M0	2--3	IIIA	Malignant	Dlv050429	6	5
LV1021a	G11	89	69	M	Liver	Hepatocellular carcinoma	T3N0M0	3	IIIA	Malignant	Dlv031105	2	8
LV1021a	G12	90	53	M	Liver	Hepatocellular carcinoma	T2N0M0	3	II	Malignant	Dlv040056	0	8

TMA	Position	No.	Age	Sex	Organ/Anatomic Site	Pathology diagnosis	TNM	Grade	Stage	Type	Tissue ID.	**IRS SST2**	**IRS SST3**
LV1021a	G13	91	70	M	Liver	Hepatocellular carcinoma	T2N0M0	3	II	Malignant	Dlv040063	0	6
LV1021a	H1	92	37	M	Liver	Hepatocellular carcinoma	T3N0M0	3	IIIA	Malignant	Dlv040071	1	4.5
LV1021a	H2	93	61	M	Liver	Hepatocellular carcinoma	T2N0M0	3	II	Malignant	Dlv031113	5	7
LV1021a	H3	94	47	M	Liver	Hepatocellular carcinoma	T3N0M0	3	IIIA	Malignant	Dlv031977	6	6
LV1021a	H4	95	63	F	Liver	Hepatocellular carcinoma	T3N0M0	3	IIIA	Malignant	Dlv040127	6	10
LV1021a	H5	96	59	M	Liver	Hepatocellular carcinoma	T3N0M0	3	IIIA	Malignant	Dlv040314	1	10
LV1021a	H6	97	59	M	Liver	Hepatocellular carcinoma	T3N0M0	3	IIIA	Malignant	Dlv030124	10	6
BO2081	A1	1	28	M	Bone	Osteosarcoma of left femur		-		malignant	Lbn020010	2	6
BO2081	A2	2	28	M	Bone	Osteosarcoma of left femur		-		malignant	Lbn020010	6	7
BO2081	A3	3	16	M	Bone	Osteosarcoma of right femur		-		malignant	Lbn020024	2	4
BO2081	A4	4	16	M	Bone	Osteosarcoma of right femur		-		malignant	Lbn020024	2	7
BO2081	A5	5	10	F	Bone	Osteosarcoma of right femur		-		malignant	Lbn030023	4	6
BO2081	A6	6	10	F	Bone	Osteosarcoma of right femur		-		malignant	Lbn030023	4	6
BO2081	A7	7	7	F	Bone	Osteosarcoma of right femur inferior extremity		-		malignant	Lbn020013	6	4.5
BO2081	A8	8	7	F	Bone	Osteosarcoma of right femur inferior extremity		-		malignant	Lbn020013	6	4.5
BO2081	A9	9	16	M	Bone	Osteosarcoma of right femur		-		malignant	Lbn030036	6	7
BO2081	A10	10	16	M	Bone	Osteosarcoma of right femur		-		malignant	Lbn030036	6	7
BO2081	A11	11	18	M	Bone	Osteosarcoma of thoracic vertebrae		-		malignant	Lbn030035	6	8
BO2081	A12	12	18	M	Bone	Osteosarcoma of thoracic vertebrae		-		malignant	Lbn030035	4.5	7
BO2081	A13	13	19	F	Bone	Osteosarcoma of right tibia superior extremity		-		malignant	Lbn020009	1	9
BO2081	A14	14	19	F	Bone	Osteosarcoma of right tibia superior extremity		-		malignant	Lbn020009	0	9
BO2081	A15	15	12	F	Bone	Osteosarcoma of right tibia		-		malignant	Lbn030028	7.5	7.5
BO2081	A16	16	12	F	Bone	Osteosarcoma of right tibia		-		malignant	Lbn030028	4.5	8
BO2081	B1	17	23	F	Bone	Osteosarcoma of left femur		-		malignant	Lbn020076	4	8
BO2081	B2	18	23	F	Bone	Osteosarcoma of left femur		-		malignant	Lbn020076	4	7
BO2081	B3	19	14	M	Bone	Osteosarcoma of right femur inferior extremity		-		malignant	Lbn020066	6	4
BO2081	B4	20	14	M	Bone	Osteosarcoma of right femur inferior extremity		-		malignant	Lbn020066	4.5	2.5
BO2081	B5	21	30	M	Bone	Osteosarcoma of right tibia		-		malignant	Lbn020039	0	1

TMA	Position	No.	Age	Sex	Organ/Anatomic Site	Pathology diagnosis	TNM	Grade	Stage	Type	Tissue ID.	**IRS SST2**	**IRS SST3**
BO2081	B6	22	30	M	Bone	Osteosarcoma of right tibia		-		malignant	Lbn020039	0	0
BO2081	B7	23	16	M	Bone	Osteosarcoma of right femur		-		malignant	Lbn020038	3	8
BO2081	B8	24	16	M	Bone	Osteosarcoma of right femur		-		malignant	Lbn020038	4	8
BO2081	B9	25	13	F	Bone	Osteosarcoma (skeletal muscle) of left femur inferior extremity		-		malignant	Lbn020034	6	11
BO2081	B10	26	13	F	Bone	Osteosarcoma (skeletal muscle) of left femur inferior extremity		-		malignant	Lbn020034	6	10
BO2081	B11	27	15	F	Bone	Osteosarcoma of left knee		-		malignant	Lbn030006	3	10
BO2081	B12	28	15	F	Bone	Osteosarcoma of left knee		-		malignant	Lbn030006	2	6
BO2081	B13	29	39	M	Bone	Osteosarcoma of right upper arm		-		malignant	Lbn030018	3	7
BO2081	B14	30	39	M	Bone	Osteosarcoma of right upper arm		-		malignant	Lbn030018	4.5	7
BO2081	B15	31	29	M	Bone	Osteosarcoma of right humerus inferior segment		-		malignant	Lbn030003	10	7
BO2081	B16	32	29	M	Bone	Osteosarcoma (sparse) of right humerus inferior segment		-		malignant	Lbn030003	11	10
BO2081	C1	33	69	M	Bone	Osteosarcoma of right femur		-		malignant	Lbn030017	6	7
BO2081	C2	34	69	M	Bone	Osteosarcoma of right femur		-		malignant	Lbn030017	6	7
BO2081	C3	35	19	M	Bone	Osteosarcoma of left fibula		-		malignant	Lbn020075	4	8
BO2081	C4	36	19	M	Bone	Osteosarcoma of left fibula		-		malignant	Lbn020075	4	8
BO2081	C5	37	23	F	Bone	Osteosarcoma with calcification above left knee		-		malignant	Lbn020015	6	8
BO2081	C6	38	23	F	Bone	Osteosarcoma with calcification above left knee		-		malignant	Lbn020015	4	8
BO2081	C7	39	22	M	Bone	Osteosarcoma of left femur inferior extremity		-		malignant	Lbn010007	7	4.5
BO2081	C8	40	22	M	Bone	Osteosarcoma of left femur inferior extremity		-		malignant	Lbn010007	5.25	5
BO2081	C9	41	18	M	Bone	Osteosarcoma		-		malignant	Lbn010021	7	7
BO2081	C10	42	18	M	Bone	Osteosarcoma		-		malignant	Lbn010021	7	8
BO2081	C11	43	14	F	Bone	Osteosarcoma of left face		-		malignant	Lbn020002	4	7
BO2081	C12	44	14	F	Bone	Osteosarcoma of left face		-		malignant	Lbn020002	3	7
BO2081	C13	45	12	M	Bone	Osteosarcoma of left femur inferior extremity		-		malignant	Lbn020058	2	8
BO2081	C14	46	12	M	Bone	Osteosarcoma of left femur inferior extremity		-		malignant	Lbn020058	2	8
BO2081	C15	47	37	M	Bone	Osteosarcoma of left femur		-		malignant	Lbn030016	4.5	8
BO2081	C16	48	37	M	Bone	Osteosarcoma of left femur		-		malignant	Lbn030016	3	9
BO2081	D1	49	37	F	Bone	Osteosarcoma of right femur inferior extremity		-		malignant	Lbn020043	7	6

TMA	Position	No.	Age	Sex	Organ/Anatomic Site	Pathology diagnosis	TNM	Grade	Stage	Type	Tissue ID.	**IRS SST2**	**IRS SST3**
BO2081	D2	50	37	F	Bone	Osteosarcoma of right femur inferior extremity		-		malignant	Lbn020043	7	6
BO2081	D3	51	62	F	Bone	Parosteal osteosarcoma of left thigh		-		malignant	Lbn020028	7	6
BO2081	D4	52	62	F	Bone	Parosteal osteosarcoma of left thigh		-		malignant	Lbn020028	7	8
BO2081	D5	53	50	M	Bone	Chondrosarcoma of costal bone		-		malignant	Lbn020003	0	4
BO2081	D6	54	50	M	Bone	Chondrosarcoma of costal bone		-		malignant	Lbn020003	0	4
BO2081	D7	55	14	M	Bone	Chondrosarcoma of right femur inferior extremity		-		malignant	Lbn020016	3	8
BO2081	D8	56	14	M	Bone	Chondrosarcoma of right femur inferior extremity		-		malignant	Lbn020016	3	8
BO2081	D9	57	42	M	Bone	Chondrosarcoma of right tibia superior extremity		-		malignant	Lbn020068	4.5	4
BO2081	D10	58	42	M	Bone	Chondrosarcoma of right tibia superior extremity		-		malignant	Lbn020068	4.5	4
BO2081	D11	59	13	M	Bone	Chondrosarcoma of right ilium		-		malignant	Lbn030008	1	9
BO2081	D12	60	13	M	Bone	Chondrosarcoma of right ilium		-		malignant	Lbn030008	1	11
BO2081	D13	61	16	M	Bone	Chondrosarcoma of tibia superior extremity		-		malignant	Lbn030012	4	7
BO2081	D14	62	16	M	Bone	Chondrosarcoma of tibia superior extremity		-		malignant	Lbn030012	4	8
BO2081	D15	63	47	F	Bone	Chondrosarcoma of left femur inferior extremity		-		malignant	Lbn040088	1	0
BO2081	D16	64	47	F	Bone	Chondrosarcoma of left femur inferior extremity		-		malignant	Lbn040088	1	0
BO2081	E1	65	13	F	Bone	Chondrosarcoma of left tibia		-		malignant	Lbn030093	4.5	8
BO2081	E2	66	13	F	Bone	Chondrosarcoma of left tibia		-		malignant	Lbn030093	4.5	8
BO2081	E3	67	44	M	Bone	Chondrosarcoma of left upper limb		-		malignant	Lbn040112	5.25	8
BO2081	E4	68	44	M	Bone	Chondrosarcoma of left upper limb		-		malignant	Lbn040112	7	7
BO2081	E5	69	45	M	Bone	Chondrosarcoma of left femur inferior extremity		-		malignant	Lbn040095	4.5	8
BO2081	E6	70	45	M	Bone	Chondrosarcoma of left femur inferior extremity		-		malignant	Lbn040095	3.75	8
BO2081	E7	71	47	M	Bone	Chondrosarcoma of sacrum		-		malignant	Lbn010023	1	2.5
BO2081	E8	72	47	M	Bone	Chondrosarcoma of sacrum		-		malignant	Lbn010023	3	5
BO2081	E9	73	44	F	Bone	Plasma cell myeloma of fourth costal bone		-		malignant	Lbn060036	8	8
BO2081	E10	74	44	F	Bone	Plasma cell myeloma of fourth costal bone		-		malignant	Lbn060036	10	8
BO2081	E11	75	69	M	Bone	Plasma cell myeloma of left clavicle		-		malignant	Lbn060009	2	0
BO2081	E12	76	69	M	Bone	Plasma cell myeloma of left clavicle		-		malignant	Lbn060009	3	0
BO2081	E13	77	39	M	Bone	Plasma cell myeloma of eighth thoracic vertebra		-		malignant	Lbn050004	9	7

TMA	Position	No.	Age	Sex	Organ/Anatomic Site	Pathology diagnosis	TNM	Grade	Stage	Type	Tissue ID.	**IRS SST2**	**IRS SST3**
BO2081	E14	78	39	M	Bone	Plasma cell myeloma of eighth thoracic vertebra		-		malignant	Lbn050004	9	7
BO2081	E15	79	57	M	Bone	Plasma cell myeloma of right pubis		-		malignant	Lbn060013	10	6
BO2081	E16	80	57	M	Bone	Plasma cell myeloma of right pubis		-		malignant	Lbn060013	11	7
BO2081	F1	81	64	M	Bone	Plasma cell myeloma of right clavicle		-		malignant	Lbn040065	8	0
BO2081	F2	82	64	M	Bone	Plasma cell myeloma of right clavicle		-		malignant	Lbn040065	10	1
BO2081	F3	83	50	M	Bone	Plasma cell myeloma of sixth costal bone		-		malignant	Lbn040091	0	0
BO2081	F4	84	50	M	Bone	Plasma cell myeloma of sixth costal bone		-		malignant	Lbn040091	1	2
BO2081	F5	85	57	M	Bone	Plasma cell myeloma of sternum		-		malignant	Lbn030119	7	7
BO2081	F6	86	57	M	Bone	Plasma cell myeloma of sternum		-		malignant	Lbn030119	8	8
BO2081	F7	87	46	F	Bone	Plasma cell myeloma of third costal bone		-		malignant	Ibm060024	1	0
BO2081	F8	88	46	F	Bone	Plasma cell myeloma of third costal bone		-		malignant	Ibm060024	1	0
BO2081	F9	89	58	M	Bone	Plasma cell myeloma of sternum		-		malignant	Ibm030703	5	7
BO2081	F10	90	58	M	Bone	Plasma cell myeloma of sternum		-		malignant	Ibm030703	6	6
BO2081	F11	91	94	F	Bone	Ewing's sarcoma of right ilium		-		malignant	Lbn050107	3	5
BO2081	F12	92	94	F	Bone	Ewing's sarcoma of right ilium		-		malignant	Lbn050107	2	6
BO2081	F13	93	15	F	Bone	Ewing's sarcoma (sparse) of pelvis		-		malignant	Lbn050024	7	4
BO2081	F14	94	15	F	Bone	Ewing's sarcoma of pelvis		-		malignant	Lbn050024	6	6
BO2081	F15	95	32	M	Bone	Giant cell tumor of right tibia superior extremity		-		undetermined malignant potential	Lbn030086	9	6
BO2081	F16	96	32	M	Bone	Giant cell tumor of right tibia superior extremity		-		undetermined malignant potential	Lbn030086	10	8
BO2081	G1	97	17	F	Bone	Giant cell tumor of left femur		-		undetermined malignant potential	Lbn020053	6.25	7
BO2081	G2	98	17	F	Bone	Giant cell tumor of left femur		-		undetermined malignant potential	Lbn020053	6.25	6
BO2081	G3	99	42	F	Bone	Giant cell tumor of humerus		-		undetermined malignant potential	Lbn020079	8	7
BO2081	G4	100	42	F	Bone	Giant cell tumor of humerus		-		undetermined malignant potential	Lbn020079	8	7

TMA	Position	No.	Age	Sex	Organ/Anatomic Site	Pathology diagnosis	TNM	Grade	Stage	Type	Tissue ID.	**IRS SST2**	**IRS SST3**
BO2081	G5	101	60	F	Bone	Giant cell tumor of left maxilla		-		undetermined malignant potential	Lbn030060	10	6
BO2081	G6	102	60	F	Bone	Giant cell tumor of left maxilla		-		undetermined malignant potential	Lbn030060	10	7
BO2081	G7	103	24	F	Bone	Giant cell tumor of right humerus		-		undetermined malignant potential	Lbn030045	8	8
BO2081	G8	104	24	F	Bone	Giant cell tumor of right humerus		-		undetermined malignant potential	Lbn030045	8	8
BO2081	G9	105	38	F	Bone	Giant cell tumor of left radius		-		undetermined malignant potential	Lbn030031	7	7
BO2081	G10	106	38	F	Bone	Giant cell tumor of left radius		-		undetermined malignant potential	Lbn030031	8	8
BO2081	G11	107	34	M	Bone	Giant cell tumor of right tibia		-		undetermined malignant potential	Lbn030019	8	7
BO2081	G12	108	34	M	Bone	Giant cell tumor of right tibia		-		undetermined malignant potential	Lbn030019	8	7
BO2081	G13	109	45	F	Bone	Giant cell tumor of left tibia		-		undetermined malignant potential	Lbn030004	7	8
BO2081	G14	110	45	F	Bone	Giant cell tumor of left tibia		-		undetermined malignant potential	Lbn030004	7	7
BO2081	G15	111	33	F	Bone	Giant cell tumor of right femur		-		undetermined malignant potential	Lbn020050	8	8
BO2081	G16	112	33	F	Bone	Giant cell tumor of right femur		-		undetermined malignant potential	Lbn020050	8	10
BO2081	H1	113	40	F	Bone	Giant cell tumor of right radius		-		undetermined malignant potential	Lbn030063	8	8
BO2081	H2	114	40	F	Bone	Giant cell tumor of right radius		-		undetermined malignant potential	Lbn030063	8	7
BO2081	H3	115	33	M	Bone	Giant cell tumor (necrotic tissue) of right humerus		-		undetermined malignant potential	Lbn020057	6	6

TMA	Position	No.	Age	Sex	Organ/Anatomic Site	Pathology diagnosis	TNM	Grade	Stage	Type	Tissue ID.	**IRS SST2**	**IRS SST3**
BO2081	H4	116	33	M	Bone	Giant cell tumor (sparse) with necrosis of right humerus		-		undetermined malignant potential	Lbn020057	6	4.5
BO2081	H5	117	36	F	Bone	Giant cell tumor of right tibia		-		undetermined malignant potential	Lbn020069	8	7
BO2081	H6	118	36	F	Bone	Giant cell tumor of right tibia		-		undetermined malignant potential	Lbn020069	7	7
BO2081	H7	119	28	M	Bone	Giant cell tumor of left clavicle		-		undetermined malignant potential	Lbn020048	8	7
BO2081	H8	120	28	M	Bone	Giant cell tumor of left clavicle		-		undetermined malignant potential	Lbn020048	8	8
BO2081	H9	121	48	F	Bone	Giant cell tumor of left femur		-		undetermined malignant potential	Lbn020051	5	8
BO2081	H10	122	48	F	Bone	Giant cell tumor of left femur		-		undetermined malignant potential	Lbn020051	4	7
BO2081	H11	123	23	M	Bone	Giant cell tumor of right femur inferior extremity		-		undetermined malignant potential	Lbn020060	4	4.5
BO2081	H12	124	23	M	Bone	Giant cell tumor (fibrous tissue and blood vessel) of right femur inferior extremity		-		undetermined malignant potential	Lbn020060	6	10
BO2081	H13	125	34	M	Bone	Giant cell tumor of sacrum		-		undetermined malignant potential	Lbn030064	5.25	4
BO2081	H14	126	34	M	Bone	Giant cell tumor of sacrum		-		undetermined malignant potential	Lbn030064	5.25	4
BO2081	H15	127	50	M	Bone	Giant cell tumor of right femoral metaphysis		-		undetermined malignant potential	Lbn010019	7	8
BO2081	H16	128	50	M	Bone	Giant cell tumor of right femoral metaphysis		-		undetermined malignant potential	Lbn010019	8	10
BO2081	I1	129	38	F	Bone	Giant cell tumor of humeral metaphysis		-		undetermined malignant potential	Lbn020017	6	8
BO2081	I2	130	38	F	Bone	Giant cell tumor of humeral metaphysis		-		undetermined malignant potential	Lbn020017	6	8

TMA	Position	No.	Age	Sex	Organ/Anatomic Site	Pathology diagnosis	TNM	Grade	Stage	Type	Tissue ID.	**IRS SST2**	**IRS SST3**
BO2081	I3	131	47	M	Bone	Giant cell tumor of right tibial metaphysis		-		undetermined malignant potential	Lbn010004	6	7
BO2081	I4	132	47	M	Bone	Giant cell tumor of right tibial metaphysis		-		undetermined malignant potential	Lbn010004	7	8
BO2081	I5	133	20	M	Bone	Giant cell tumor of right femoral metaphysis		-		undetermined malignant potential	Lbn010013	6	7
BO2081	I6	134	20	M	Bone	Giant cell tumor of right femoral metaphysis		-		undetermined malignant potential	Lbn010013	6	7
BO2081	I7	135	44	M	Bone	Chordoma of sacrum		-		malignant	Lbn020042	5	4
BO2081	I8	136	44	M	Bone	Chordoma of sacrum		-		malignant	Lbn020042	5	4
BO2081	I9	137	50	F	Bone	Chordoma of sacrum		-		malignant	Lbn040078	0	3
BO2081	I10	138	50	F	Bone	Chordoma of sacrum		-		malignant	Lbn040078	0	2
BO2081	I11	139	11	M	Mandible	Adamantinoma of mandible		-		benign	Doc023856	2	4
BO2081	I12	140	11	M	Mandible	Adamantinoma of mandible		-		benign	Doc023856	0	3
BO2081	I13	141	28	M	Mandible	Squamosu cell adamantinoma of left mandible		-		benign	Doc030652	2	8
BO2081	I14	142	28	M	Mandible	Squamosu cell adamantinoma of left mandible		-		benign	Doc030652	2	11
BO2081	I15	143	64	F	Bone	Adamantinoma of right tibia		-		malignant	Lbn050018	4	8
BO2081	I16	144	64	F	Bone	Adamantinoma of right tibia		-		malignant	Lbn050018	4	8
BO2081	J1	145	44	M	Bone	Squamous cell adamantinoma of right fibula		-		malignant	Lbn030144	2	4.5
BO2081	J2	146	44	M	Bone	Squamous cell adamantinoma of right fibula		-		malignant	Lbn030144	3	6
BO2081	J3	147	34	M	Mandible	Adamantinoma of left mandible		-		benign	Doc031074	3	6
BO2081	J4	148	34	M	Mandible	Adamantinoma of left mandible		-		benign	Doc031074	4	6
BO2081	J5	149	40	M	Mandible	Adamantinoma of mandible		-		benign	Doc031673	2	4.5
BO2081	J6	150	40	M	Mandible	Adamantinoma of mandible		-		benign	Doc031673	0	4.5
BO2081	J7	151	47	F	Mandible	Adamantinoma of mandible		-		benign	Doc031680	2	7
BO2081	J8	152	47	F	Mandible	Adamantinoma of mandible		-		benign	Doc031680	2	7
BO2081	J9	153	70	F	Mandible	Adamantinoma of right mandible		-		benign	Doc041150	0	1
BO2081	J10	154	70	F	Mandible	Adamantinoma of right mandible		-		benign	Doc041150	1	2
BO2081	J11	155	27	M	Bone	Invasive osteoblastoma (sparse) of right femur		-		malignant	Lbn020074	3	4.5

TMA	Position	No.	Age	Sex	Organ/Anatomic Site	Pathology diagnosis	TNM	Grade	Stage	Type	Tissue ID.	**IRS SST2**	**IRS SST3**
BO2081	J12	156	27	M	Bone	Invasive osteoblastoma (sparse) of right femur		-		malignant	Lbn020074	3	4.5
BO2081	J13	157	12	M	Bone	Chondroma of left femur inferior extremity		-		benign	Lbn010016	4.5	2
BO2081	J14	158	12	M	Bone	Chondroma of left femur inferior extremity		-		benign	Lbn010016	6	2
BO2081	J15	159	14	F	Cartilage	Chondroma of chest wall		-		benign	Lca030102	6	2
BO2081	J16	160	14	F	Cartilage	Chondroma of chest wall		-		benign	Lca030102	6	2
BO2081	K1	161	39	M	Cartilage	Osteochondroma of right femur		-		benign	Lca030106	4.5	4
BO2081	K2	162	39	M	Cartilage	Osteochondroma of right femur		-		benign	Lca030106	4.5	3
BO2081	K3	163	27	M	Bone	Osteochondroma of right tibia superior extremity		-		benign	Lbn020044	2	11
BO2081	K4	164	27	M	Bone	Osteochondroma of right tibia superior extremity		-		benign	Lbn020044	2	12
BO2081	K5	165	46	F	Bone	Osteochondroma (fibrous tissue)		-		benign	Lbn030046	0	12
BO2081	K6	166	46	F	Bone	Osteochondroma (bone tissue)		-		benign	Lbn030046	0	12
BO2081	K9	169	29	M	Cartilage	Chondroblastoma of right femur inferior extremity		-		benign	Lca050080	4	7
BO2081	K10	170	29	M	Cartilage	Chondroblastoma of right femur inferior extremity		-		benign	Lca050080	3.5	7
BO2081	K11	171	22	M	Bone	Osteofibrous dysplasia of left fourth costal bone		-		benign	Lbn020011	2	7
BO2081	K12	172	22	M	Bone	Osteofibrous dysplasia of left fourth costal bone		-		benign	Lbn020011	2	7
BO2081	K13	173	38	F	Bone	Osteofibrous dysplasia of sixth costal bone		-		benign	Lbn020046	3	8
BO2081	K14	174	38	F	Bone	Osteofibrous dysplasia of sixth costal bone		-		benign	Lbn020046	3	8
BO2081	K16	176	25	F	Bone	Osteofibrous dysplasia of left femur		-		benign	Lbn020049	2	10
BO2081	L1	177	45	F	Bone	Aneurysmal bone cyst of right tibia		-		benign	Lbn030079	3.75	7
BO2081	L2	178	45	F	Bone	Aneurysmal bone cyst of right tibia		-		benign	Lbn030079	3.75	8
BO2081	L3	179	16	M	Bone	Aneurysmal bone cyst of right ankle		-		benign	Lbn030043	4.5	7
BO2081	L4	180	16	M	Bone	Aneurysmal bone cyst of right ankle		-		benign	Lbn030043	5	7
BO2081	L5	181	16	F	Bone	Aneurysmal bone cyst of left tibia superior extremity		-		benign	Lbn010015	5	7
BO2081	L6	182	16	F	Bone	Aneurysmal bone cyst of left tibia superior extremity		-		benign	Lbn010015	6	7
BO2081	L7	183	30	F	Bone	Aneurysmal bone cyst of femur inferior extremity		-		benign	Lbn040108	7	8
BO2081	L8	184	30	F	Bone	Aneurysmal bone cyst of femur inferior extremity		-		benign	Lbn040108	5	7
BO2081	L9	185	25	F	Bone	Aneurysmal bone cyst of left femur		-		benign	Lbn020041	2	8
BO2081	L10	186	25	F	Bone	Aneurysmal bone cyst of left femur		-		benign	Lbn020041	2	7

TMA	Position	No.	Age	Sex	Organ/Anatomic Site	Pathology diagnosis	TNM	Grade	Stage	Type	Tissue ID.	**IRS SST2**	**IRS SST3**
BO2081	L11	187	32	F	Bone	Aneurysmal bone cyst of left humerus superior extremity		-		benign	Lbn030030	0	8
BO2081	L12	188	32	F	Bone	Aneurysmal bone cyst of left humerus superior extremity		-		benign	Lbn030030	0	8
BC000120b	A1	1	59	M	Stomach	Adenocarcinoma	T3N0M0	1	IIA	Malignant	Dst030315	4.5	3
BC000120b	A2	2	55	F	Stomach	Adenocarcinoma	T2N0M0	1	IB	Malignant	Dst030108	6	4
BC000120b	A3	3	51	F	Stomach	Adenocarcinoma	T2N0M0	1--2	IB	Malignant	Dst030474	1	3
BC000120b	A4	4	61	M	Stomach	Adenocarcinoma	T2N0M0	2	IB	Malignant	Dst030387	2	4.5
BC000120b	A5	5	61	M	Stomach	Adenocarcinoma	T2N0M0	2	IB	Malignant	Dst030279	2	2.5
BC000120b	A6	6	60	M	Stomach	Adenocarcinoma	T2N0M0	2	IB	Malignant	Dst030396	3	4.5
BC000120b	A7	7	56	M	Stomach	Adenocarcinoma	T2N0M0	2	IB	Malignant	Dst030278	0	4
BC000120b	A8	8	67	M	Stomach	Adenocarcinoma	T2N0M0	2	IB	Malignant	Dst030306	2	2.5
BC000120b	A10	10	56	F	Stomach	Adenocarcinoma	T3N1M0	2	IIB	Malignant	Dst120185	0	3
BC000120b	A11	11	68	M	Stomach	Adenocarcinoma	T3N0M0	3	IIA	Malignant	Dst030406	2	2.5
BC000120b	A12	12	73	M	Stomach	Adenocarcinoma	T2N0M0	2--3	IB	Malignant	Dst030401	1	7.5
BC000120b	A13	13	58	M	Stomach	Adenocarcinoma	T2N0M0	2--3	IB	Malignant	Dst030149	2	2.5
BC000120b	A14	14	57	M	Stomach	Adenocarcinoma	T2N0M0	2	IB	Malignant	Dst030095	0	5
BC000120b	A15	15	60	M	Stomach	Adenocarcinoma	T2N0M0	2	IB	Malignant	Dst030397	1	3
BC000120b	A16	16	50	F	Stomach	Adenocarcinoma	T3N0M0	2--3	IIA	Malignant	Dst030044	0	3
BC000120b	B1	17	94	M	Stomach	Adenocarcinoma	T2N0M0	2--3	IB	Malignant	Dst030297	2	11
BC000120b	B2	18	59	M	Stomach	Adenocarcinoma	T2N0M0	3	IB	Malignant	Dst030250	0	0
BC000120b	B3	19	66	M	Stomach	Adenocarcinoma	T3N0M0	3	IIA	Malignant	Dst030173	3	3.5
BC000120b	B4	20	48	M	Stomach	Adenocarcinoma	T3N2M0	3	IIIA	Malignant	Dst100288	7	2.5
BC000120b	B5	21	70	M	Stomach	Adenocarcinoma	T2N0M0	3	IB	Malignant	Dst030198	6	2
BC000120b	B6	22	45	F	Stomach	Adenocarcinoma	T4N0M1	3	IV	Malignant	Dst030152	0	5
BC000120b	B7	23	56	M	Stomach	Adenocarcinoma	T2N0M0	3	IB	Malignant	Dst030119	3	2.5
BC000120b	B8	24	32	M	Stomach	Adenocarcinoma	T2N0M0	3	IB	Malignant	Dst030346	0	8.25
BC000120b	B9	25	75	M	Stomach	Adenocarcinoma	T2N0M0	3	IB	Malignant	Dst030114	2	6
BC000120b	B10	26	60	M	Stomach	Mucinous adenocarcinoma	T3N0M0	3	IIA	Malignant	Dst030090	2	2
BC000120b	B11	27	47	F	Stomach	Adenocarcinoma	T3N1M0	3	IIB	Malignant	Dst030089	1	4

TMA	Position	No.	Age	Sex	Organ/Anatomic Site	Pathology diagnosis	TNM	Grade	Stage	Type	Tissue ID.	**IRS SST2**	**IRS SST3**
BC000120b	B12	28	69	M	Stomach	Adenocarcinoma	T2N0M0	3	IB	Malignant	Dst030025	4	3
BC000120b	B13	29	66	M	Stomach	Adenocarcinoma	T2N0M0	3	IB	Malignant	Dst030024	3	4
BC000120b	B14	30	49	M	Stomach	Adenocarcinoma	T2N0M0	3	IB	Malignant	Dst030365	1	4
BC000120b	B15	31	69	M	Stomach	Adenocarcinoma	T3N2M0	3	IIIA	Malignant	Dst100011	2	3
BC000120b	B16	32	40	M	Stomach	Adenocarcinoma	T4N0M0	3	IIB	Malignant	Dst030415	1.5	2.5
BC000120b	C1	33	47	F	Stomach	Adenocarcinoma	T2N0M0	3	IB	Malignant	Dst030266	4.5	4
BC000120b	C2	34	63	M	Stomach	Adenocarcinoma	T3N1M0	3--4	IIB	Malignant	Dst100444	2	2
BC000120b	C3	35	57	M	Stomach	Adenocarcinoma	T3N2M0	3	IIIA	Malignant	Dst100438	5.25	0
BC000120b	C4	36	57	M	Stomach	Adenocarcinoma	T3N1M0	3	IIB	Malignant	Dst100292	3	3
BC000120b	C5	37	62	M	Stomach	Adenocarcinoma	T3N2M0	3	IIIA	Malignant	Dst100091	1	1
BC000120b	C6	38	61	F	Stomach	Adenocarcinoma	T2N0M0	3--4	IB	Malignant	Dst030305	3	10
BC000120b	C7	39	46	M	Liver	Hepatocellular carcinoma	T3N0M0	2	III	Malignant	Dlv030488	4	8
BC000120b	C8	40	65	M	Liver	Hepatocellular carcinoma	T2N0M0	1--2	II	Malignant	Dlv030121	2	3
BC000120b	C9	41	62	F	Liver	Hepatocellular carcinoma	T3N0M0	2	III	Malignant	Dlv030570	1.5	7
BC000120b	C10	42	68	M	Liver	Hepatocellular carcinoma	T3N0M0	2	III	Malignant	Dlv030034	2	8
BC000120b	C11	43	48	F	Liver	Hepatocellular carcinoma	T3N0M0	2	III	Malignant	Dlv030364	1	3.5
BC000120b	C12	44	43	M	Liver	Hepatocellular carcinoma	T3N0M0	2	III	Malignant	Dlv030116	1	10
BC000120b	C13	45	49	M	Liver	Hepatocellular carcinoma	T2N0M0	2	II	Malignant	Dlv030029	2	3
BC000120b	C14	46	49	M	Liver	Hepatocellular carcinoma	T2N0M0	2	II	Malignant	Dlv030538	2	7
BC000120b	C15	47	40	M	Liver	Hepatocellular carcinoma	T3N0M0	1--2	III	Malignant	Dlv030517	1.5	8
BC000120b	C16	48	35	M	Liver	Hepatocellular carcinoma	T3N0M0	2	III	Malignant	Dlv030481	3	7
BC000120b	D1	49	65	F	Liver	Hepatocellular carcinoma	T2N0M0	2	II	Malignant	Dlv030012	0	7
BC000120b	D2	50	64	F	Liver	Hepatocellular carcinoma	T3N0M0	3	III	Malignant	Dlv030417	3	5.25
BC000120b	D3	51	49	F	Liver	Hepatocellular carcinoma	T2N0M0	3	II	Malignant	Dlv030617	2	3
BC000120b	D4	52	70	M	Liver	Hepatocellular carcinoma	T3N0M0	2	III	Malignant	Dlv030572	3	7
BC000120b	D5	53	47	F	Liver	Hepatocellular carcinoma	T3N0M0	-	III	Malignant	Dlv030191	3	3
BC000120b	D6	54	58	M	Liver	Hepatocellular carcinoma	T3N0M0	2	III	Malignant	Dlv030585	4	6
BC000120b	D7	55	52	F	Liver	Hepatocellular carcinoma	T3N0M0	2	III	Malignant	Dlv030497	0	6

TMA	Position	No.	Age	Sex	Organ/Anatomic Site	Pathology diagnosis	TNM	Grade	Stage	Type	Tissue ID.	**IRS SST2**	**IRS SST3**
BC000120b	D8	56	40	M	Liver	Hepatocellular carcinoma	T2N0M0	2	II	Malignant	Dlv030717	6	7.5
BC000120b	D9	57	42	M	Liver	Hepatocellular carcinoma	T4N0M0	2	IIIC	Malignant	Dlv030678	0	8
BC000120b	D10	58	68	F	Liver	Hepatocellular carcinoma	T2N0M0	2	II	Malignant	Dlv030319	7	8
BC000120b	D11	59	52	F	Liver	Hepatocellular carcinoma	T3N0M0	2	III	Malignant	Dlv030362	6	3
BC000120b	D12	60	60	M	Liver	Hepatocellular carcinoma	T3N0M0	2	III	Malignant	Dlv100315	1	4.5
BC000120b	D13	61	55	F	Liver	Hepatocellular carcinoma	T3N0M0	2	III	Malignant	Dlv030211	0	3
BC000120b	D14	62	47	M	Liver	Hepatocellular carcinoma	T3N0M0	2	III	Malignant	Dlv030194	3	8
BC000120b	D15	63	35	M	Liver	Hepatocellular carcinoma	T3N0M0	2	III	Malignant	Dlv030189	4.5	8
BC000120b	D16	64	54	F	Liver	Hepatocellular carcinoma	T2N0M0	2	II	Malignant	Dlv100286	4	11
BC000120b	E1	65	46	F	Liver	Hepatocellular carcinoma	T3N0M0	2--3	III	Malignant	Dlv030285	4	3
BC000120b	E2	66	62	F	Liver	Hepatocellular carcinoma	T3N0M0	3	III	Malignant	Dlv030172	1	3
BC000120b	E3	67	63	M	Liver	Hepatocellular carcinoma	T2N0M0	2	II	Malignant	Dlv030030	3	8
BC000120b	E4	68	41	M	Liver	Hepatocellular carcinoma	T3N0M0	3	III	Malignant	Dlv030759	5	3.5
BC000120b	E5	69	56	M	Liver	Hepatocellular carcinoma	T3N0M0	3	III	Malignant	Dlv030591	5	7
BC000120b	E6	70	46	M	Liver	Hepatocellular carcinoma	T3N0M0	2	III	Malignant	Dlv030579	0	6
BC000120b	E7	71	69	F	Liver	Hepatocellular carcinoma	T3N0M0	3	III	Malignant	Dlv030479	0	3
BC000120b	E8	72	35	M	Liver	Hepatocellular carcinoma	T2N0M0	3	II	Malignant	Dlv030221	2	4
BC000120b	E9	73	52	M	Liver	Hepatocellular carcinoma	T3N0M0	3	III	Malignant	Dlv030580	1.5	3.5
BC000120b	E10	74	38	M	Liver	Hepatocellular carcinoma	T3N0M0	3	III	Malignant	Dlv030419	1	9
BC000120b	E11	75	45	M	Liver	Hepatocellular carcinoma	T2N0M0	3	II	Malignant	Dlv030804	4	5
BC000120b	E12	76	64	M	Liver	Hepatocellular carcinoma	T2N0M0	-	II	Malignant	Dlv030439	4	7
BC000120b	E13	77	48	F	Ovary	Low grade serous adenocarcinoma	T1aN0M0	-	IA	Malignant	Fov030859	3	6
BC000120b	E14	78	24	F	Ovary	High grade serous adenocarcinoma	T1aN0M0	-	IA	Malignant	Fov030183	3	7
BC000120b	E15	79	41	F	Ovary	Low grade serous adenocarcinoma	T1N0M0	-	I	Malignant	Fov030178	3	7
BC000120b	E16	80	48	F	Ovary	Mucinous adenocarcinoma	T1N0M0	1--2	I	Malignant	Fov030376	0	0
BC000120b	F1	81	46	F	Ovary	Mucinous adenocarcinoma	T1N0M0	1--2	I	Malignant	Fov030042	7	6
BC000120b	F2	82	32	F	Ovary	Mucinous papillary cystadenocarcinoma	T1N0M0	1--2	I	Malignant	Fov030469	3	4
BC000120b	F3	83	52	F	Ovary	Mucinous adenocarcinoma	T1aN0M0	2	IA	Malignant	Fov100182	3.5	8

TMA	Position	No.	Age	Sex	Organ/Anatomic Site	Pathology diagnosis	TNM	Grade	Stage	Type	Tissue ID.	**IRS SST2**	**IRS SST3**
BC000120b	F4	84	56	F	Ovary	Mucinous adenocarcinoma	T1bN0M0	3	IB	Malignant	Fov030416	3.5	6
BC000120b	F5	85	57	F	Ovary	High grade serous adenocarcinoma	T1bN0M0	-	IB	Malignant	Fov030236	3	5
BC000120b	F6	86	56	F	Ovary	High grade serous adenocarcinoma	T2N0M0	-	II	Malignant	Fov030109	1	3
BC000120b	F7	87	56	F	Ovary	High grade serous adenocarcinoma	T1bN0M0	-	IB	Malignant	Fov030231	0	7
BC000120b	F8	88	73	F	Ovary	High grade serous adenocarcinoma	T1bN0M0	-	IB	Malignant	Fov030377	2	3
BC000120b	F9	89	49	F	Ovary	High grade serous adenocarcinoma	T1aN0M0	-	IA	Malignant	Fov030405	6	4
BC000120b	F10	90	64	F	Ovary	High grade serous adenocarcinoma	T1N0M0	-	I	Malignant	Fov030816	4	7
BC000120b	F11	91	66	F	Ovary	High grade serous adenocarcinoma	T1cN0M0	-	IC	Malignant	Fov030504	2.5	4
BC000120b	F12	92	39	F	Ovary	High grade serous adenocarcinoma	T2N0M0	-	II	Malignant	Fov030055	3	3
BC000120b	F13	93	64	F	Ovary	High grade serous adenocarcinoma	T1N0M0	-	I	Malignant	Fov100071	8	4
BC000120b	F14	94	69	F	Ovary	High grade serous adenocarcinoma	T2N0M0	-	II	Malignant	Fov030128	0	8
BC000120b	F15	95	55	F	Ovary	High grade serous adenocarcinoma	T1aN0M0	-	IA	Malignant	Fov030898	2	0
BC000120b	F16	96	57	F	Ovary	High grade serous adenocarcinoma	T1aN0M0	-	IA	Malignant	Fov030286	3	6
BC000120b	G1	97	47	F	Ovary	High grade serous adenocarcinoma	T1aN0M0	-	IA	Malignant	Fov030524	2	4.5
BC000120b	G2	98	57	F	Ovary	High grade serous adenocarcinoma	T1bN0M0	-	IB	Malignant	Fov030728	2	2
BC000120b	G3	99	53	F	Ovary	High grade serous adenocarcinoma	T1aN0M0	-	IA	Malignant	Fov030763	1	2
BC000120b	G4	100	57	F	Ovary	High grade serous adenocarcinoma	T3N0M0	-	III	Malignant	Fov030788	0	4
BC000120b	G5	101	58	F	Ovary	High grade serous adenocarcinoma	T1aN0M0	-	IA	Malignant	Fov030379	5	7
BC000120b	G6	102	57	F	Ovary	High grade serous adenocarcinoma	T1cN0M0	-	IC	Malignant	Fov030211	1	3
BC000120b	G7	103	45	F	Ovary	High grade serous adenocarcinoma	T1aN0M0	-	IA	Malignant	Fov030313	1	8
BC000120b	G8	104	55	F	Ovary	High grade serous adenocarcinoma	T1aN0M0	-	IA	Malignant	Fov030343	3	7
BC000120b	G9	105	56	F	Ovary	High grade serous adenocarcinoma	T2N0M0	-	II	Malignant	Fov030124	3	4
BC000120b	G10	106	55	F	Ovary	High grade serous adenocarcinoma	T1bN0M0	-	IB	Malignant	Fov030419	2	4
BC000120b	G11	107	48	F	Ovary	High grade serous adenocarcinoma	T3cN0M0	-	IIIC	Malignant	Fov030120	1	3
BC000120b	G12	108	50	F	Ovary	High grade serous adenocarcinoma	T3N1M0	-	IIIC	Malignant	Fov030110	0	3
BC000120b	G13	109	40	F	Ovary	High grade serous adenocarcinoma	T1N0M0	-	I	Malignant	Fov030074	2	4
BC000120b	G14	110	52	F	Ovary	High grade serous adenocarcinoma	T1cN0M0	-	IC	Malignant	Fov030481	1	8
BC000120b	G15	111	47	F	Ovary	High grade serous adenocarcinoma	T3N0M0	-	III	Malignant	Fov100004	0	0

TMA	Position	No.	Age	Sex	Organ/Anatomic Site	Pathology diagnosis	TNM	Grade	Stage	Type	Tissue ID.	**IRS SST2**	**IRS SST3**
BC000120b	G16	112	48	F	Ovary	Adenocarcinoma	T1N0M0	3	I	Malignant	Fov100158	0	7
BC000120b	H1	113	69	F	Ovary	Adenocarcinoma	T2N1M0	3	II	Malignant	Fov100097	1.5	3
BC000120b	H2	114	54	F	Ovary	Adenocarcinoma	T2N0M0	3	II	Malignant	Fov030981	1	3
BC000120b	H3	115	40	F	Uterus	Endometrioid adenocarcinoma	T2N0M0	1	II	Malignant	Fur030344	0	2
BC000120b	H4	116	40	F	Uterus	Endometrioid adenocarcinoma	T1bN0M0	1	IB	Malignant	Fur030068	0	4
BC000120b	H5	117	61	F	Uterus	Endometrioid adenocarcinoma	T1cN0M0	1	I	Malignant	Fur030367	0	2
BC000120b	H6	118	50	F	Uterus	Endometrioid adenocarcinoma	T1bN0M0	1	IB	Malignant	Fur030806	0	4
BC000120b	H7	119	65	F	Uterus	Endometrioid adenocarcinoma	T1aN0M0	1	I	Malignant	Fur030542	4.5	8
BC000120b	H8	120	62	F	Uterus	Endometrioid adenocarcinoma	T1cN0M0	1	I	Malignant	Fur030114	0	7.5
BC000120b	H9	121	57	F	Uterus	Endometrioid adenocarcinoma	T1cN0M0	2	I	Malignant	Fur031300	6	7
BC000120b	H10	122	65	F	Uterus	Endometrioid adenocarcinoma	T2bN0M0	1	II	Malignant	Fur030766	4	7
BC000120b	H11	123	56	F	Uterus	Endometrioid adenocarcinoma	T1N0M0	2	Ib	Malignant	Fur030015	4	7
BC000120b	H12	124	50	F	Uterus	Endometrioid adenocarcinoma	T1bN0M0	2	IB	Malignant	Fur030880	2	7
BC000120b	H13	125	53	F	Uterus	Endometrioid adenocarcinoma	T1bN0M0	1	IB	Malignant	Fur030912	2	7
BC000120b	H14	126	40	F	Uterus	Endometrioid adenocarcinoma	T2aN0M0	1	II	Malignant	Fur031021	4	8
BC000120b	H15	127	51	F	Uterus	Endometrioid adenocarcinoma	T1bN0M0	1--2	IB	Malignant	Fur030984	0	6
BC000120b	H16	128	52	F	Uterus	Endometrioid adenocarcinoma	T1bN0M0	2	IB	Malignant	Fur031400	0	8
BC000120b	I1	129	40	F	Uterus	Endometrioid adenocarcinoma	T1bN0M0	2	IB	Malignant	Fur030374	3	5
BC000120b	I2	130	64	F	Uterus	Endometrioid adenocarcinoma	T1bN0M0	3	IB	Malignant	Fur030662	1	7
BC000120b	I3	131	62	F	Uterus	Endometrioid adenocarcinoma	T1bN0M0	3	IB	Malignant	Fur030797	0	5
BC000120b	I4	132	61	F	Uterus	Endometrioid adenocarcinoma	T1bN0M0	2	IB	Malignant	Fur031386	2	4
BC000120b	I5	133	58	F	Uterus	Endometrioid adenocarcinoma	TIcN0M0	1--2	I	Malignant	Fur031275	1	2.5
BC000120b	I6	134	35	F	Uterus	Endometrioid adenocarcinoma	T2bN0M0	1--2	II	Malignant	Fur031112	1	2
BC000120b	I7	135	55	F	Uterus	Endometrioid adenocarcinoma	T1cN0M0	2	I	Malignant	Fur030193	3	8
BC000120b	I8	136	65	F	Uterus	Endometrioid adenocarcinoma	T1cN0M0	1--2	I	Malignant	Fur030071	5	9
BC000120b	I9	137	56	F	Uterus	Endometrioid adenocarcinoma	T2aN0M0	2	II	Malignant	Fur030131	0	1
BC000120b	I10	138	68	F	Uterus	Endometrioid adenocarcinoma	T1bN0M0	2	IB	Malignant	Fur030113	2	4.5
BC000120b	I11	139	57	F	Uterus	Endometrioid adenocarcinoma	T1cN0M0	2	I	Malignant	Fur031341	2.5	5

TMA	Position	No.	Age	Sex	Organ/Anatomic Site	Pathology diagnosis	TNM	Grade	Stage	Type	Tissue ID.	**IRS SST2**	**IRS SST3**
BC000120b	I12	140	52	F	Uterus	Endometrioid adenocarcinoma	T1bN0M0	2	IB	Malignant	Fur031347	0	3
BC000120b	I13	141	59	F	Uterus	Endometrioid adenocarcinoma	T1cN0M0	2	I	Malignant	Fur030009	2	7
BC000120b	I14	142	50	F	Uterus	Endometrioid adenocarcinoma	T2bN0M0	3	II	Malignant	Fur030783	8	10
BC000120b	I15	143	30	F	Uterus	Endometrioid adenocarcinoma	T1bN0M0	2	IB	Malignant	Fur030929	0	6
BC000120b	I16	144	57	F	Uterus	Endometrioid adenocarcinoma	T1cN0M0	2--3	I	Malignant	Fur031037	2	9
BC000120b	J1	145	47	F	Uterus	Endometrioid adenocarcinoma	T3aN0M0	3	IIIA	Malignant	Fur030582	0	3
BC000120b	J2	146	45	F	Uterus	Endometrioid adenocarcinoma	T1bN0M0	3	IB	Malignant	Fur030456	1	1
BC000120b	J3	147	48	F	Uterus	Endometrioid adenocarcinoma	T2N0M0	2	II	Malignant	Fur030025	0	0
BC000120b	J4	148	60	F	Uterus	Endometrioid adenocarcinoma	T1cN0M0	3	I	Malignant	Fur031018	7	10
BC000120b	J5	149	54	F	Uterus	Endometrioid adenocarcinoma	T1aN0M0	2	I	Malignant	Fur100042	3	2.5
BC000120b	J6	150	53	F	Uterus	Endometrioid adenocarcinoma	T1cN0M0	3	I	Malignant	Fur030167	0	4
BC000120b	J7	151	48	F	Uterus	Endometrioid adenocarcinoma	T1aN0M0	3	I	Malignant	Fur100255	1	7
BC000120b	J8	152	63	F	Uterus	Endometrioid adenocarcinoma	T1aN0M0	3	I	Malignant	Fur030421	4	5
BC000120b	J9	153	40	M	Upper jaw	Squamous cell carcinoma of left upper jaw	T4N0M0	1	IV	Malignant	Doc010736	3	4
BC000120b	J10	154	52	M	Cheek	Squamous cell carcinoma of cheek	T1N0M0	1	I	Malignant	Doc024254	2	4
BC000120b	J11	155	35	F	Tongue	Squamous cell carcinoma	T2N0M0	1	II	Malignant	Doc022134	4	3
BC000120b	J12	156	70	M	Tongue	Squamous cell carcinoma	T2N0M0	1	II	Malignant	Doc140372	4	4
BC000120b	J13	157	54	M	Tongue	Squamous cell carcinoma	T1N0M0	1	I	Malignant	Doc040411	4	4
BC000120b	J14	158	54	M	Upper jaw	Squamous cell carcinoma of left upper jaw	T3N0M0	1	III	Malignant	Doc030331	1	0
BC000120b	J15	159	50	M	Larynx	Squamous cell carcinoma	T4N0M0	1	IV	Malignant	Rla010107	1	3
BC000120b	J16	160	63	M	Larynx	Squamous cell carcinoma	T1N0M0	2	I	Malignant	Rla030977	1	0
BC000120b	K1	161	65	M	Larynx	Squamous cell carcinoma	T2N0M0	2	II	Malignant	Rla040381	0	5
BC000120b	K2	162	61	M	Larynx	Squamous cell carcinoma	T3N0M0	1	III	Malignant	Rla040023	3	4
BC000120b	K3	163	55	M	Larynx	Squamous cell carcinoma	T2N0M0	1	II	Malignant	Rla040382	0	2
BC000120b	K4	164	56	M	Larynx	Squamous cell carcinoma	T4N0M0	1	IV	Malignant	Rla040143	4.5	4
BC000120b	K5	165	59	M	Lip	Squamous cell carcinoma of upper lip	T1N0M0	1	I	Malignant	Doc040421	6	5
BC000120b	K6	166	81	M	Lip	Squamous cell carcinoma	T2N0M0	2	II	Malignant	Doc020132	5	6
BC000120b	K7	167	60	M	Larynx	Squamous cell carcinoma	T4N0M0	1--2	III	Malignant	Rla030154	1	4

TMA	Position	No.	Age	Sex	Organ/Anatomic Site	Pathology diagnosis	TNM	Grade	Stage	Type	Tissue ID.	**IRS SST2**	**IRS SST3**
BC000120b	K8	168	61	M	Larynx	Squamous cell carcinoma	T3N0M0	2	III	Malignant	Rla030036	2	3
BC000120b	K9	169	64	M	Larynx	Squamous cell carcinoma	T2N0M0	2	II	Malignant	Rla010088	2	6
BC000120b	K10	170	52	M	Larynx	Squamous cell carcinoma	T3N0M0	1--2	III	Malignant	Rla030005	3	3
BC000120b	K11	171	73	M	Larynx	Squamous cell carcinoma	T3N0M0	2	III	Malignant	Rla040177	1	2
BC000120b	K12	172	64	M	Larynx	Squamous cell carcinoma	T4N1M0	2	IVA	Malignant	Rla010072	2.5	6
BC000120b	K13	173	60	M	Larynx	Squamous cell carcinoma	T3N0M0	2	III	Malignant	Rla030103	2.5	6
BC000120b	K14	174	60	M	Larynx	Squamous cell carcinoma	T3N0M0	2	III	Malignant	Rla030161	1	4
BC000120b	K15	175	58	M	Larynx	Squamous cell carcinoma	T3N0M0	2	III	Malignant	Rla040031	1	4
BC000120b	K16	176	49	M	Larynx	Squamous cell carcinoma	T2N0M0	2	II	Malignant	Rla031005	0	4
BC000120b	L1	177	60	M	Larynx	Squamous cell carcinoma	T1N0M0	2	I	Malignant	Rla040385	2	4
BC000120b	L2	178	57	M	Larynx	Squamous cell carcinoma	T2N1M0	1	II	Malignant	Rla011562	1	6
BC000120b	L3	179	57	M	Larynx	Squamous cell carcinoma	T4N0M0	2	IV	Malignant	Rla030003	1	2
BC000120b	L4	180	47	F	Nose	Squamous cell carcinoma of maxillary sinus	T1N0M0	2--3	I	Malignant	Rns031022	2	2.5
BC000120b	L5	181	46	M	Larynx	Squamous cell carcinoma	T3N0M0	2	III	Malignant	Rla020606	0	6
BC000120b	L6	182	60	M	Larynx	Squamous cell carcinoma	T1N0M0	3	I	Malignant	Rla030786	2.5	7
BC000120b	L7	183	68	M	Larynx	Squamous cell carcinoma	T2N0M0	2	II	Malignant	Rla040387	0	1
BC000120b	L8	184	49	M	Gingiva	Squamous cell carcinoma	T4N0M0	2	IV	Malignant	Doc030456	0	3
BC000120b	L9	185	52	M	Larynx	Squamous cell carcinoma	T3N0M0	3	III	Malignant	Rla020618	3	2.5
BC000120b	L10	186	47	M	Nose	Squamous cell carcinoma of sinus	T2N0M0	3	II	Malignant	Rns020614	5	5
BC000120b	L11	187	36	F	Nose	Squamous cell carcinoma of maxillary sinus	T2N0M0	3	II	Malignant	Rns020273	4	3.5
BC000120b	L12	188	74	M	Larynx	Squamous cell carcinoma	T2N0M0	3	II	Malignant	Rla020612	1	4.5
BC000120b	L13	189	62	M	Nose	Squamous cell carcinoma of maxillary sinus	T4N0M0	3	IV	Malignant	Rns040007	4	4
BC000120b	L14	190	63	M	Larynx	Squamous cell carcinoma	T3N0M0	3	III	Malignant	Rla040195	1	4.5
KD2001	A1	1	64	F	Kidney	Clear cell carcinoma	T1aN0M0		I	Malignant	Ukn030115	1	3
KD2001	A2	2	64	F	Kidney	Clear cell carcinoma	T1aN0M0		I	Malignant	Ukn030115	0	4
KD2001	A3	3	50	M	Kidney	Clear cell carcinoma	T1bN0M0		I	Malignant	Ukn030055	0	3.5
KD2001	A4	4	50	M	Kidney	Clear cell carcinoma	T1bN0M0		I	Malignant	Ukn030055	2	4
KD2001	A5	5	70	M	Kidney	Clear cell carcinoma	T1bN0M0		I	Malignant	Ukn030022	6	4.5

TMA	Position	No.	Age	Sex	Organ/Anatomic Site	Pathology diagnosis	TNM	Grade	Stage	Type	Tissue ID.	**IRS SST2**	**IRS SST3**
KD2001	A6	6	70	M	Kidney	Clear cell carcinoma	T1bN0M0		I	Malignant	Ukn030022	6	4
KD2001	A7	7	68	M	Kidney	Clear cell carcinoma	T1aN0M0		I	Malignant	Ukn020069	3	6
KD2001	A8	8	68	M	Kidney	Clear cell carcinoma	T1aN0M0		I	Malignant	Ukn020069	3	5
KD2001	A9	9	74	M	Kidney	Clear cell carcinoma	T1aN0M0		I	Malignant	Ukn020065	3	6
KD2001	A10	10	74	M	Kidney	Clear cell carcinoma	T1aN0M0		I	Malignant	Ukn020065	3	3
KD2001	A11	11	26	M	Kidney	Clear cell carcinoma	T1aN0M0		I	Malignant	Ukn020064	3	7
KD2001	A12	12	26	M	Kidney	Clear cell carcinoma	T1aN0M0		I	Malignant	Ukn020064	3	7
KD2001	A13	13	79	M	Kidney	Clear cell carcinoma	T1bN0M0		I	Malignant	Ukn020062	3	4
KD2001	A14	14	79	M	Kidney	Clear cell carcinoma	T1bN0M0		I	Malignant	Ukn020062	3	7
KD2001	A15	15	61	M	Kidney	Clear cell carcinoma	T2aN0M0		II	Malignant	Ukn020051	2	5
KD2001	A16	16	61	M	Kidney	Clear cell carcinoma	T2aN0M0		II	Malignant	Ukn020051	2.5	5
KD2001	A17	17	45	F	Kidney	Clear cell carcinoma	T1aN0M0		I	Malignant	Ukn020037	2.5	4
KD2001	A18	18	45	F	Kidney	Clear cell carcinoma	T1aN0M0		I	Malignant	Ukn020037	3	4
KD2001	B1	19	66	M	Kidney	Clear cell carcinoma	T1aN0M0		I	Malignant	Ukn020024	5	7.5
KD2001	B2	20	66	M	Kidney	Clear cell carcinoma	T1aN0M0		I	Malignant	Ukn020024	5	4
KD2001	B3	21	49	F	Kidney	Clear cell carcinoma	T1aN0M0		I	Malignant	Ukn020022	3	0
KD2001	B4	22	49	F	Kidney	Clear cell carcinoma	T1aN0M0		I	Malignant	Ukn020022	4.5	3
KD2001	B5	23	50	M	Kidney	Clear cell carcinoma	T1aN0M0		I	Malignant	Ukn020020	2	4
KD2001	B6	24	50	M	Kidney	Clear cell carcinoma	T1aN0M0		I	Malignant	Ukn020020	2	4
KD2001	B7	25	67	M	Kidney	Clear cell carcinoma	T1aN0M0		I	Malignant	Ukn020010	3	6
KD2001	B8	26	67	M	Kidney	Clear cell carcinoma	T1aN0M0		I	Malignant	Ukn020010	1	4
KD2001	B9	27	57	F	Kidney	Clear cell carcinoma	T1aN0M0		I	Malignant	Ukn010147	1.5	7
KD2001	B10	28	57	F	Kidney	Clear cell carcinoma	T1aN0M0		I	Malignant	Ukn010147	1	7
KD2001	B11	29	58	F	Kidney	Clear cell carcinoma	T1aN0M0		I	Malignant	Ukn010132	1	3
KD2001	B12	30	58	F	Kidney	Clear cell carcinoma	T1aN0M0		I	Malignant	Ukn010132	0	4
KD2001	B13	31	49	F	Kidney	Clear cell carcinoma	T1aN0M0		I	Malignant	Ukn010116	0	1
KD2001	B14	32	49	F	Kidney	Clear cell carcinoma	T1aN0M0		I	Malignant	Ukn010116	1	1
KD2001	B15	33	62	M	Kidney	Clear cell carcinoma	T1aN0M0		I	Malignant	Ukn010106	3	7

TMA	Position	No.	Age	Sex	Organ/Anatomic Site	Pathology diagnosis	TNM	Grade	Stage	Type	Tissue ID.	**IRS SST2**	**IRS SST3**
KD2001	B16	34	62	M	Kidney	Clear cell carcinoma	T1aN0M0		I	Malignant	Ukn010106	3	7
KD2001	B17	35	59	M	Kidney	Clear cell carcinoma	T1aN0M0		I	Malignant	Ukn010102	0	5
KD2001	B18	36	59	M	Kidney	Clear cell carcinoma(chronic nephritis)	T1aN0M0		I	Malignant	Ukn010102	3	3
KD2001	C1	37	68	F	Kidney	Clear cell carcinoma	T1aN0M0		I	Malignant	Ukn010101	2	3
KD2001	C2	38	68	F	Kidney	Clear cell carcinoma	T1aN0M0		I	Malignant	Ukn010101	1	2
KD2001	C3	39	67	M	Kidney	Clear cell carcinoma	T3aN0M0		III	Malignant	Ukn010100	3	2
KD2001	C4	40	67	M	Kidney	Clear cell carcinoma	T3aN0M0		III	Malignant	Ukn010100	2.5	3
KD2001	C5	41	66	M	Kidney	Clear cell carcinoma	T1aN0M0		I	Malignant	Ukn010095	0	3
KD2001	C6	42	66	M	Kidney	Clear cell carcinoma	T1aN0M0		I	Malignant	Ukn010095	0	2
KD2001	C7	43	52	M	Kidney	Clear cell carcinoma	T1bN0M0		I	Malignant	Ukn010079	6	5
KD2001	C8	44	52	M	Kidney	Clear cell carcinoma	T1bN0M0		I	Malignant	Ukn010079	6	5
KD2001	C9	45	64	F	Kidney	Clear cell carcinoma	T1bN0M0		I	Malignant	Ukn010068	1	1
KD2001	C10	46	64	F	Kidney	Clear cell carcinoma	T1bN0M0		I	Malignant	Ukn010068	0	2
KD2001	C11	47	73	F	Kidney	Clear cell carcinoma(chronic nephritis)	T1bN0M0		I	Malignant	Ukn010066	6	8
KD2001	C12	48	73	F	Kidney	Clear cell carcinoma(chronic nephritis)	T1bN0M0		I	Malignant	Ukn010066	6	10
KD2001	C13	49	47	M	Kidney	Clear cell carcinoma	T1bN0M0		I	Malignant	Ukn010062	0	5
KD2001	C14	50	47	M	Kidney	Clear cell carcinoma	T1bN0M0		I	Malignant	Ukn010062	1	4
KD2001	C15	51	63	M	Kidney	Clear cell carcinoma	T1aN0M0		I	Malignant	Ukn010043	1	6
KD2001	C16	52	63	M	Kidney	Clear cell carcinoma	T1aN0M0		I	Malignant	Ukn010043	1	5
KD2001	C17	53	45	M	Kidney	Clear cell carcinoma	T2aN0M0		II	Malignant	Ukn010037	1	5
KD2001	C18	54	45	M	Kidney	Clear cell carcinoma	T2aN0M0		II	Malignant	Ukn010037	0	3
KD2001	D1	55	56	M	Kidney	Clear cell carcinoma	T1bN0M0		I	Malignant	Ukn010029	0	3
KD2001	D2	56	56	M	Kidney	Clear cell carcinoma	T1bN0M0		I	Malignant	Ukn010029	1	4
KD2001	D3	57	67	M	Kidney	Clear cell carcinoma	T2bN0M0		II	Malignant	Ukn010027	2	4
KD2001	D4	58	67	M	Kidney	Clear cell carcinoma	T2bN0M0		II	Malignant	Ukn010027	2	5
KD2001	D5	59	50	M	Kidney	Clear cell carcinoma	T1bN0M0		I	Malignant	Ukn010026	4	3
KD2001	D6	60	50	M	Kidney	Clear cell carcinoma	T1bN0M0		I	Malignant	Ukn010026	4	4
KD2001	D7	61	50	M	Kidney	Clear cell carcinoma	T2aN0M0		II	Malignant	Ukn010013	1	0

TMA	Position	No.	Age	Sex	Organ/Anatomic Site	Pathology diagnosis	TNM	Grade	Stage	Type	Tissue ID.	**IRS SST2**	**IRS SST3**
KD2001	D8	62	50	M	Kidney	Clear cell carcinoma	T2aN0M0		II	Malignant	Ukn010013	1	3
KD2001	D9	63	54	M	Kidney	Clear cell carcinoma	T1aN0M0		I	Malignant	Ukn010010	6	10
KD2001	D10	64	54	M	Kidney	Clear cell carcinoma	T1aN0M0		I	Malignant	Ukn010010	6	10
KD2001	D11	65	54	M	Kidney	Clear cell carcinoma	T1aN0M0		I	Malignant	Ukn010003	4	7
KD2001	D12	66	54	M	Kidney	Clear cell carcinoma	T1aN0M0		I	Malignant	Ukn010003	3	7
KD2001	D13	67	59	M	Kidney	Clear cell carcinoma with necrosis	T1aN0M0		I	Malignant	Ukn030090	2	8
KD2001	D14	68	59	M	Kidney	Clear cell carcinoma with necrosis	T1aN0M0		I	Malignant	Ukn030090	3	10
KD2001	D15	69	58	M	Kidney	Clear cell carcinoma(tumor necrosis)	T3aN0N0		III	Malignant	Ukn030089	0	0
KD2001	D16	70	58	M	Kidney	Clear cell carcinoma	T3aN0N0		III	Malignant	Ukn030089	3	11
KD2001	D17	71	60	M	Kidney	Clear cell carcinoma	T1bN0M0		I	Malignant	Ukn030017	0	2.5
KD2001	D18	72	60	M	Kidney	Clear cell carcinoma	T1bN0M0		I	Malignant	Ukn030017	0	3
KD2001	E1	73	67	M	Kidney	Clear cell carcinoma	T1aN0M0		I	Malignant	Ukn020049	2	8
KD2001	E2	74	67	M	Kidney	Clear cell carcinoma	T1aN0M0		I	Malignant	Ukn020049	2.5	9
KD2001	E3	75	60	M	Kidney	Clear cell carcinoma	T1bN0M0		I	Malignant	Ukn020044	0	7
KD2001	E4	76	60	M	Kidney	Clear cell carcinoma	T1bN0M0		I	Malignant	Ukn020044	0	10
KD2001	E5	77	64	F	Kidney	Clear cell carcinoma	T1aN0M0		I	Malignant	Ukn020043	2.5	7
KD2001	E6	78	64	F	Kidney	Clear cell carcinoma	T1aN0M0		I	Malignant	Ukn020043	2	4
KD2001	E7	79	60	M	Kidney	Clear cell carcinoma	T2aN0M0		II	Malignant	Ukn020021	2	6
KD2001	E8	80	60	M	Kidney	Clear cell carcinoma	T2aN0M0		II	Malignant	Ukn020021	3	7
KD2001	E9	81	33	F	Kidney	Clear cell carcinoma	T1bN0M0		I	Malignant	Ukn010133	4.5	3
KD2001	E10	82	33	F	Kidney	Clear cell carcinoma	T1bN0M0		I	Malignant	Ukn010133	3	4
KD2001	E11	83	72	M	Kidney	Clear cell carcinoma	T1aN0M0		I	Malignant	Ukn010125	0	5
KD2001	E12	84	72	M	Kidney	Clear cell carcinoma	T1aN0M0		I	Malignant	Ukn010125	0	6
KD2001	E13	85	50	M	Kidney	Clear cell carcinoma	T1aN0M0		I	Malignant	Ukn010107	3.5	10
KD2001	E14	86	50	M	Kidney	Clear cell carcinoma	T1aN0M0		I	Malignant	Ukn010107	2	3
KD2001	E15	87	62	M	Kidney	Clear cell carcinoma	T1aN0M0		I	Malignant	Ukn010206	2	5
KD2001	E16	88	62	M	Kidney	Clear cell carcinoma	T1aN0M0		I	Malignant	Ukn010206	2.5	6
KD2001	E17	89	60	M	Kidney	Clear cell carcinoma	T2aN0M0		II	Malignant	Ukn010091	3	6

TMA	Position	No.	Age	Sex	Organ/Anatomic Site	Pathology diagnosis	TNM	Grade	Stage	Type	Tissue ID.	**IRS SST2**	**IRS SST3**
KD2001	E18	90	60	M	Kidney	Clear cell carcinoma	T2aN0M0		II	Malignant	Ukn010091	3.5	7
KD2001	F1	91	54	M	Kidney	Clear cell carcinoma(sparse)	T1bN0M0		I	Malignant	Ukn010061	3.5	2
KD2001	F2	92	54	M	Kidney	Clear cell carcinoma	T1bN0M0		I	Malignant	Ukn010061	3.5	3
KD2001	F3	93	74	M	Kidney	Clear cell carcinoma	T2aN0M0		II	Malignant	Ukn010058	0	0
KD2001	F4	94	74	M	Kidney	Clear cell carcinoma	T2aN0M0		II	Malignant	Ukn010058	2	3
KD2001	F5	95	54	F	Kidney	Clear cell carcinoma	T2aN0M0		II	Malignant	Ukn010049	0	2
KD2001	F6	96	54	F	Kidney	Clear cell carcinoma	T2aN0M0		II	Malignant	Ukn010049	6	2
KD2001	F7	97	57	F	Kidney	Clear cell carcinoma	T1aN0M0		I	Malignant	Ukn010048	0	0
KD2001	F8	98	57	F	Kidney	Clear cell carcinoma	T1aN0M0		I	Malignant	Ukn010048	0	0
KD2001	F9	99	71	F	Kidney	Clear cell carcinoma	T2aN0M0		II	Malignant	Ukn010045	2	2
KD2001	F10	100	71	F	Kidney	Clear cell carcinoma	T2aN0M0		II	Malignant	Ukn010045	1	4
KD2001	F11	101	64	F	Kidney	Clear cell carcinoma	T2aN0M0		II	Malignant	Ukn010040	3.5	4
KD2001	F12	102	64	F	Kidney	Clear cell carcinoma	T2aN0M0		II	Malignant	Ukn010040	5	4
KD2001	F13	103	53	F	Kidney	Clear cell carcinoma	T1aN0M0		I	Malignant	Ukn010031	1	2.5
KD2001	F14	104	53	F	Kidney	Clear cell carcinoma	T1aN0M0		I	Malignant	Ukn010031	0	4
KD2001	F15	105	71	F	Kidney	Clear cell carcinoma(tumor necrosis)	T2aN0M0		II	Malignant	Ukn030029	5	8
KD2001	F16	106	71	F	Kidney	Clear cell carcinoma	T2aN0M0		II	Malignant	Ukn030029	4	7
KD2001	F17	107	54	M	Kidney	Clear cell carcinoma	T1bN0M0		I	Malignant	Ukn020054	7	4.5
KD2001	F18	108	54	M	Kidney	Clear cell carcinoma	T1bN0M0		I	Malignant	Ukn020054	6	5.5
KD2001	G1	109	43	M	Kidney	Clear cell carcinoma	T1aN0M0		I	Malignant	Ukn020048	6	7
KD2001	G2	110	43	M	Kidney	Clear cell carcinoma	T1aN0M0		I	Malignant	Ukn020048	1	6
KD2001	G3	111	50	M	Kidney	Clear cell carcinoma	T1aN0M0		I	Malignant	Ukn020029	2	3
KD2001	G4	112	50	M	Kidney	Clear cell carcinoma	T1aN0M0		I	Malignant	Ukn020029	3	5
KD2001	G5	113	53	F	Kidney	Clear cell carcinoma	T2bN0M0		II	Malignant	Ukn020017	3	4
KD2001	G6	114	53	F	Kidney	Clear cell carcinoma	T2bN0M0		II	Malignant	Ukn020017	2	4
KD2001	G7	115	57	F	Kidney	Clear cell carcinoma	T2aN0M0		II	Malignant	Ukn020009	5	5.25
KD2001	G8	116	57	F	Kidney	Clear cell carcinoma	T2aN0M0		II	Malignant	Ukn020009	5	5
KD2001	G9	117	73	M	Kidney	Clear cell carcinoma	T1bN0M0		I	Malignant	Ukn020006	3	6

TMA	Position	No.	Age	Sex	Organ/Anatomic Site	Pathology diagnosis	TNM	Grade	Stage	Type	Tissue ID.	**IRS SST2**	**IRS SST3**
KD2001	G10	118	73	M	Kidney	Clear cell carcinoma	T1bN0M0		I	Malignant	Ukn020006	1	6
KD2001	G11	119	60	M	Kidney	Clear cell carcinoma	T1aN0M0		I	Malignant	Ukn010149	6	7
KD2001	G12	120	60	M	Kidney	Clear cell carcinoma	T1aN0M0		I	Malignant	Ukn010149	3	3
KD2001	G13	121	51	M	Kidney	Clear cell carcinoma	T3aN0M0		III	Malignant	Ukn010113	6	6
KD2001	G14	122	51	M	Kidney	Clear cell carcinoma	T3aN0M0		III	Malignant	Ukn010113	2	6
KD2001	G15	123	65	M	Kidney	Clear cell carcinoma(fiber stroma)	T3aN0M0		III	Malignant	Ukn010096	3	0
KD2001	G16	124	65	M	Kidney	Clear cell carcinoma	T3aN0M0		III	Malignant	Ukn010096	6	6
KD2001	G17	125	78	M	Kidney	Clear cell carcinoma	T3aN0M0		III	Malignant	Ukn010093	2	3.5
KD2001	G18	126	78	M	Kidney	Clear cell carcinoma	T3aN0M0		III	Malignant	Ukn010093	3	5.5
KD2001	H1	127	38	M	Kidney	Clear cell carcinoma	T2aN0M0		II	Malignant	Ukn010060	0	3
KD2001	H2	128	38	M	Kidney	Clear cell carcinoma	T2aN0M0		II	Malignant	Ukn010060	4	5.25
KD2001	H3	129	51	M	Kidney	Clear cell carcinoma	T1bN0M0		I	Malignant	Ukn010052	1	4.5
KD2001	H4	130	51	M	Kidney	Clear cell carcinoma	T1bN0M0		I	Malignant	Ukn010052	5	3
KD2001	H5	131	67	M	Kidney	Clear cell carcinoma	T1aN0M0		I	Malignant	Ukn020164	3	0
KD2001	H6	132	67	M	Kidney	Clear cell carcinoma	T1aN0M0		I	Malignant	Ukn020164	3.5	0
KD2001	H7	133	51	M	Kidney	Clear cell carcinoma	T2aN0M0		II	Malignant	Ukn020041	7	8
KD2001	H8	134	51	M	Kidney	Clear cell carcinoma	T2aN0M0		II	Malignant	Ukn020041	5.25	6.875
KD2001	H9	135	65	M	Kidney	Clear cell carcinoma	T2bN0M0		II	Malignant	Ukn020014	3	3
KD2001	H10	136	65	M	Kidney	Clear cell carcinoma	T2bN0M0		II	Malignant	Ukn020014	0	2
KD2001	H11	137	43	F	Kidney	Clear cell carcinoma	T1bN0M0		I	Malignant	Ukn020004	2	5
KD2001	H12	138	43	F	Kidney	Clear cell carcinoma	T1bN0M0		I	Malignant	Ukn020004	4	6
KD2001	H13	139	74	F	Kidney	Clear cell papillary renal cell carcinoma	T1aN0M0		I	Malignant	Ukn020026	4	10
KD2001	H14	140	74	F	Kidney	Clear cell papillary renal cell carcinoma	T1aN0M0		I	Malignant	Ukn020026	5.25	10
KD2001	H15	141	40	M	Kidney	Clear cell papillary renal cell carcinoma	T1aN0M0		I	Malignant	Ukn020016	3	0
KD2001	H16	142	40	M	Kidney	Clear cell papillary renal cell carcinoma	T1aN0M0		I	Malignant	Ukn020016	3	0
KD2001	H17	143	54	M	Kidney	Sarcomatoid carcinoma	T2N0M0		II	Malignant	Ukn020126	4	10
KD2001	H18	144	54	M	Kidney	Sarcomatoid carcinoma	T2N0M0		II	Malignant	Ukn020126	4	8
KD2001	I1	145	40	F	Kidney	Sarcomatoid carcinoma	T2bN0M0		II	Malignant	Ukn020113	5	7

TMA	Position	No.	Age	Sex	Organ/Anatomic Site	Pathology diagnosis	TNM	Grade	Stage	Type	Tissue ID.	**IRS SST2**	**IRS SST3**
KD2001	I2	146	40	F	Kidney	Sarcomatoid carcinoma	T2bN0M0		II	Malignant	Ukn020113	6	9
KD2001	I3	147	39	F	Kidney	Papillary renal cell carcinoma(II type)	T1N0M0		I	Malignant	Ukn020334	0	4
KD2001	I4	148	39	F	Kidney	Papillary renal cell carcinoma(II type)	T1N0M0		I	Malignant	Ukn020334	0	4.5
KD2001	I5	149	45	F	Kidney	Papillary renal cell carcinoma(I type)	T1N0M0		I	Malignant	Ukn010134	8	10
KD2001	I6	150	45	F	Kidney	Papillary renal cell carcinoma(I type)	T1N0M0		I	Malignant	Ukn010134	8	10
KD2001	I7	151	50	M	Kidney	Chromopobe carcinoma	T2N1M0		III	Malignant	Ukn070016	10	10
KD2001	I8	152	50	M	Kidney	Chromopobe carcinoma	T2N1M0		III	Malignant	Ukn070016	8	10
KD2001	I9	153	61	F	Kidney	Chromopobe carcinoma	T2N0M0		II	Malignant	Ukn010118	0	0
KD2001	I10	154	61	F	Kidney	Chromopobe carcinoma	T2N0M0		II	Malignant	Ukn010118	0	0
KD2001	I11	155	51	M	Renal pelvis	Invasive low grade urothelial carcinoma	T1N0M0		I	Malignant	Ukn020174	4	4
KD2001	I12	156	51	M	Renal pelvis	Invasive low grade urothelial carcinoma	T1N0M0		I	Malignant	Ukn020174	6	4
KD2001	I13	157	80	F	Renal pelvis	Invasive high grade urothelial carcinoma	T1N0M0		I	Malignant	Ukn020160	4	0
KD2001	I14	158	80	F	Renal pelvis	Invasive high grade urothelial carcinoma	T1N0M0		I	Malignant	Ukn020160	8	2
KD2001	I15	159	65	F	Renal pelvis	Invasive high grade urothelial carcinoma	T3N0M0		III	Malignant	Ukn020156	3	7.5
KD2001	I16	160	65	F	Renal pelvis	Invasive high grade urothelial carcinoma(tumor necrosis)	T3N0M0		III	Malignant	Ukn020156	3	6
KD2001	I17	161	80	M	Renal pelvis	Invasive high grade urothelial carcinoma	T1N0M0		I	Malignant	Ukn020053	10	8
KD2001	I18	162	80	M	Renal pelvis	Invasive high grade urothelial carcinoma	T1N0M0		I	Malignant	Ukn020053	7	9
KD2001	J1	163	47	M	Renal pelvis	Invasive high grade urothelial carcinoma	T1N0M0		I	Malignant	Ukn020032	4	5.5
KD2001	J2	164	47	M	Renal pelvis	Invasive high grade urothelial carcinoma	T1N0M0		I	Malignant	Ukn020032	4	4
KD2001	J3	165	44	M	Renal pelvis	Invasive high grade urothelial carcinoma	T1N0M0		I	Malignant	Ukn020025	4	0
KD2001	J4	166	44	M	Renal pelvis	Invasive high grade urothelial carcinoma	T1N0M0		I	Malignant	Ukn020025	6	0
KD2001	J5	167	62	M	Renal pelvis	Invasive high grade urothelial carcinoma	T1N0M0		I	Malignant	Ukn010141	4	9
KD2001	J6	168	62	M	Renal pelvis	Invasive high grade urothelial carcinoma	T1N0M0		I	Malignant	Ukn010141	5	7
KD2001	J7	169	52	M	Renal pelvis	Invasive low grade urothelial carcinoma	T1N0M0		I	Malignant	Ukn030069	6	2
KD2001	J8	170	52	M	Renal pelvis	Invasive low grade urothelial carcinoma	T1N0M0		I	Malignant	Ukn030069	6	2
KD2001	J9	171	75	M	Renal pelvis	Invasive low grade urothelial carcinoma	T1N0M0		I	Malignant	Ukn020158	6	0
KD2001	J10	172	75	M	Renal pelvis	Invasive low grade urothelial carcinoma	T1N0M0		I	Malignant	Ukn020158	6	2
KD2001	J11	173	56	M	Renal pelvis	Invasive low grade urothelial carcinoma	T1N0M0		I	Malignant	Ukn020154	6	3

TMA	Position	No.	Age	Sex	Organ/Anatomic Site	Pathology diagnosis	TNM	Grade	Stage	Type	Tissue ID.	**IRS SST2**	**IRS SST3**
KD2001	J12	174	56	M	Renal pelvis	Invasive low grade urothelial carcinoma	T1N0M0		I	Malignant	Ukn020154	6	3
KD2001	J13	175	49	M	Renal pelvis	Invasive low grade urothelial carcinoma	T1N0M0		I	Malignant	Ukn020124	4	7
KD2001	J14	176	49	M	Renal pelvis	Invasive low grade urothelial carcinoma	T1N0M0		I	Malignant	Ukn020124	3.5	8
KD2001	J15	177	75	F	Renal pelvis	Invasive low grade urothelial carcinoma	T2N0M0		II	Malignant	Ukn020111	2	3
KD2001	J16	178	75	F	Renal pelvis	Invasive low grade urothelial carcinoma	T2N0M0		II	Malignant	Ukn020111	2	6
KD2001	J17	179	60	M	Renal pelvis	Invasive low grade urothelial carcinoma	T1N0M0		I	Malignant	Ukn020042	4	6
KD2001	J18	180	60	M	Renal pelvis	Invasive low grade urothelial carcinoma	T1N0M0		I	Malignant	Ukn020042	6	7
ME2082d	A1	1	40	M	Skin	Malignant melanoma of right chest wall	T4aN0M0		IIB	Malignant	Atc060179	6	8
ME2082d	A2	2	70	F	Parotid gland	Malignant melanoma of left parotid gland	-		-	Malignant	Doc040189	10.5	8
ME2082d	A3	3	50	M	Esophagus	Malignant melanoma	-		-	Malignant	Des030673	6	9
ME2082d	A4	4	64	M	Esophagus	Malignant melanoma	-		-	Malignant	Des050286	4	8
ME2082d	A5	5	73	M	Small intestine	Malignant melanoma	-		-	Malignant	Din090039	2	6
ME2082d	A6	6	71	M	Small intestine	Malignant melanoma	-		-	Malignant	Din024558	10	7
ME2082d	A7	7	64	M	Rectum	Malignant melanoma	-		-	Malignant	Dre070142	1	8
ME2082d	A8	8	42	F	Rectum	Malignant melanoma	-		-	Malignant	Dre061890	10	8
ME2082d	A9	9	67	M	Rectum	Malignant melanoma	-		-	Malignant	Dre062768	4.5	8
ME2082d	A10	10	67	F	Rectum	Malignant melanoma	-		-	Malignant	Dre030138	2.5	6
ME2082d	A11	11	66	F	Rectum	Malignant melanoma (sparse)	-		-	Malignant	Dre080282	9	11
ME2082d	A12	12	47	M	Rectum	Malignant melanoma	-		-	Malignant	Dre062316	3.75	4
ME2082d	A13	13	64	F	Rectum	Malignant melanoma	-		-	Malignant	Dre023410	4	9
ME2082d	A14	14	75	M	Rectum	Malignant melanoma	-		-	Malignant	Dre062643	6	8
ME2082d	A15	15	66	M	Rectum	Malignant melanoma	-		-	Malignant	Dre031567	9	11
ME2082d	A16	16	54	F	Rectum	Malignant melanoma	-		-	Malignant	Dre041097	10.5	10
ME2082d	B1	17	52	F	Rectum	Malignant melanoma	-		-	Malignant	Dre041512	2	10
ME2082d	B2	18	82	F	Rectum	Malignant melanoma of anal canal	T4aN0M0		IIB	Malignant	Dre041111	6	8
ME2082d	B3	19	57	M	Rectum	Malignant melanoma of anal canal	T4aN0M0		IIB	Malignant	Dre061446	5	9
ME2082d	B4	20	84	F	Rectum	Malignant melanoma of anus	T4bN0M0		IIC	Malignant	Dre031701	5	9
ME2082d	B5	21	69	F	Rectum	Malignant melanoma of anal canal (sparse)	T4bN3M1		IV	Malignant	Dre051429	2	7.5

TMA	Position	No.	Age	Sex	Organ/Anatomic Site	Pathology diagnosis	TNM	Grade	Stage	Type	Tissue ID.	**IRS SST2**	**IRS SST3**
ME2082d	B6	22	62	F	Skin	Malignant melanoma of left sole of foot	T4aN0M0		IIB	Malignant	Sst17007	7	11
ME2082d	B7	23	31	M	Skin	Malignant melanoma of left upper arm	T4N1M0		III	Malignant	Sss050228	8	8
ME2082d	B8	24	55	M	Stomach	Malignant melanoma	-		-	Malignant	Dst080193	2	6
ME2082d	B9	25	55	M	Stomach	Malignant melanoma	-		-	Malignant	Dst010327	9	10
ME2082d	B10	26	41	F	Vulva	Malignant melanoma	T4aN0M0		IIB	Malignant	Fvl041282	1	7.5
ME2082d	B11	27	34	F	Vulva	Malignant melanoma	T4aN0M0		IIB	Malignant	Fvl041118	3.5	11
ME2082d	B12	28	45	F	Vulva	Malignant melanoma	T4aN0M0		IIB	Malignant	Fvl032365	6	9
ME2082d	B13	29	38	F	Vulva	Malignant melanoma	T4aN0M0		IIB	Malignant	Fvl050429	5	7
ME2082d	B14	30	62	F	Vulva	Malignant melanoma of vagina	-		-	Malignant	Fvl041203	4	8
ME2082d	B15	31	44	F	Vulva	Malignant melanoma	T4aN0M0		IIB	Malignant	Fvl070221	2	7
ME2082d	B16	32	57	F	Vulva	Malignant melanoma	T4aN0M0		IIB	Malignant	Fvl030086	8	10
ME2082d	C1	33	44	F	Cervix	Malignant melanoma	-		-	Malignant	Fdu030471	3	9
ME2082d	C2	34	62	F	Skin	Malignant melanoma of right foot	T4aN0M0		IIB	Malignant	Kin160005	8	12
ME2082d	C3	35	51	M	Mediastinum	Malignant melanoma	-		-	Malignant	Amd090058	4	10
ME2082d	C4	36	72	F	Skin	Malignant melanoma of waist	T4aN0M0		IIB	Malignant	Lbn100003	12	11
ME2082d	C5	37	50	F	Cerebrum	Malignant melanoma of left cerebrum	T4aN0M0		IIB	Malignant	Nct020139	10	10
ME2082d	C6	38	62	F	Urethra	Malignant melanoma	-		-	Malignant	Uuh020408	3	10
ME2082d	C7	39	62	M	Scrotum	Malignant melanoma	T4bN0M0		IIC	Malignant	Msc020091	0	3
ME2082d	C8	40	46	M	Scrotum	Malignant melanoma	T4aN0M0		IIB	Malignant	Msc030430	2	6
ME2082d	C9	41	55	M	Scrotum	Malignant melanoma	T4N1M0		III	Malignant	Msc080034	2	0
ME2082d	C10	42	64	M	Skin	Malignant melanoma of sole of foot	T4aN0M0		IIB	Malignant	Sst14038	4.5	11
ME2082d	C11	43	78	M	Nose	Malignant melanoma of left nasal cavity	-		-	Malignant	Rns020059	4.5	11
ME2082d	C12	44	38	F	Nose	Malignant melanoma of left nasal cavity	-		-	Malignant	Rns050322	5	6
ME2082d	C13	45	56	F	Maxillary sinus	Malignant melanoma of right maxillary sinus	-		-	Malignant	Rns120111	7	7
ME2082d	C14	46	50	M	Nose	Malignant melanoma	-		-	Malignant	Rns030924	2.5	7
ME2082d	C15	47	63	M	Nose	Malignant melanoma	-		-	Malignant	Rns070033	2.5	10
ME2082d	C16	48	71	F	Skin	Malignant melanoma of right sole of foot	T2aN0M0		IB	Malignant	Kin14017	2.5	11
ME2082d	D1	49	49	F	Skin	Malignant melanoma with necrosis of back	T4aN0M0		IIB	Malignant	Sst050140	3	4.5

TMA	Position	No.	Age	Sex	Organ/Anatomic Site	Pathology diagnosis	TNM	Grade	Stage	Type	Tissue ID.	**IRS SST2**	**IRS SST3**
ME2082d	D2	50	45	F	Skin	Malignant melanoma of left thigh	T4aN0M0		IIB	Malignant	Sst050082	7	9
ME2082d	D3	51	38	F	Rectum	Malignant melanoma	-		-	Malignant	Dre010332	4	8
ME2082d	D4	52	49	M	Skin	Malignant melanoma of left thigh	T4aN0M0		IIB	Malignant	Sst050141	8	10
ME2082d	D5	53	65	M	Skin	Malignant melanoma of right sole of foot	T2bN0M0		IB	Malignant	Kin040084	7.5	11
ME2082d	D6	54	42	F	Skin	Malignant melanoma of right thigh	T4aN0M0		IIB	Malignant	Sst060045	6	10
ME2082d	D7	55	71	M	Skin	Malignant melanoma of right buttock	T4aN0M0		IIB	Malignant	Sst030215	3.5	8
ME2082d	D8	56	67	F	Skin	Malignant melanoma of right armpit	T2aN0M0		IB	Malignant	Str050192	6	10
ME2082d	D9	57	42	F	Skin	Malignant melanoma of left thumb	T4bN0M0		IIC	Malignant	Ssk010143	2	7.5
ME2082d	D10	58	58	F	Skin	Malignant melanoma of left buttock	T4aN0M0		IIB	Malignant	Ssk010147	5	10
ME2082d	D11	59	50	M	Skin	Malignant melanoma of left shoulder	T4aN0M0		IIB	Malignant	Srm040032	6	10
ME2082d	D12	60	7	M	Skin	Malignant melanoma of sacrococcygeal region	T4aN0M0		IIB	Malignant	Srm030016	0	0
ME2082d	D13	61	25	M	Skin	Malignant melanoma of left sole of foot	T4bN0M0		IIC	Malignant	Kin100016	0	11
ME2082d	D14	62	66	M	Skin	Malignant melanoma of sole of foot	T4bN0M0		IIC	Malignant	Kin140034	4	6
ME2082d	D15	63	45	F	Skin	Malignant melanoma of chest wall	T4aN0M0		IIB	Malignant	Kin030056	5	9
ME2082d	D16	64	41	M	Skin	Malignant melanoma of left forearm	T4aN0M0		IIB	Malignant	Kin080030	10.5	11
ME2082d	E1	65	57	M	Skin	Malignant melanoma of left shoulder	T4aN0M0		IIB	Malignant	Kin030109	0	8.25
ME2082d	E2	66	52	M	Skin	Malignant melanoma of left lower abdominal wall	T4bN0M0		IIC	Malignant	Kin060158	4	7
ME2082d	E3	67	56	M	Skin	Malignant melanoma of abdominal wall	T4aN0M0		IIB	Malignant	Kin030042	9	9
ME2082d	E4	68	54	F	Skin	Malignant melanoma of left foot	T4aN0M0		IIB	Malignant	Kin040053	4	8
ME2082d	E5	69	83	F	Skin	Malignant melanoma of right little finger	T4bN0M0		IIC	Malignant	Kin060202	6	8
ME2082d	E6	70	46	F	Skin	Malignant melanoma of thigh	T4bN0M0		IIC	Malignant	Kin020154	6	9
ME2082d	E7	71	38	M	Skin	Malignant melanoma of right sole of foot	T3N1M0		III	Malignant	Kin130007	6	10
ME2082d	E8	72	49	M	Skin	Malignant melanoma of left buttock	T4bN0M0		IIC	Malignant	Kin080057	8	4.5
ME2082d	E9	73	65	M	Skin	Malignant melanoma of scalp	T4aN0M0		IIB	Malignant	Kin030283	10.5	11
ME2082d	E10	74	61	F	Skin	Malignant melanoma of left foot	T4bN0M0		IIC	Malignant	Kin060181	6	10
ME2082d	E11	75	32	F	Skin	Malignant melanoma of right waist (sparse)	T4aN0M0		IIB	Malignant	Kin070094	6	11
ME2082d	E12	76	41	M	Skin	Malignant melanoma of left leg	T4aN0M0		IIB	Malignant	Kin060143	10	10
ME2082d	E13	77	51	M	Skin	Malignant melanoma of back	T4aN0M0		IIB	Malignant	Kin050114	4	6

TMA	Position	No.	Age	Sex	Organ/Anatomic Site	Pathology diagnosis	TNM	Grade	Stage	Type	Tissue ID.	**IRS SST2**	**IRS SST3**
ME2082d	E14	78	42	M	Skin	Malignant melanoma of left heel	T4N2M0		III	Malignant	Kin050012	0	6
ME2082d	E15	79	63	F	Skin	Malignant melanoma of right heel	T4bN0M0		IIC	Malignant	Kin050001	6	7
ME2082d	E16	80	62	F	Skin	Malignant melanoma of right thumb	T4aN0M0		IIB	Malignant	Kin020031	9	11
ME2082d	F1	81	69	M	Skin	Malignant melanoma of sole of foot	T2bN0M0		IB	Malignant	Kin140035	6	10
ME2082d	F2	82	38	F	Skin	Malignant melanoma of left upper arm	T4N1M0		III	Malignant	Kin040110	7	10
ME2082d	F3	83	55	M	Skin	Malignant melanoma of sole of foot	T4aN0M0		IIB	Malignant	Kin060003	3.5	4
ME2082d	F4	84	51	M	Skin	Malignant melanoma of right upper arm	T4N1M0		III	Malignant	Kin060149	10.5	10
ME2082d	F5	85	74	F	Skin	Malignant melanoma of back	T4aN0M0		IIB	Malignant	Kin170009	4	8
ME2082d	F6	86	45	M	Skin	Malignant melanoma of crissum	T4aN0M0		IIB	Malignant	Kin020054	3	8
ME2082d	F7	87	60	M	Skin	Malignant melanoma of left sole of foot	T4bN0M0		IIC	Malignant	Kin130012	1	6
ME2082d	F8	88	71	M	Skin	Malignant melanoma of right groin	T4aN0M0		IIB	Malignant	Kin090021	2	10
ME2082d	F9	89	59	F	Skin	Malignant melanoma of right buttock (sparse)	T1aN0M0		IA	Malignant	Kin050083	7	10
ME2082d	F10	90	66	F	Skin	Malignant melanoma of chest wall	T4aN0M0		IIB	Malignant	Kin030031	4	9
ME2082d	F11	91	43	F	Skin	Malignant melanoma of left leg	T4bN0M0		IIC	Malignant	Kin080006	7	10
ME2082d	F12	92	80	M	Skin	Malignant melanoma of right sole of foot	T4aN0M0		IIB	Malignant	Kin060124	8	7
ME2082d	F13	93	36	M	Skin	Malignant melanoma of right chest wall	T4aN0M0		IIB	Malignant	Kin060059	8	7.5
ME2082d	F14	94	31	M	Skin	Malignant melanoma of scalp	T4aN0M0		IIB	Malignant	Kin040100	3	7
ME2082d	F15	95	46	F	Skin	Malignant melanoma of right thumb	T4aN0M0		IIB	Malignant	Kin060107	5	9
ME2082d	F16	96	60	M	Skin	Malignant melanoma of right buttock	T4aN1M0		III	Malignant	Kin060015	4	11
ME2082d	G1	97	40	M	Skin	Malignant melanoma of right chest wall	T4aN0M0		IIB	Malignant	Kin060017	6	6
ME2082d	G2	98	37	M	Skin	Malignant melanoma of right upper arm	T4aN0M0		IIB	Malignant	Kin090033	7.5	8
ME2082d	G3	99	47	F	Skin	Malignant melanoma of right upper arm	T4aN0M0		IIB	Malignant	Kin030099	2	10
ME2082d	G4	100	43	F	Skin	Malignant melanoma of right upper abdominal wall	T4aN0M0		IIB	Malignant	Sfb050103	5	4.5
ME2082d	G5	101	66	M	Eye	Malignant melanoma of right lower eyelid	T4bN0M0		IIC	Malignant	Vey030004	6	10
ME2082d	G6	102	52	M	Eye	Malignant melanoma o left eye	T4aN0M0		IIB	Malignant	Vey030016	7	8
ME2082d	G7	103	37	M	Eye	Malignant melanoma o left eye	T3N1M0		III	Malignant	Vey090004	2.5	9
ME2082d	G8	104	56	F	Eye	Malignant melanoma o right eye	T3aN0M0		IIA	Malignant	Vey070024	6	2
ME2082d	G9	105	45	F	Eye	Malignant melanoma o right eyeball	T4aN0M0		IIB	Malignant	Vey070022	4	4

TMA	Position	No.	Age	Sex	Organ/Anatomic Site	Pathology diagnosis	TNM	Grade	Stage	Type	Tissue ID.	**IRS SST2**	**IRS SST3**
ME2082d	G10	106	57	M	Eye	Malignant melanoma of right choroid	T4aN0M0		IIB	Malignant	Vey060023	6	10
ME2082d	G11	107	41	F	Eye	Malignant melanoma of right choroid	T4aN0M0		IIB	Malignant	Vey090008	4	2
ME2082d	G12	108	67	M	Eye	Malignant melanoma o right eye	T2aN0M0		IB	Malignant	Vey050015	5	5
ME2082d	G13	109	49	F	Eye	Malignant melanoma o right eyeball	T4aN0M0		IIB	Malignant	Vey020015	7	10
ME2082d	G14	110	35	F	Eye	Malignant melanoma of right choroid	T4aN0M0		IIB	Malignant	Vey040001	6	8
ME2082d	G15	111	62	F	Eye	Malignant melanoma of right choroid	T4aN0M0		IIB	Malignant	Vey040003	4.5	7
ME2082d	G16	112	57	M	Eye	Malignant melanoma of right choroid	T4aN0M0		IIB	Malignant	Vey030029	6	6
ME2082d	H1	113	65	M	Lymph node	Metastatic malignant melanoma of right groin	-		-	Metastasis	Ily020130	7	10
ME2082d	H2	114	38	M	Lymph node	Metastatic malignant melanoma with necrosis of left armpit	-		-	Metastasis	Ily020132	2	10
ME2082d	H3	115	49	M	Lymph node	Metastatic malignant melanoma of left armpit	-		-	Metastasis	Ily040138	2.5	3
ME2082d	H4	116	49	F	Lymph node	Metastatic malignant melanoma with necrosis of neck	-		-	Metastasis	Ily040099	3	4.5
ME2082d	H5	117	55	M	Lymph node	Metastatic malignant melanoma of left groin	-		-	Metastasis	Ily020267	8	9
ME2082d	H6	118	40	F	Lymph node	Metastatic malignant melanoma of right groin	-		-	Metastasis	Ily030025	3.5	8
ME2082d	H7	119	70	M	Lymph node	Metastatic malignant melanoma of armpit	-		-	Metastasis	Ily030182	8	9
ME2082d	H8	120	68	F	Lymph node	Metastatic malignant melanoma of right groin from vulva	-		-	Metastasis	Ily050011	4	10
ME2082d	H9	121	68	M	Lymph node	Metastatic malignant melanoma of neck	-		-	Metastasis	Ily030317	4	10
ME2082d	H10	122	61	F	Lymph node	Metastatic malignant melanoma of right groin	-		-	Metastasis	Ily050048	2	8
ME2082d	H11	123	65	M	Lymph node	Metastatic malignant melanoma of groin	-		-	Metastasis	Ily030379	8	10
ME2082d	H12	124	46	M	Lymph node	Metastatic malignant melanoma of right groin	-		-	Metastasis	Ily010050	3.5	1
ME2082d	H13	125	42	F	Lymph node	Metastatic malignant melanoma of groin	-		-	Metastasis	Ily030646	4	8
ME2082d	H14	126	43	F	Lymph node	Metastatic malignant melanoma of groin	-		-	Metastasis	Ily030647	5	7
ME2082d	H15	127	56	F	Lymph node	Metastatic malignant melanoma of right groin	-		-	Metastasis	Ily030728	4	10
ME2082d	H16	128	56	F	Lymph node	Metastatic malignant melanoma of groin	-		-	Metastasis	Ily030853	8	10
ME2082d	I1	129	63	F	Lymph node	Metastatic malignant melanoma of right groin	-		-	Metastasis	Ily050026	7	10
ME2082d	I2	130	45	M	Lymph node	Metastatic malignant melanoma of right armpit	-		-	Metastasis	Ily040212	7	3
ME2082d	I3	131	60	F	Lymph node	Metastatic malignant melanoma of neck	-		-	Metastasis	Ily040231	8	10
ME2082d	I4	132	29	F	Lymph node	Metastatic malignant melanoma of neck	-		-	Metastasis	Ily040390	7	10
ME2082d	I5	133	31	F	Lymph node	Metastatic malignant melanoma of neck	-		-	Metastasis	Ily040482	7	10

TMA	Position	No.	Age	Sex	Organ/Anatomic Site	Pathology diagnosis	TNM	Grade	Stage	Type	Tissue ID.	**IRS SST2**	**IRS SST3**
ME2082d	I6	134	57	F	Lymph node	Metastatic malignant melanoma of right groin	-		-	Metastasis	Ily080023	1	10
ME2082d	I7	135	60	F	Lymph node	Metastatic malignant melanoma of right groin	-		-	Metastasis	Ily070128	0	7
ME2082d	I8	136	46	F	Lymph node	Metastatic malignant melanoma of right neck	-		-	Metastasis	Ily070107	8	7
ME2082d	I9	137	49	M	Lymph node	Metastatic malignant melanoma of neck	-		-	Metastasis	Ily070098	2	11
ME2082d	I10	138	58	F	Lymph node	Metastatic malignant melanoma of left groin	-		-	Metastasis	Ily060260	1	7
ME2082d	I11	139	68	M	Lymph node	Metastatic malignant melanoma of right armpit	-		-	Metastasis	Ily050136	5	10
ME2082d	I12	140	52	M	Lymph node	Metastatic malignant melanoma of left groin	-		-	Metastasis	Ily060115	4	10
ME2082d	I13	141	53	F	Lymph node	Metastatic malignant melanoma of right groin	-		-	Metastasis	Ily060147	0	4
ME2082d	I14	142	68	F	Lymph node	Metastatic malignant melanoma of left groin	-		-	Metastasis	Ily060148	8	10
ME2082d	I15	143	38	F	Lymph node	Metastatic malignant melanoma of right groin	-		-	Metastasis	Ily060267	6	10
ME2082d	I16	144	48	M	Lymph node	Metastatic malignant melanoma of groin	-		-	Metastasis	Ily120006	6	8
ME2082d	J1	145	44	M	Lymph node	Metastatic malignant melanoma of right armpit	-		-	Metastasis	Ily020004	6	10
ME2082d	J2	146	70	M	Lymph node	Metastatic malignant melanoma of groin	-		-	Metastasis	Ily070095	3	10
ME2082d	J3	147	74	M	Lymph node	Metastatic malignant melanoma of left groin	-		-	Metastasis	Ily130165	0	6
ME2082d	J4	148	47	F	Lymph node	Metastatic malignant melanoma of armpit	-		-	Metastasis	Kin020078	10	9
ME2082d	J5	149	61	M	Lymph node	Metastatic malignant melanoma of right groin	-		-	Metastasis	Kin060180	7.5	9
ME2082d	J6	150	38	M	Lymph node	Metastatic malignant melanoma of armpit	-		-	Metastasis	Kin020090	5	9
ME2082d	J7	151	41	F	Lymph node	Metastatic malignant melanoma of groin	-		-	Metastasis	Kin030275	6	11
ME2082d	J8	152	49	M	Lymph node	Metastatic malignant melanoma of left groin	-		-	Metastasis	Kin060186	3	6
ME2082d	J9	153	45	F	Lymph node	Metastatic malignant melanoma of left chest wall of No.63	-		-	Metastasis	Kin030056	7.5	10
ME2082d	J10	154	58	M	Lymph node	Metastatic malignant melanoma of left groin	-		-	Metastasis	Kin070024	7	11
ME2082d	J11	155	54	M	Lymph node	Metastatic malignant melanoma of left groin	-		-	Metastasis	Kin100002	7.5	8
ME2082d	J12	156	45	F	Lymph node	Metastatic malignant melanoma of spleen	-		-	Metastasis	Isp070017	4.5	7
ME2082d	J13	157	50	M	Lymph node	Metastatic malignant melanoma of paraesophageal lymph node of No.3	-		-	Metastasis	Des030673	2	3.5
ME2082d	J14	158	50	M	Lymph node	Metastatic malignant melanoma of right parparotid lymph node	-		-	Metastasis	Doc062566	4	7
ME2082d	J15	159	55	F	Lymph node	Metastatic malignant melanoma of right occipital lobe	-		-	Metastasis	Nct050132	7	9
ME2082d	J16	160	62	M	Lymph node	Metastatic malignant melanoma of groin of No.39	-		-	Metastasis	Msc020091	0	9
ME2082d	K1	161	39	M	Lymph node	Metastatic malignant melanoma of left femur	-		-	Metastasis	Lbn060075	1	10

TMA	Posit ion	No.	Age	Sex	Organ/Anatomic Site	Pathology diagnosis	TNM	Grade	Stage	Type	Tissue ID.	**IRS SST2**	**IRS SST3**
ME2082d	K2	162	56	M	Lymph node	Metastatic malignant melanoma of left preauricula	-		-	Metastasis	Str030254	4.5	11
ME2082d	K3	163	80	F	Lymph node	Metastatic malignant melanoma of right armpit	-		-	Metastasis	Str030025	1	5
ME2082d	K4	164	72	F	Lymph node	Metastatic malignant melanoma of left groin	-		-	Metastasis	Str030368	5	10
ME2082d	K5	165	62	M	Lymph node	Metastatic malignant melanoma of groin	-		-	Metastasis	Str080050	3	6
ME2082d	K6	166	41	F	Lymph node	Metastatic malignant melanoma of left groin	-		-	Metastasis	Str040126	6	11
ME2082d	K7	167	61	F	Lymph node	Metastatic malignant melanoma of right thigh	-		-	Metastasis	Sst060088	4	10
ME2082d	K8	168	35	F	Lymph node	Metastatic malignant melanoma with necrosis of left armpit	-		-	Metastasis	Sst060003	8	10
ME2082d	K9	169	41	F	Lymph node	Metastatic malignant melanoma of groin	-		-	Metastasis	Sst030376	7	10
ME2082d	K10	170	72	M	Lymph node	Metastatic malignant melanoma of right groin	-		-	Metastasis	Ssk020031	7	4
ME2082d	K11	171	62	M	Lymph node	Metastatic malignant melanoma of left neck	-		-	Metastasis	Ssk020026	3	9
ME2082d	K12	172	48	M	Lymph node	Metastatic malignant melanoma of neck	-		-	Metastasis	Ssk010135	3	8
ME2082d	K13	173	49	M	Lymph node	Metastatic malignant melanoma of pelvic cavity	-		-	Metastasis	Ssk020010	4.5	9
ME2082d	K14	174	63	M	Lymph node	Metastatic malignant melanoma of right neck	-		-	Metastasis	Sft020175	5	4.5
ME2082d	K15	175	71	M	Lymph node	Metastatic malignant melanoma of right armpit	-		-	Metastasis	Scf130002	3	9
ME2082d	K16	176	67	F	Lymph node	Metastatic malignant melanoma of right armpit of No.56	-		-	Metastasis	Str050192	10	10

Table SI-3 - raw data
